# Evidence supports a causal association between allele-specific vitamin D receptor binding and multiple sclerosis among Europeans

**DOI:** 10.1073/pnas.2302259121

**Published:** 2024-02-12

**Authors:** Cameron Adams, Ali Manouchehrinia, Hong L. Quach, Diana L. Quach, Tomas Olsson, Ingrid Kockum, Catherine Schaefer, Chris P. Ponting, Lars Alfredsson, Lisa F. Barcellos

**Affiliations:** a Genetic Epidemiology and Genomics Laboratory, School of Public Health, University of California, Berkeley, CA, USA; b Department of Clinical Neuroscience, Karolinska Institutet, Stockholm, Sweden; c Centre for Occupational and Environmental Medicine, Region Stockholm, Stockholm, Sweden; d MRC Human Genetics Unit, The Institute of Genetics and Cancer, University of Edinburgh, Western General Hospital, Crewe Road, Edinburgh, UK; e Kaiser Permanente Division of Research, Kaiser Permanente Northern California, Oakland, California, USA; f Academic Specialist Center, Stockholm, Sweden; g Centrum for molecular medicine, Karolinska University Hospital, Stockholm, Sweden; h Institute of Environmental Medicine, Karolinska Institutet, Stockholm, Sweden

**Keywords:** BIOLOGICAL SCIENCES, Genetics

## Abstract

Although evidence exists for a causal association between 25-hydroxyvitamin D (25(OH)D) serum levels and multiple sclerosis (MS), the role of variation in vitamin D receptor (VDR) binding in MS is unknown. Here, we leveraged previously identified variants associated with allele imbalance in VDR binding (VDR-binding variant; VDR-BV) in ChIP-exo data from calcitriol-stimulated lymphoblastoid cell lines and 25(OH)D serum levels from genome-wide association studies to construct genetic instrumental variables (GIVs). GIVs are composed of one or more genetic variants that serve as proxies for exposures of interest. Here, GIVs for both VDR-BVs and 25(OH)D were used in a two-sample Mendelian Randomization study to investigate the relationship between VDR binding at a locus, 25(OH)D serum levels, and MS risk. Data for 13,598 MS cases and 38,887 controls of European ancestry from Kaiser Permanente Northern California, Swedish MS studies, and the UK Biobank were included. We estimated the association between each VDR-BV GIV and MS. Significant interaction between a VDR-BV GIV and a GIV for serum 25OH(D) was evidence for a causal association between VDR-BVs and MS unbiased by pleiotropy. We observed evidence for associations between two VDR-BVs (rs2881514, rs2531804) and MS after correction for multiple tests. There was evidence of interaction between rs2881514 and a 25(OH)D GIV, providing evidence of a causal association between rs2881514 and MS. This study is the first to demonstrate evidence that variation in VDR binding at a locus contributes to MS risk. Our results are relevant to other autoimmune diseases in which vitamin D plays a role.

## Introduction

Multiple sclerosis (MS) is more prevalent among people residing in latitudes farther from the equator (1). This has led to the hypothesis that low vitamin D levels are associated with increased MS susceptibility (2). In observational studies, low levels of serum 25-hydroxyvitamin D (25(OH)D) (3) and lack of sunlight exposure (4, 5) are associated with an increased risk of MS, and vitamin D supplements and diets rich in vitamin D (6, 7) are associated with decreased risk of MS. However, the potential for reverse causation and unmeasured confounding factors make it difficult to assess alternative explanations. Studies employing Mendelian Randomization (MR), a method that utilizes genetic variation in genes associated with an exposure or other variables of interest (genetic instrumental variables or GIVs) to estimate a causal association between that exposure and disease(8), strongly support a causal relationship between low serum 25(OH)D levels and increased MS susceptibility (9–11). Many genetic variants used in these MR studies are involved in vitamin D biosynthesis, suggesting that different aspects of the vitamin D pathway, specifically transcription and expression mediated by vitamin D receptor DNA binding, contribute to the relationship between vitamin D and MS (12, 13).

**Figure 1:**
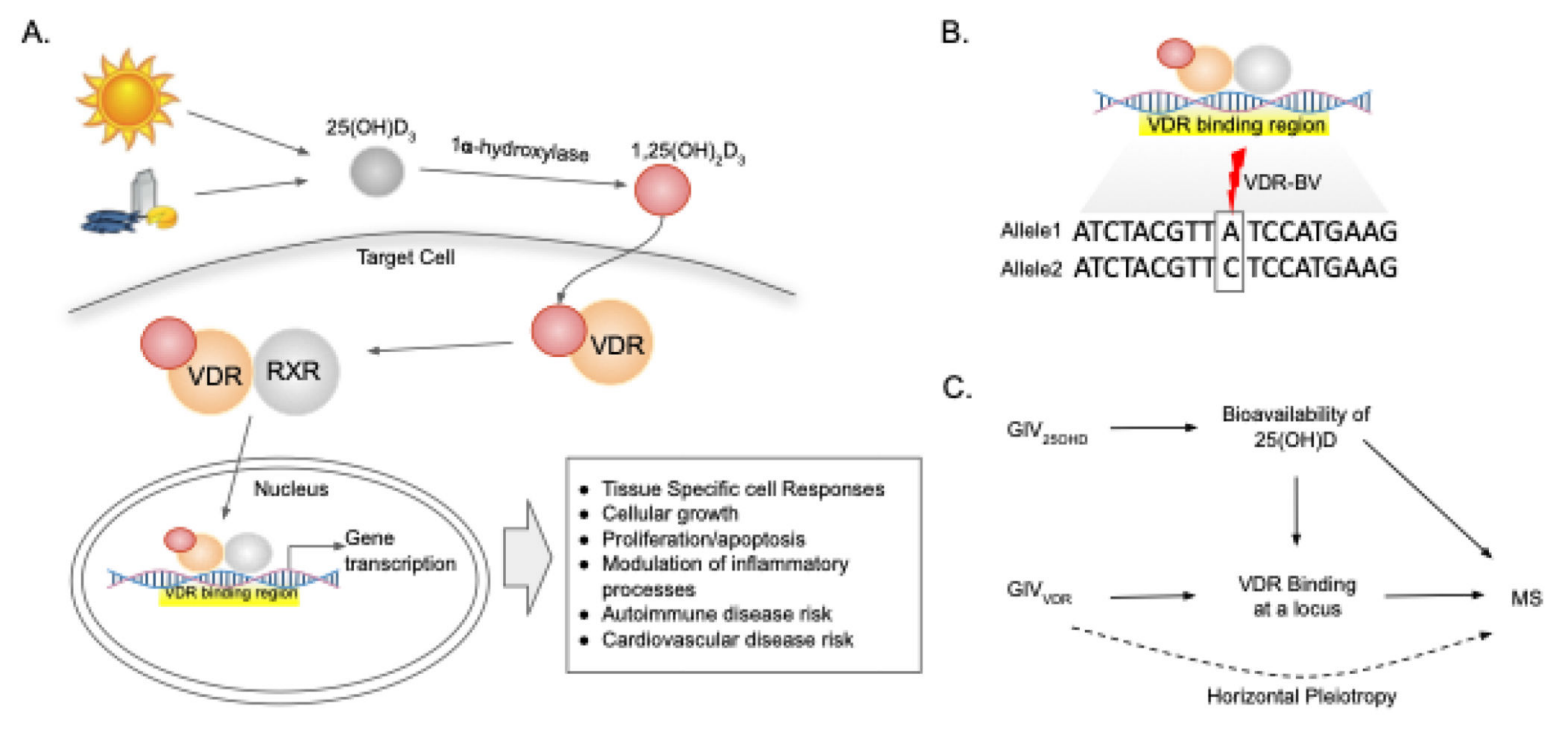
A. Representation of the vitamin D pathway. B. Example of a vitamin D receptor binding variant (VDR-BV) location within a VDR binding region. VDR-BVs are single nucleotide polymorphisms associated with increased or decreased VDR binding at a VDR binding region. C. Directed acyclic graph depicting our mendelian randomization analyses. Genetic variants associated with altered VDR binding at a locus (GIV_VDR-BV_) are used to estimate association between VDR-Binding at a locus and MS susceptibility within our MS case-control datasets. Genetic variants from genome-wide association studies on serum vitamin D levels (GIV_25(OH)D_) are used to estimate the association between VDR binding and MS unbiased by horizontal pleiotropy (dashed-line).

25(OH)D signals through the nuclear vitamin D receptor (VDR), a ligand-regulated transcription factor that mediates all genomic actions of 25(OH)D (Fig. 1A) (14). Upon activation by vitamin D, the VDR forms the RXR/VDR heterodimer that acts as a transcription factor by binding to specific DNA regions across the genome. Most of these regions are vitamin D response elements (VDREs), a particular DNA sequence located in gene promoter regions defined by its high VDR binding affinity; however, not all VDR binding sites contain VDREs (15, 16). Through this pathway, VDR binding modulates the transcription of genes across the genome and 25(OH)D signaling through the VDR can lead to target gene expression (17). Through VDR-mediated gene transcription, vitamin D regulates calcium metabolism, cellular growth, proliferation, apoptosis, and inflammation (18). VDR binds preferentially to one allele over the other at heterozygous sites in Lymphoblastoid Cell-Lines (LCLs) (Fig. 1B), as measured using chromatin immunoprecipitation followed by exonuclease digestion (ChIP-Exo) (19). 1000 Genomes data reveals that VDR-BVs are enriched within genomic regions associated with autoimmune conditions, including MS (19).

Individual VDR-BVs have not been evaluated for association with MS susceptibility in large studies. Here, we leveraged available data on VDR-BVs and results from genome-wide association studies of 25(OH)D serum levels to construct GIVs for both VDR-BV and 25(OH)D serum levels using imputed whole genome SNP data. GIVs can serve as proxies for exposures and other variables of interest (here for VDR-BVs and 25(OH)D) and were used in a two-sample Mendelian Randomization study to investigate the causal relationship between VDR binding at a locus, vitamin D serum levels and MS risk. We also utilized the dependency between VDR binding and bioavailability of 25(OH)D to develop an MR analysis framework to account for horizontal pleiotropy, a source of bias where a GIV (VDR-BV) affects disease risk outside of its effect on the variable of interest. For a true causal association, the bioavailability of 25(OH)D should modulate the effect of genetic variation in VDR binding on MS susceptibility. A significant interaction between the bioavailability of 25(OH)D and VDR binding, captured by the GIVs, would provide evidence of an association between VDR binding at a locus and MS that is not biased by horizontal pleiotropy (Fig.1C). We hypothesized that altered VDR binding modulates transcription and expression of a target gene, thereby increasing or decreasing the risk of MS. We utilized data on individuals from three MS case-control studies and the United Kingdom (UK) Biobank. Identification of VDR-BVs associated with MS could improve understanding of the biological mechanisms through which vitamin D acts to affect MS and further elucidate the molecular and cellular causes of MS.

## Results

### Characteristics of MS cases and controls

The largest source of MS cases was the Swedish Human OMNI (OMNI; 6,709 MS cases and 5,881 controls), followed by the Swedish GSA (GSA; 3,718 MS cases, 1,180 controls), the UK Biobank study (UKB; 2,087 MS cases, 20,870 matched controls), and Kaiser Permanente Northern California (KPNC; 1,082 cases, 10,956 controls) (Table 1). The individuals in Swedish cohorts were genotyped on two different platforms and analyzed separately. Consistent with the female predominance in MS, all studies were primarily comprised of female participants with KPNC having the highest proportion of females (80.2%) and UKB having the lowest (72.4%). The average birth year was earlier within KPNC and UKB studies (the mid-1950s) than the other studies (1960-1970s). The proportion of MS cases with at least one HLA-DRB1*15:01 allele was similar across all studies (~49-57%), with UKB cases having the lowest carriage rate. All analyses were performed separately within KPNC, GSA, OMNI, and UKB and combined with meta-analysis.

### Linkage Disequilibrium between VDR-BVs and MS GWAS risk variants

Within the European samples from the 1000 Genomes project, linkage disequilibrium (LD*)* between VDR-BVs and established MS GWAS variants was generally low. Of the 112 VDR-BVs considered in this investigation, only two VDR-BVs had an *r*^*2*^ > 0.2 with a known MS GWAS variant (VDR-BV: rs55792977, MS GWAS: rs13385171, *r*^*2*^=0.48; VDR-BV: rs13098781, MS GWAS: rs9863496, *r*^*2*^=0.34), indicating no correlation between MS GWAS risk variants and nearly all VDR-BV variants.

### 25(OH)D serum levels are casually associated with MS susceptibility

**Figure 2:**
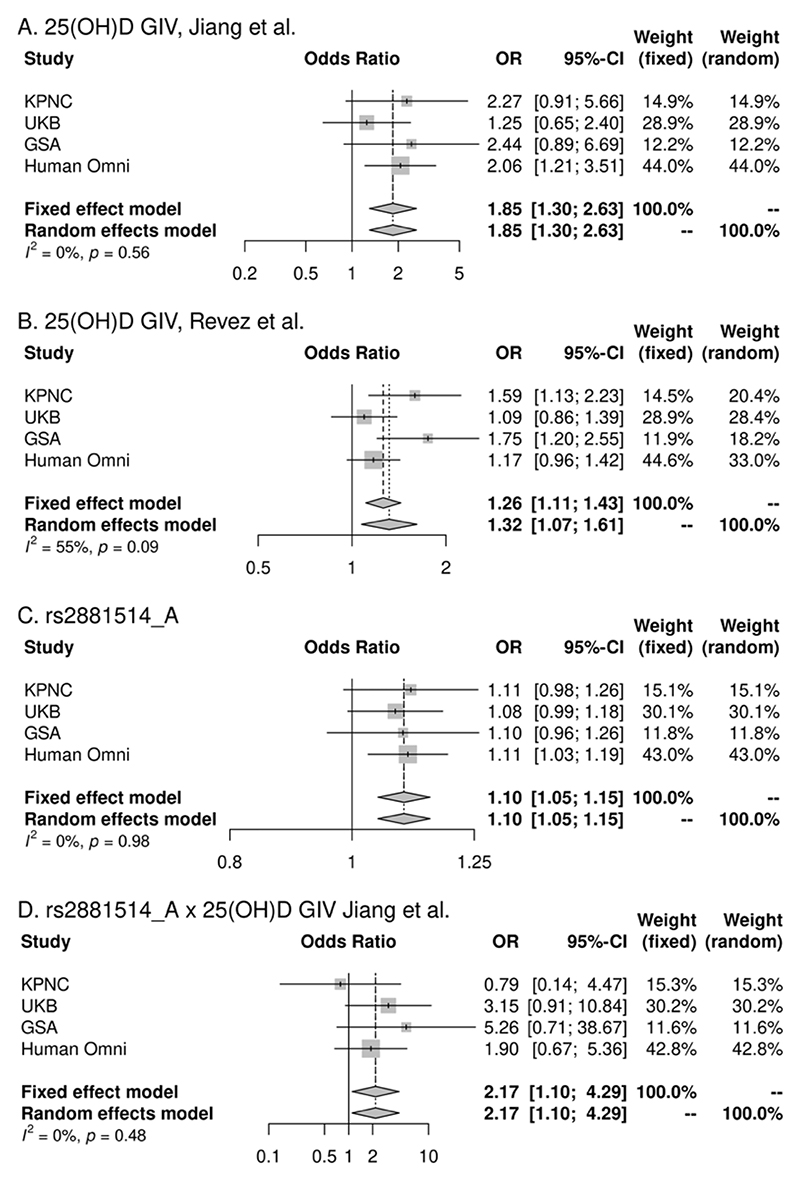
Results from meta-analyses of association between 25(OH)D genetic instrumental variables (GIV_25OHD_) and VDR-BV rs2881514 and multiple sclerosis. GIVs_25OHD_ calculated using summary statistics from genome-wide association studies on serum 25(OH)D levels. A) GIVs_25OHD_ calculated using summary statistics from Jiang et al. 2019 (13); B) GIVs_25OHD_ calculated using summary statistics from Revez et al. 2020 (12). (C) Meta-analysis results for association between GIV_25(OH)D_ rs2881514 and multiple sclerosis and (D) Evidence of interaction between rs2881514 and Jiang et al. 25(OH)D genetic instrumental variable. *τ*^2^= estimate of between study heterogeneity; *I*^2^=proportion of variance due to heterogeneity; and p=p-value for Cochran’s Q test of heterogeneity.

Two GIVs constructed using summary statistics from two recent GWASs on *25(OH)D serum levels by* Jiang et al. and Revez et al. (hereafter, GIV_25OHD_; see Methods) were used in our analyses (12, 13). The mean GIV_25(OH)D_ for increased 25(OH)D serum levels was lower among MS cases compared to controls in all studies (Table 1). The difference for both the Jiang et al. GIV_25(OH)D_ and Revez et al. GIV_25(OH)D_ means between MS cases and controls was the largest among Sweden participants genotyped using the GSA chip. The difference in the means was smallest across both GIV_25(OH)D_ between UKB MS cases and controls. Consistent with previous research, a decreased 25(OH)D serum level captured by each GIV_25(OH)D_ was associated with an increased risk of MS (Fig. 2A-B). The magnitude of the association was greater for the Jiang et al. GIV_25(OH)D_ (OR: 1.85, 95% CI: 1.30-2.63) compared to the Revez et al. GIV_25(OH)D_ (OR: 1.32, 95% CI: 1.07-1.61), likely owing to the difference in the number of SNPs within each GIV (Methods) and reported effect sizes. Estimates for between study variance were low for the Jiang et al. GIV_25(OH)D_ (*I*^2^=0%, *p*_Q_=0.56) indicating associations were consistent across all studies. While there was some evidence for moderate heterogeneity for the Revez et al. GIV_25(OH)D_ (*I*^2^=55%, p_Q_=0.09), the direction of effect was consistent across all studies, providing support for a uniform pattern across the studies, and strong evidence for causal association between low 25(OH)D serum level and MS risk.

### VDR-BV *associations with MS*

GIVs for each VDR-BV (hereafter, GIV_VDR_) were constructed (See Methods). Of the 112 GIVs_VDR_ included in our analyses, seven GIVs_VDR_ were associated with MS at p<0.05 (Table 2). Following correction for multiple testing, two GIV_VDR_ remained significant (p_FDR_<0.05). Meta-analyses of these two GIVs_VDR_ demonstrated evidence of an association between variation within VDR binding and MS susceptibility across all four studies. GIV_VDR_ rs2881514 was associated with an increased risk of MS (Meta-analysis Odds Ratio (OR): 1.10, 95% CI: 1.05-1.15, p=9.4 × 10^−5^; Fig. 2C) and GIV_VDR_ rs2531804 was associated with a decreased risk of MS (Meta-analysis OR: 0.82, 95% CI: 0.73-0.92, p=6.4 × 10^−4^). There was evidence of interaction between 13 GIVs_VDR_ and GIV_25OHD_ at p<0.05 (SI Appendix, Table S1). No interactions were significant after correcting for multiple testing; however, GIV_VDR_ rs2881514, the top finding from the independent models, was among these 13 (GIV_VDR_ rs2881514 × Jiang et al. GIV_25OHD_ Meta-analysis OR: 2.17, 95% CI: 1.10-4.29, p=0.025; Fig. 2D). The direction of this association provided evidence of interaction between *increased* VDR binding at GIV_VDR_ rs2881514 and *decreased* bioavailability of 25(OH)D measured with Jiang et al. GIV_25OHD_ conferring *increased* risk of MS.

### VDR-BVs with evidence of MS association are eQTLs and sQTLs

Several of the GIV_VDR_ that were independently associated at p<0.05 with MS or showed evidence of interaction with GIV_25(OH)D_ are expression quantitative trait loci (eQTL) or splicing quantitative trait loci (sQTL) for one or more human tissues in GTEx (SI Appendix, Fig. S1) (20). GIV_VDR_ rs2881514 is an eQTL for *RFTN1* in esophageal tissue and an sQTL for *RFTN1* in EBV-transformed lymphocytes and thyroid tissues. *RFTN1* encodes Raftlin, a B cell-specific major raft protein necessary for lipid raft integrity and B-cell receptor signal transduction (21). Other GIV_VDR_, including rs2531804, rs55811049, rs961320, rs871699, and rs62200158, which showed evidence of association with MS at p<0.05, are eQTLs or sQTLs in brain or skin tissue, among others.

## Discussion

This is the first study to observe evidence for associations between variation in VDR binding at a locus and MS susceptibility, providing further evidence of the important role of the vitamin D pathway in MS. Our results support the hypothesis that alterations in VDR binding and subsequent modulations in vitamin D gene transcription and expression contributes to developing MS. We found two GIV_VDR_, rs2881514 and rs2531804, were independently associated with MS susceptibility. There was also evidence of interaction between several GIVs_VDR_ and GIV_25(OH)D_ and MS risk. Although no observed interactions were significant after correction for multiple testing, there was evidence of interaction between GIV_VDR_ rs2881514 and the Jiang et al. GIV_25(OH)D_ at p<0.05. It is important to note that detecting interactions requires substantially more statistical power than main effects. Regardless, evidence of an association between a GIV_VDR_ and MS in independent and interaction models is promising (22). Both GIV_25(OH)D_ for 25(OH)D serum levels were significantly associated with MS; specifically, lower 25(OH)D serum was associated with increased risk of MS in all four studies.

There are causal associations between vitamin D serum levels and MS risk using MR methods (9–11). Our findings using GIVs constructed using summary statistics from two recent GWASs on 25(OH)D serum levels are consistent with previous MR studies and further confirm vitamin D insufficiency as an important risk factor for MS. Measures of heterogeneity indicate little variation between studies for Jiang et al. GIV_25(OH)D_, although there was some evidence of moderate heterogeneity for the Revez et al. GIV_25(OH)D_ (*I*^2^=55%, *p*_Q_=0.09), which might be explained by the larger number of SNPs included in the Revez et al. GIV_25(OH)D_. Associations were modest for both GIV_25(OH)D_ among UKB participants. Unlike the other studies, MS cases from UKB did not have their MS status confirmed by a neurologist. Instead, diagnoses were inferred from electronic health records or self-report and may have resulted in a small amount of misclassification of MS case status in this cohort. Evidence of this may be seen in the lower frequency of carriage of HLA-DRB1*15:01, the major risk allele for MS risk, in UKB MS cases (49.7%) compared to cases in the other three studies (>53%).

The top GIV_VDR_ associated with MS from meta-analyses was rs2881514. The A allele of this SNP was associated with increased VDR binding in LCLs. Evidence from results within each study and meta-analyses indicate a harmful effect of increased VDR binding at that locus on MS risk. Further, the interaction observed between GIV_VDR_ rs2881514 and GIV_25(OH)D_ demonstrates evidence for interaction between *increased* VDR binding at rs2881514 and *decreased* 25(OH)D serum levels, yielding a substantial increase in the risk of MS (Meta-analysis OR: 2.17, 95% CI: 1.10-4.29). This association was consistent among UKB, OMNI, and GSA MS cases and controls but was slightly protective in KPNC, though very wide confidence intervals were observed (KPNC OR: 0.79, 95% CI: 0.14-4.47). GIV_VDR_ rs2881514 is located on chromosome 3, 1,533 bases downstream of the transcription start site of *RFTN1. In GTEx*, rs2881514 is an eQTL for *RFTN1* in esophageal tissue and an sQTL for *RFTN1* in EBV-transformed lymphocytes and thyroid tissues, providing evidence for a role of rs2881514 in *RFTN1* expression. *RFTN1* encodes Raftlin, a protein that is critical for producing lipid rafts, which are membrane microdomains that play a crucial role in B cell activation through B cell receptor signaling (21). Raftlin is essential for clathrin-dependent endocytosis of Toll-like Receptor (TLR) 3 ligand in human epithelial cells and myeloid dendritic cells (23), and for LPS-induced TLR4 internalization and Toll-IL-1R domain-containing adaptor molecule-1 (TICAM-1) signaling in human monocyte-derived DCs and macrophages (24). TLR3 and TLR4 signaling pathways have been implicated in MS pathogenesis and symptom modulation (25). In mice, experimental autoimmune encephalomyelitis (EAE), a Th1-/Th17-mediated disease, was more severe among mice with wild-type Raftlin expression compared to Raftlin deficient mice (23). *RFTN1* has not been directly implicated in MS risk; however, there is increased expression of *RFTN1* in chronic active brain lesions from MS cases compared to healthy brain tissue from controls (26). More work is needed to understand the relationship between *RFTN1* and vitamin D, and its role in MS.

Several of the GIV_VDR_ associated with MS had evidence of SNP-associated tissue-specific expression and alternative splicing in GTEx (SI Appendix, Fig. S1). rs25318104, rs9621320, rs871699, rs689384, rs62200158, rs558110449, and rs2286576 show evidence of being an eQTL or sQTL in one or more brain tissues. Many of these SNPs are eQTLs or sQTLs across various tissues. Of the set of VDR-BVs tested as GIV_VDR_ in the current study, those with evidence of association with MS susceptibility were enriched for genes involved in immune-related processes (leukocyte-mediated immunity, immune effector process, myeloid leukocyte activation, and phospholipase activity), vesicles, and exocytosis (SI Appendix, Table S2). Annotations to eQTLs and sQTLs and the enrichment results provide evidence supporting a mediating role of allele-specific VDR binding in the expression of nearby genes and immune pathways in MS risk.

Observational studies have shown that increased dietary intake of vitamin D is associated with reduced risk of MS, but it is unknown whether interventional vitamin D supplementation would yield protective effects on disease susceptibility (6, 27). Studies investigating the effects of vitamin D supplementation on MS progression have found associations between higher serum levels of vitamin D and reduced hazard of relapse and a reduction in incidence of T2 lesions (28, 29). Our results show how genetic variation in the vitamin D pathway affects MS disease risk. Future studies investigating the protective effects of vitamin D supplementation on disease risk and progression should consider genetic variation in vitamin D pathway genes as part of their design.

This study had several strengths. It is the first to investigate the association of VDR binding at a locus with MS among cases and controls. An investigation of variation in VDR binding among cases and controls would not be feasible in large sample sizes without utilizing MR methodology. We used previously identified VDR-BVs to create GIVs for VDR binding at a locus (19). Another strength was the large sample size and use of data from independent studies. There were also some limitations, including the possibility of horizontal pleiotropy, biasing associations between GIV_VDR_ and MS. Each GIV_VDR_ was constructed using only one VDR-BV, preventing the use of standard sensitivity analyses for assessing the validity of MR assumptions in our VDR-BV analyses. However, our use of GIVs for 25(OH)D serum level in the analyses allowed us to estimate the association between GIV_VDR_ and MS unbiased by horizontal pleiotropy. Additionally, all but two of the VDR-BV were independent of known MS GWAS variant risks. MS cases and controls in the study were of European ancestry to reduce the impact of population stratification; however, this approach limits the generalizability of our findings to non-European populations. Further, VDR-BVs were identified in LCLs which are EBV-transformed B-cell lines. Variation in VDR binding associated with MS likely occurs in other lymphocytes, including CD4+ and CD8+ T cells and it is unknown whether the VDR-BVs active in LCLs are also active in these T cells. We were also only able to assess variation in VDR binding at 112 loci, which represent a small portion of genomic regions where VDR binds. Lastly, there are likely to be mechanisms other than VDR binding that underlie the observed VDR-MS associations reported here, including alternative splicing, effect of microRNAs, and combinatorial protein binding. Our study was designed to identify causal mechanisms of MS susceptibility that are mediated by altered VDR binding due to DNA variation.

This study is the first to demonstrate that genetic variation in VDR binding at a single locus contributes to MS susceptibility. Our results highlight the importance of the vitamin D pathway in MS pathogenesis. Our results are also relevant to other autoimmune and inflammatory diseases and several cancers for which a role for vitamin D has been suggested. Future studies of VDR binding and MS should identify VDR-BVs in lymphocytes not captured by LCLs, including CD4+ and CD8+ T cells.

## Materials and Methods

### MS case-control studies

Individual-level case-control data used in this research was from one USA-based case-control study and two Sweden-based MS case-control studies. MS cases and controls from the US were from the Kaiser Permanente Northern California (KPNC) MS Research Program (30). Additional KPNC controls were participants of the Genetic Epidemiology Research on Adult Health and Aging (GERA; dbGaP phs000674.v2. p2) (31). From Sweden, MS cases and controls were from the Nationwide Epidemiological Investigation of Multiple Sclerosis (EIMS), the Genes and Environment in Multiple Sclerosis (GEMS), IMSE study of the effect of immunomodulatory drugs, and the Karolinska Hospital STOPMS study (32, 33). Methods for identification and confirmation of MS diagnosis in each study are provided within the corresponding publications. Briefly, MS cases from KPNC, EIMS, GEMS, IMSE, and STOPMS had their disease status confirmed by an independent neurologist. All study participants provided written informed consent, and all studies obtained approval from the Institutional Review Boards of KPNC, local Ethical Committees, and the University of California, Berkeley.

### UK Biobank

The UK Biobank (UKB, http://www.ukbiobank.ac.uk) is a prospective cohort study of approximately 500,000 individuals from the United Kingdom (34). Recruitment took place between 2006-2010 in 22 assessment centers located across the United Kingdom. Participants were aged 40-69 at the time of recruitment. MS cases were identified using UKB field 31043-0.0 “Source of report of G35 (multiple sclerosis)”. Controls were defined as those not reporting MS symptoms or other demyelinating diseases at the time of enrollment and were frequency matched to cases by year of birth (±2 years) and sex at a ratio of 10 controls per MS case. Participants included in analyses were unrelated individuals of European ancestry with imputed genetic data and no sex chromosome aneuploidies. Those who had withdrawn consent or were recommended for genetic analysis exclusion were excluded. Data were accessed under the approval of UKB within project 69668. All participants gave prior written informed consent, and the study was conducted following the principles of the Declaration of Helsinki.

### Genotype and exposure assessment

All participants in the MS case-control studies (KPNC, GEMS, EIMS, IMSE, and STOP-MS) completed an interview or self-reported questionnaire related to MS disease events, reproductive history, and environmental exposures (30–33). Participants provided blood or saliva samples for genotyping. SNP genotyping was performed using the Illumina Infinium 660K BeadChip Array and Human Omni Express Array (KPNC), Illumina Global Screening Array or Human Omni Expression (GEMS, EIMS, IMSE, and STOPMS), and Affymetrix Axiom Array (GERA). The data from GEMS, EIMS, IMSE, and STOPMS were analyzed in two cohorts depending on which genotyping array was used: Human Omni Express (OMNI) or Global screening array (GSA). Details of genotyping and imputation for KPNC (35), GSA AND OMNI (36), and UKB have been described previously. Participants with missing genotypes that met QC thresholds (info score>0.8, missingness per SNP<0.05, missingness per cohort<0.05, and minor allele frequency (MAF)>0.05) were imputed using the mean MAF within each study.

### Genetic instruments for VDR binding

Mendelian Randomization (MR) is a method that uses one or more genetic variants associated with an exposure or other variable of interest as a genetic instrumental variable (GIV) to estimate causal associations between that exposure and an outcome. GIVs are calculated by summing the weighted contributions of individual SNPs, based on their effect sizes derived from GWASs or other genetic studies. In this study GIV_VDR-BV_ for each individual were constructed using the following equation:

$$GIV_{VDR\text{-}BV_{ij}}=dosage_{ij}\times \beta_{\text{VDR-B}{\text{V}}_{j}}$$

where, *dosage*_*ij*_ is the number of effect alleles at *VDR*-*BV*_*j*_ carried by individual *i* and ${\text{β}}_{\text{VDR-B}{\text{V}}_{\;\text{i}}}$ is the effect size for the association between VDR-binding at a locus and alleles at *VDR*-*BV*_*j*_. There are three primary MR assumptions: the variant(s) must be associated with the exposure, there are no unmeasured confounders of the association between the genetic variant(s) and the outcome, and the genetic variants only affect the outcome through the exposure (8). The occupancy of VDR at a given genomic locus was the exposure of interest in this study. Our objective was to use SNPs associated with VDR binding at a locus previously identified by Gallone et al (37) as GIVs to identify associations between variation in VDR binding at a locus and MS susceptibility. Occupancy of VDR was quantified genome-wide at heterozygous sites using ChIP-exo sequencing read data from sixteen genotyped calcitriol-stimulated LCLs. To satisfy the first assumption of MR, at least one variant statistically associated with altered VDR occupancy at a locus was required (8). SNPs associated with VDR binding in cis were identified by observing differential VDR occupancy over each allele of a given heterozygous SNP. Allele-specific VDR binding was identified using AlleleSeq, modelling reads mapping to each allele using the binomial distribution and applying a two-tailed test (38). VDR-BVs were called at heterozygous sites with five or more ChIP-Exo reads, with at least read being mapped to each allele in order to reduce unwanted effects of PCR duplication. After accounting for copy number variation (32) and correction for multiple testing using AlleleSeq’s computational simulation approach (False discovery rate < 2%), 305 VDR-BVs were identified. Of these, 112 VDR-BVs were present in all imputed case-control datasets for analysis. SNP-VDR binding effect sizes were estimated by regressing the read counts from ChIP-exo data at a heterozygous VDR binding region described above against the corresponding VDR-BV alleles. For each VDR-BV the GIV was derived by multiplying the SNP-VDR binding effect size by the number alleles carried by each participant (hereafter, GIV_VDR_).

### Pleiotropy and Bioavailability of Vitamin D

The causal effect estimates from MR analysis are unbiased in the absence of pleiotropic effects. A common approach to estimating the pleiotropic bias is based on over-dispersion across multiple instrumental SNPs for a given exposure. However, sensitivity analyses for multi-SNP MR instruments were not applicable as there was only one instrumental SNP for each GIV_VDR_. Given that VDR binding is dependent upon the bioavailability of vitamin D, we used variants and summary statistics from two recent GWAS on serum 25(OH)D to construct GIVs for bioavailability of vitamin D (see below) to perform subgroup analyses (12, 13). Interaction between a GIV_VDR_ and bioavailability of 25(OH)D was considered evidence of an association between VDR binding and MS susceptibility not biased by horizontal pleiotropy.

### Genetic instruments for 25(OH)D

SNPs and the estimated effect sizes from two recent GWAS on serum 25(OH)D were used to construct each GIV_25OHD_ (12, 13). GWAS summary statistics were extracted from MR-Base R platform (39). The extract_instruments() function in the TwoSampleMR R package was used to identify independent variants with GWAS *p* < 1 × 10^−8^ within genomic windows of 10,000Kb (R^2^<0.001) using a European LD reference panel from 1000 Genomes Phase 3. These procedures yielded 8 SNPs for the Jiang et al. GIV_25(OH)D_ and 103 SNPs for the Revez et al. GIV_25(OH)D_ (SI Appendix, Tables S3 and S4). After identifying these SNPs, each GIV_25(OH)D_ was calculated in Plink1.9 using --score (40) which uses the following equation:

$$GIV_{25(OH)D_{i}}=\overset{K}{\underset{k}{\sum \;}}\beta_{k}\times \;dosage_{\;ik}$$

where, *dosage*_*ik*_ is the number of effect alleles at GWAS variant *k* carried by individual *i* and *β*_*k*_ is the effect size for the association between reduced 25(OH)D serum levels at and GWAS variant *k*.

### Statistical methods

Logistic regression was used to estimate the associations between all GIVs (GIV_25OHD_ and GIV_VDR_) and MS susceptibility within each study:

$$\begin{array}{c} logit(P(MS=1))\sim \beta_{0}+\beta_{1}GIV_{25OHD}\\ logit(P(MS=1))\sim \beta_{0}+\beta_{1}GIV_{VDR_{j}} \end{array}$$

Additionally, to estimate an association not biased by horizontal pleiotropy, we estimated interaction between each GIV_VDR_ and GIV_25OHD_ for MS susceptibility:

$$logit(P(MS=1))\sim \beta_{0}+\beta_{1}GIV_{VDR_{j}}+\beta_{2}GIV_{25OHD}+\beta_{3}\left(GIV_{VDR_{j}}\times GIV_{25OHD}\right),$$

where *β*_3_ is the estimate of multiplicative interaction between VDR binding at a locus and the bioavailability of 25(OH)D. A significantly non-zero value of *β*_3_ indicates that pleiotropy does not explain all the observed association and therefore, the association between VDR-BV and MS is causal.

All models were adjusted for sex (male or female), quintiles of birth year, carriage of the HLA-DRB1*15:01 allele, and the first six genome-wide principal components. Random effects meta-analysis was used to combine study-specific associations. All meta-analyses were performed using the metagen() function in the Meta R package (41). The DerSimonian-Laird estimator was used to estimate the between-study variance. Between study heterogeneity was assessed using Cochran’s *Q* statistic and Higgins & Thompson’s *I*^2^ statistic. p-values from associations between each GIV_25OHD_ and MS and the interaction parameter between each GIV_VDR_ and GIV_25OHD_ were corrected for multiple tests using the Benjamini-Hochberg method (42).

Annotation of VDR-BVs to the nearest gene transcription start sites (TSS) and GO enrichment analysis was performed using rGREAT (43). Tissue-specific expression quantitative trait loci (eQTL) and splicing quantitative trait loci (sQTL) for VDR-BVs were from the GTEx v8 Project (20). LD between VDR-BVs and MS GWAS risk variants was estimated using the ld_matrix() function from the *ieugwasr* R package using the European LD reference panel from 1000 Genomes Phase 3 (44).

All statistical analyses were conducted using R 4.0.2 (45).

## Supplementary Material

Appendix 1

## Figures and Tables

**Table 1:** Characteristics of MS cases and controls.

	KPNC		UKB		GSA		OMNI	
	Case	Control	Case	Control	Case	Control	Case	Control
n	1084	10956	2087	20870	3718	1180	6709	5881
Sex, n (%)								
Male	215 (19.8)	2165 (19.8)	576 (27.6)	5760 (27.6)	1031 (27.7)	342 (29.0)	1808 (26.9)	1455 (24.7)
Female	869 (80.2)	8791 (80.2)	1511 (72.4)	15110 (72.4)	2687 (72.3)	838 (71.0)	4901 (73.1)	4426 (75.3)
Birth year, mean (SD) DRB1*15:01 carrier, n (%)	1957.6 (8.9)	1957.1 (8.6)	1952.7 (7.6)	1952.7 (7.6)	1973.1 (12.4)	1968.9 (13.7)	1961.0 (13.7)	1960.5 (13.3)
0 alleles	506 (46.7)	8280 (75.6)	1050 (50.3)	15271 (73.2)	1621 (43.6)	843 (71.4)	2913 (43.4)	4052 (68.9)
1 or 2 alleles	578 (53.3)	2676 (24.4)	1037 (49.7)	5599 (26.8)	2097 (56.4)	336 (28.5)	3796 (56.6)	1829 (31.1)
25(OH)D GIV, Jiang et al.^a ^ (mean (SD))	0.285 (0.07)	0.288 (0.07)	0.292 (0.07)	0.293 (0.07)	0.289 (0.07)	0.293 (0.07)	0.286 (0.07)	0.290 (0.07)
25(OH)D GIV, Revez et al.^a^ (mean (SD))	2.150 (0.12)	2.163 (0.19)	2.184 (0.19)	2.187 (0.19)	2.057 (0.19)	2.074 (0.18)	2.066 (0.19)	2.070 (0.19)

Abbreviations: GSA, Swedish cohort genotyped on global screening array; Human Omni, Swedish cohort genotyped on human omni express array; KPNC, Kaiser Permanente Northern California MS case-control study; GIV, Genetic Instrumental Variable; UKB, UK Biobank

a GIVs calculated from independent GWAS variants (Linkage disequilibrium R^2^<0.001) with p<5x10^-8^.

**Table 2:** Vitamin D Receptor binding variants (VDR-BVs) instrumental variable (IV) associated with multiple sclerosis susceptibility at p<0.05.

GIV_VDR_^a^	chr:bp	GREAT annotation^b^	OR^c^	95% CI	*P*	*P*_FDR_^c^
rs2881514_A	chr3:16553679	RFTN1 (+1533); OXNAD1 (+246974)	1.10	1.05-1.15	9.4E-05	0.011
rs2531804_A	chr6:28411302	ZSCAN23 (-59)	0.82	0.73-0.92	6.4E-04	0.036
rs12048389_T	chr1:107538722	PRMT6 (-60578)	0.92	0.87-0.99	1.8E-02	0.660
rs55792977_T	chr2:65650863	SPRED2 (+8447); ACTR2 (+195893)	1.05	1.00-1.09	3.2E-02	0.660
rs7309003_C	chr12:97751789	NEDD1 (+450546)	0.95	0.91-1.00	3.2E-02	0.660
rs10995246_C	chr10:64391845	ADO (-172670); ZNF365 (+257895)	1.06	1.00-1.13	3.5E-02	0.660
rs10232857_C	chr7:55601335	VOPP1 (+38882); LANCL2 (+168195)	0.93	0.87-1.00	4.9E-02	0.780

Abbreviations: bp, base pair; chr; chromosome; CI, Confidence Interval; FDR, false discovery rate; GIV, Genetic Instrumental Variable; GREAT, Genomic Regions Enrichment of Annotations Tool; OR, Odds Ratio; VDR-BV, Vitamin D Receptor Binding Variant.

a VDR-BV using as instrumental variable for allelic specific binding. Allele included is the allele associated with increased binding.

b Distance (bp) from VDR-BV to TSS of the nearest upstream and downstream gene from rGREAT.

c Odds ratios are from random effect meta-analyses combining estimates from the four studies. ORs for association between VDR-BV and MS.

## Data Availability

Metadata for KPNC (https://doi.org/10.6084/m9.figshare.22145966.v1) as well as GSA and OMNI participants (https://doi.org/10.6084/m9.figshare.22047314.v1) is available. Complete KPNC data can be accessed by contacting Lisa Barcellos (lbarcellos@berkeley.edu) and Lynn Hollyer (lhollyer@berkeley.edu). Requests will be reviewed by the IRB, and data can be shared upon approval. Complete GSA and OMNI data can be accessed by contacting Ingrid Kockum (Ingrid.Kockum@ki.se). Genotype and phenotype data for UKB participants can be accessed from UKB (https://www.ukbiobank.ac.uk/enable-your-research/about-our-data/genetic-data). GERA data are available on dbGaP (phs000674.v2.p2). R scripts for regression analyses and meta-analyses are available here: https://doi.org/10.5281/zenodo.7647412. Summary statistics from Gallone et al. for VDR-BVs used in this study (SI Appendix, Table S5) and study-specific and meta-analysis associations between GIV_VDR-BV_ and MS susceptibility for all VDR-BVs are available in the supplement (SI Appendix, Tables S6-S9).

## Significance Statement

There is evidence for a causal association between 25-hydroxyvitamin D (25(OH)D) serum levels and multiple sclerosis (MS), but the underlying mechanisms are unknown. We conducted a Mendelian Randomization study to estimate causal associations between vitamin D receptor (VDR) binding at a locus and MS susceptibility using genetic instrumental variables constructed from variants previously associated with allele-specific VDR-binding (VDR-binding variant; VDR-BV) and serum 25(OH)D. Two VDR-BV (rs2881514; rs2531804) were independently associated with MS and rs2881514 demonstrated significant interaction with 25(OH)D, providing evidence for a causal association between rs2881514 and MS. Our results demonstrate that genetic variation in individual VDR binding sites contributes to MS and are relevant to other autoimmune diseases for which a role for vitamin D has been implicated.
