# Supplementary material for: Evidence supports a causal association between allele-specific vitamin D receptor binding and multiple sclerosis among Europeans
Source: Proc Natl Acad Sci U S A. Author manuscript; Available in PMC 2024 Mar 5. (PMC10895341; doi:10.1073/pnas.2302259121)
Supplement: Appendix 1 [file EMS194105-supplement-Appendix_1.pdf]

PNAS

Supporting Information for

Evidence supports causal association between allele-specific vitamin D receptor binding and multiple sclerosis among Europeans

Cameron Adams[a,1]; Ali Manouchehrinia[b,g,1]; Hong L. Quach[a]; Diana L. Quach[a]; Tomas Olsson[b,f,g]; Ingrid Kockum[b,f,g]; Catherine Schaefer[e]; Chris P. Ponting[d]; Lars Alfredsson[b,c,h]; Lisa F. Barcellos[a,e,2]

[a] Genetic Epidemiology and Genomics Laboratory, School of Public Health, University of California, Berkeley, CA, USA
[b] Department of Clinical Neuroscience, Karolinska Institutet, Stockholm, Sweden
[c] Centre for Occupational and Environmental Medicine, Region Stockholm, Stockholm, Sweden
[d] MRC Human Genetics Unit, The Institute of Genetics and Cancer, University of Edinburgh, Western General Hospital, Crewe Road, Edinburgh, UK
[e] Kaiser Permanente Division of Research, Kaiser Permanente Northern California, Oakland, California, USA
[f] Academic Specialist Center, Stockholm, Sweden
[g] Centrum for molecular medicine, Karolinska University hospital, Stockholm, Sweden
[h] Institute of Environmental Medicine, Karolinska Institutet, Stockholm, Sweden.

[1] Co-first authors contributed equally

[2] Lisa Barcellos
324 Stanley Hall, UC Berkeley
Berkeley, CA 94720
Telephone: (510) 642-7814
Email: lbarcellos@berkeley.edu

This PDF file includes:

Figures S1 to S2
Tables S1 to S9

Other supporting materials for this manuscript include the following:

None

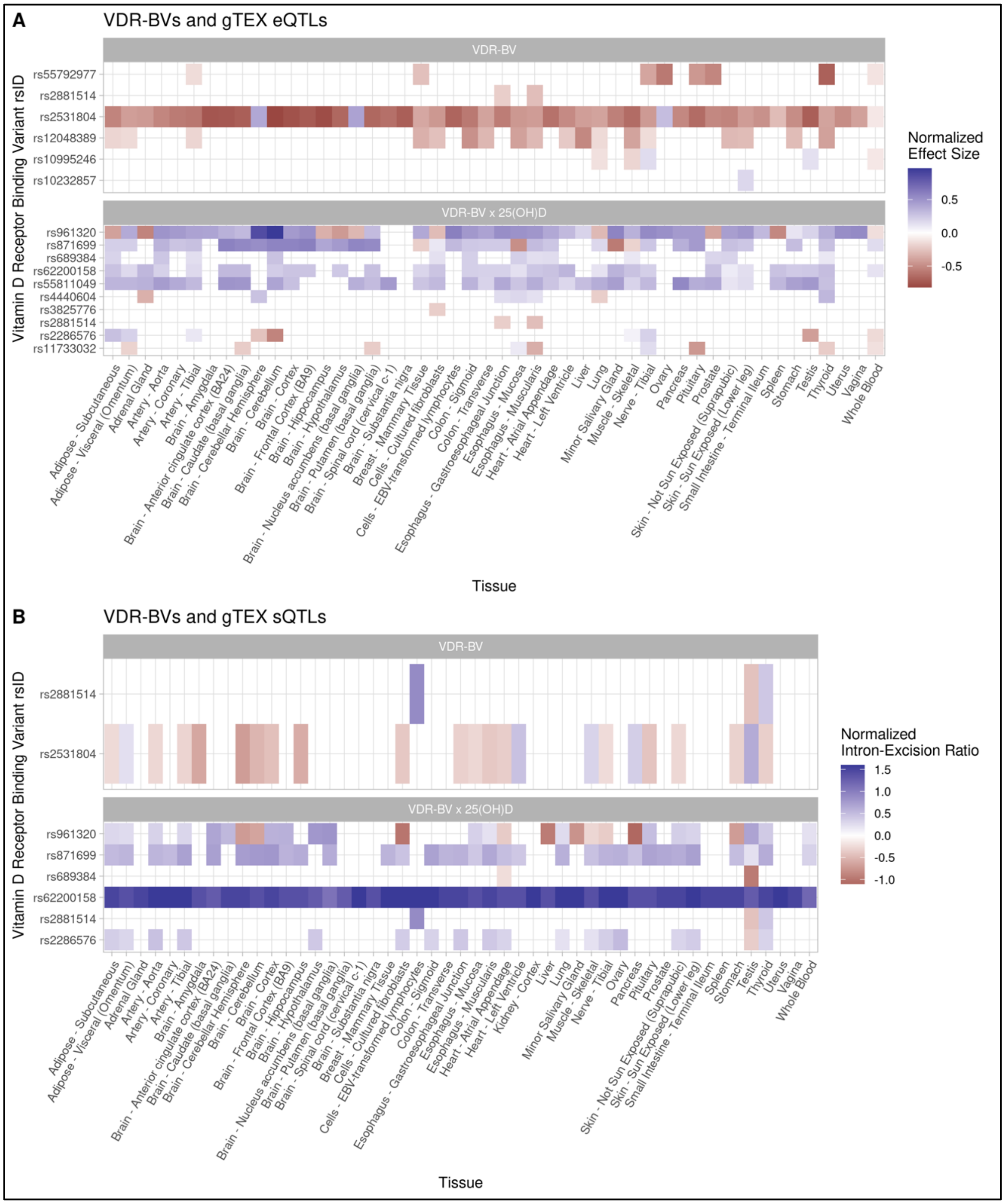

Fig. S1. Evidence of SNP associated tissue specific expression and alternative splicing among vitamin D receptor binding genetic instrumental variables ($GIVs_{VDR}$) presented in Tables 2 and S-1 from GTEx v8 (https://gtexportal.org/). A) expression quantitative trait loci (eQTL) and B) splicing quantitative trait loci (sQTL).

Tissue	Samples	NES	p-value	m-value
Esophagus - Muscularis	465	0.279	1.4e-12	1.00
Esophagus - Gastroesophageal Junction	330	0.210	3.6e-7	1.00
Brain - Substantia nigra	114	0.188	0.04	0.427
Artery - Coronary	213	0.176	1.7e-3	0.903
Liver	208	0.123	0.1	0.103
Cells - EBV-transformed lymphocytes	147	0.118	0.2	0.0700
Kidney - Cortex	73	0.111	0.3	0.111
Testis	322	0.109	0.008	0.250
Uterus	129	0.108	0.2	0.0530
Adrenal Gland	233	0.0998	0.04	0.0390
Prostate	221	0.0819	0.04	0.0130
Brain - Anterior cingulate cortex (BA24)	147	0.0742	0.4	0.110
Stomach	324	0.0713	0.04	0.0690
Spleen	227	0.0694	0.2	0.00500
Brain - Hypothalamus	170	0.0587	0.4	0.0340
Adipose - Subcutaneous	581	0.0581	0.08	0.00
Colon - Sigmoid	318	0.0536	0.1	0.00
Lung	515	0.0502	0.09	0.00
Esophagus - Mucosa	497	0.0495	0.04	0.00
Brain - Cortex	205	0.0472	0.2	0.00
Brain - Amygdala	129	0.0428	0.7	0.0300
Vagina	141	0.0303	0.6	0.00800
Nerve - Tibial	532	0.0294	0.4	0.00
Small Intestine - Terminal Ileum	174	0.0280	0.5	0.00
Pancreas	305	0.0279	0.6	0.00
Adipose - Visceral (Omentum)	469	0.0272	0.5	0.00
Cells - Cultured fibroblasts	483	0.0244	0.3	0.00
Heart - Left Ventricle	386	0.0164	0.6	0.00
Brain - Nucleus accumbens (basal ganglia)	202	0.0154	0.7	0.00
Brain - Frontal Cortex (BA9)	175	0.0111	0.8	0.00
Artery - Tibial	584	0.00593	0.9	0.00
Heart - Atrial Appendage	372	0.00485	0.9	0.00
Whole Blood	670	0.00359	0.8	0.00
Skin - Sun Exposed (Lower leg)	605	0.00201	0.9	0.00
Brain - Cerebellar Hemisphere	175	-0.000905	1	0.00100
Colon - Transverse	368	-0.00443	0.9	0.00
Breast - Mammary Tissue	396	-0.00740	0.8	0.00
Skin - Not Sun Exposed (Suprapubic)	517	-0.0107	0.6	0.00
Brain - Spinal cord (cervical c-1)	126	-0.0116	0.9	0.00
Muscle - Skeletal	706	-0.0186	0.5	0.00
Brain - Putamen (basal ganglia)	170	-0.0252	0.7	0.00
Minor Salivary Gland	144	-0.0262	0.7	0.00
Thyroid	574	-0.0412	0.2	0.00
Ovary	167	-0.0417	0.5	0.00
Artery - Aorta	387	-0.0498	0.1	0.00
Brain - Hippocampus	165	-0.0511	0.5	0.00
Brain - Caudate (basal ganglia)	194	-0.0630	0.3	0.00
Pituitary	237	-0.0793	0.06	0.00
Brain - Cerebellum	209	-0.0839	0.2	0.00

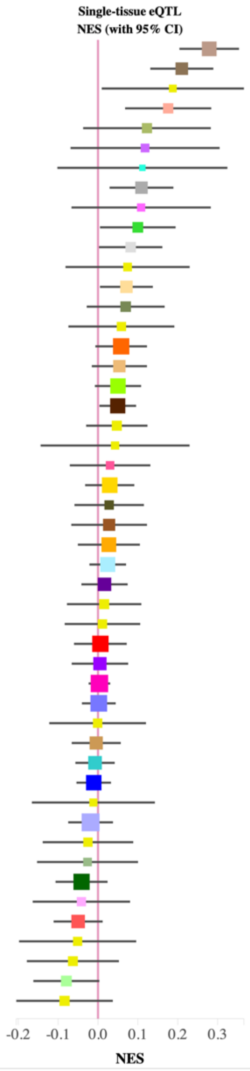

Fig. S-2. Multi-tissue eQTL Comparison for rs2881514_A. The effect allele for normalized expression is G (decreased VDR binding).

Table S1. Vitamin D Receptor binding variants (VDR-BVs) with evidence of interaction with 25(OH)D Genetic Instrumental Variables at p<0.05.

GIV_{VDR}[a]	$GIV_{25(OH)D}$[b]	chr:bp	GREAT annotation[b]	OR[c]	95% CI	*P*	P_{FDR}[c]
rs11733032_G	Jiang et al.	chr4:79560226	BMP2K (-137305); ANXA3 (+87554)	2.91	(1.50-5.66)	0.002	0.24
rs2286576_T	Jiang et al.	chr12:4714007	DYRK4 (+14764); AKAP3 (+44205)	2.47	(1.31-4.65)	0.005	0.45
rs961320_C	Jiang et al.	chr12:49297863	CCDC65 (-68)	3.20	(1.35-7.62)	0.008	0.46
rs55811049_A	Revez et al.	chr5:118324297	DTWD2 (-58)	1.53	(1.11-2.12)	0.009	0.46
rs11729497_G	Revez et al.	chr4:126551382	FAT4 (+313829)	0.72	(0.56-0.93)	0.01	0.46
rs62200158_C	Jiang et al.	chr20:61557905	TCFL5 (-64791); DIDO1 (+11368)	0.22	(0.07-0.72)	0.011	0.47
rs55811049_A	Jiang et al.	chr5:118324297	DTWD2 (-58)	2.76	(1.15-6.64)	0.024	0.53
rs4440604_G	Jiang et al.	chr8:105290834	DCSTAMP (-61219); RIMS2 (+777720)	0.50	(0.28-0.92)	0.024	0.53
rs2881514_A	Jiang et al.	chr3:16553679	RFTN1 (+1533); OXNAD1 (+246974)	2.17	(1.10-4.29)	0.025	0.53
rs12144635_T	Jiang et al.	chr1:173246261	PRDX6 (-200143); TNFSF4 (-69810)	0.26	(0.08-0.85)	0.026	0.53
rs11733032_G	Revez et al.	chr4:79560226	BMP2K (-137305); ANXA3 (+87554)	1.32	(1.03-1.69)	0.026	0.53
rs3825776_T	Jiang et al.	chr15:58746829	LIPC (+44062); ADAM10 (+295347)	0.38	(0.16-0.91)	0.030	0.56
rs871699_T	Jiang et al.	chr11:18127678	SAAL1 (-41)	0.31	(0.10-0.94)	0.039	0.60
rs689384_C	Jiang et al.	chr18:54318453	WDR7 (-162)	2.62	(1.02-6.75)	0.047	0.63

Abbreviations: bp, base pair; chr; chromosome; CI, Confidence Interval; FDR, false discovery rate; GIV, genetic instrumental variable; GREAT, Genomic Regions Enrichment of Annotations Tool; OR, Odds Ratio; VDR-BV, Vitamin D Receptor Binding Variant

[a] VDR-BV using as instrumental variable for allelic specific binding. Allele including is the allele associated with decreased binding affinity.

[b] GIV calculated using summary statistics from Jiang et al 2019 and Revez et al 2020 GWAS.

[c] Distance from VDR-BV to TSS of nearest upstream and downstream gene from rGREAT.

[d] Odds ratios are from random effect meta-analyses combining estimates from the four studies. ORs are the multiplicative interaction coefficient for interaction between 25(OH)D GIV and VDR-BV.

Table S2. Results from GREAT enrichment analyses of VDR-BVs associated with MS susceptibility.

**Ontology**	**ID**	**Description**	**GREAT[a] Enrichment *P*-value**	**VDR-BV**
BPs	GO:0002443	leukocyte mediated immunity	0.0044	rs12144635; rs2881514; rs11733032; rs3825776
BP	GO:0002252	immune effector process	0.0074	rs12144635; rs2881514; rs11733032; rs3825776
BP	GO:0002274	myeloid leukocyte activation	0.0074	rs12144635; rs11733032; rs4440604; rs3825776
BP	GO:0006887	exocytosis	0.0074	rs12144635; rs11733032; rs4440604; rs3825776
BP	GO:0032940	secretion by cell	0.0074	rs12144635; rs11733032; rs4440604; rs3825776
BP	GO:0045055	regulated exocytosis	0.0074	rs12144635; rs11733032; rs4440604; rs3825776
BP	GO:0046903	secretion	0.0074	rs12144635; rs11733032; rs4440604; rs3825776
CC	GO:0099503	secretory vesicle	0.0048	rs12144635; rs11733032; rs2286576; rs3825776; rs689384
CC	GO:0031410	cytoplasmic vesicle	0.0060	rs12144635; rs2881514; rs11733032; rs4440604; rs2286576; rs3825776; rs689384
CC	GO:0097708	intracellular vesicle	0.0060	rs12144635; rs2881514; rs11733032; rs4440604; rs2286576; rs3825776; rs689384
CC	GO:0042581	specific granule	0.0110	rs11733032; rs3825776
CC	GO:0030141	secretory granule	0.0120	rs12144635; rs11733032; rs2286576; rs3825776
MF	GO:0004620	phospholipase activity	0.0300	rs12144635; rs3825776

Abbreviations: BP, Biological Process; CC, Cellular Component; MF, Molecular Function; GREAT, Genomic Regions Enrichment of Annotations Tool; VDR-BV, Vitamin D Receptor Binding Variant.
[a] Enrichment analyses performed using rGREAT. Background regions were locations of all 112 VDR-BV used in regression analyses, test regions were the 12 VDR-BVs with nominal associations with MS presented in Tables 2 and S1.

Table S3. Variants used in Jiang et al 25(OH)D GIV.

Variant[a]	effect_size	effect_allele	other_allele
rs17775309	0.0173	G	T
rs2298850	-0.0893	C	G
rs12785878	0.0363	T	G
rs2060793	-0.0296	G	A
rs2597193	0.0187	A	G
rs4762258	0.0158	G	A
rs8018720	-0.0168	C	G
rs17216707	-0.0263	C	T

[a] Variants from Jiang et al. 2018 GWAS on 25(OH)D. Variants, alleles, and effect sizes extracted with TwoSampleMR::extract_instruments("ebi-a-GCST005367" , clump=T, p1 = 5e-08) in R.

Table S4. Variants used in Revez et al 25(OH)D GIV.

Variant[a]	effect_size	effect_allele	other_allele
rs35408430	-0.0213322	T	C
rs2642439	-0.0145212	G	A
rs2131925	-0.0222318	T	G
rs6672758	0.0160992	T	C
rs512083	0.0122018	C	T
rs6671730	-0.015115	A	G
rs7528419	0.0200747	G	A
rs10908469	0.0158902	C	A
rs7522116	-0.0126086	T	C
rs1260326	0.0224633	C	T
rs13011615	0.0169587	T	A
rs6723486	-0.0111173	T	C
rs7569755	0.0132378	A	G
rs1047891	-0.0125534	A	C
rs2012736	-0.0481921	A	C
rs6724965	-0.0165826	G	A
rs58387006	-0.0137174	C	A
rs34186890	-0.0149926	G	A
rs2246832	0.017206	T	A
rs113642272	-0.019613	T	G
rs11458206	0.0135809	TG	T
rs10028494	0.028108	C	A
rs6834488	-0.0146317	T	C
rs4616820	-0.0123353	T	C
rs2579309	0.0228904	A	G
rs36102036	0.0337496	A	G
rs1352846	-0.193371	G	A
rs10084913	0.0186868	G	A
rs4364259	0.0165067	A	G
rs78649910	-0.0193309	A	T
rs3890624	0.0112736	G	A
rs1966478	-0.0123541	C	T
rs2608984	-0.0208623	T	A
rs72834856	-0.0235978	G	T
rs11751024	-0.0125611	A	C
rs9467550	-0.0188955	G	A
rs143069752	0.0224092	A	T

rs9476310	0.0117234	T	C
rs1858889	0.0111372	C	A
rs2346264	-0.0147004	C	A
rs7784802	0.0137867	T	A
rs17144574	-0.01526	C	T
rs41301394	0.0126168	T	C
rs57459725	-0.0176615	G	C
rs804281	0.0157516	G	A
rs34726834	0.0149657	T	C
rs12056768	-0.021949	G	T
rs9409266	-0.0184021	A	G
rs532436	-0.0169916	A	G
rs13284054	0.0185546	C	T
rs4418728	0.0121203	T	G
rs10822145	-0.012578	T	C
rs2297991	0.0120361	C	T
rs3925446	0.0162616	A	G
rs964184	0.0431884	C	G
rs1660818	0.0159911	A	G
rs12798050	0.110611	T	C
rs2276360	0.110966	C	G
rs113140528	0.0327849	T	A
rs7128011	-0.0753553	A	G
rs10766281	0.0154476	G	A
rs575976	0.0155802	G	A
rs33981819	0.0112242	G	T
rs2847500	-0.0221038	A	G
rs12317268	-0.0206326	G	A
rs11182428	-0.0123489	C	T
rs8181687	0.0116958	A	G
rs10859995	-0.0399464	C	T
rs73413596	0.0237483	C	T
rs2171427	-0.0154101	A	G
rs35014562	-0.0126538	GA	G
rs8018720	-0.0383637	C	G
rs2756119	0.0121881	A	G
rs62012775	-0.0162631	T	A
rs1800588	-0.0329963	T	C
rs1532085	0.0260168	G	A

rs2123930	-0.014114	A	G
rs77924615	-0.0144881	A	G
rs11542462	-0.0232523	A	G
rs8063565	0.0123302	C	G
rs11076175	0.023978	G	A
rs34177108	-0.0122879	A	C
rs2952289	0.0178756	T	C
rs2659007	0.0114099	A	G
rs61003750	-0.0120389	C	G
rs12949853	0.0144344	A	G
rs4121823	-0.0185043	A	T
rs8091117	-0.02514	A	C
rs2037511	0.0170377	A	G
rs12462826	-0.0131168	A	G
rs429358	-0.0231982	C	T
rs212100	-0.065701	C	T
rs3814995	-0.0126428	T	C
rs200210321	0.0395561	AG	A
rs142158911	0.0266711	A	G
rs11606	0.0112201	G	C
rs62115743	0.0274464	T	C
rs1841850	0.0279578	C	A
rs8121940	-0.037716	G	C
rs2229742	-0.0251367	C	G
rs2074735	0.027808	C	G
rs115621755	-0.012627	T	C
rs6003465	-0.0119188	C	T

[a] Variants from Revez et al. 2020 GWAS on 25(OH)D. Variants, alleles, and effect sizes collected with TwoSampleMR::extract_instruments("ebi-a-GCST90000615" , clump=T, p1 = 5e-08) in R.

Table S5. Effect sizes and alleles for association between VDR-BVs and VDR occupancy as measured using ChipExo from Gallone et al. 2017.

VDR-BV	chr_hg19	bp_hg19	effect_allele	other_allele	beta	SE
rs10797448	chr1	233348941	C	T	0.83	0.16
rs10800934	chr1	203429502	G	C	0.86	0.14
rs11263852	chr1	36088841	G	C	0.65	0.18
rs11585739	chr1	230110153	C	T	0.43	0.12
rs12021542	chr1	230981596	T	A	0.82	0.17
rs12048389	chr1	106996101	T	C	0.66	0.13
rs12144635	chr1	173277123	T	C	0.47	0.16
rs2331903	chr1	180981733	T	C	0.31	0.07
rs3738504	chr1	42767136	C	G	0.56	0.16
rs4916354	chr1	173415688	C	T	0.58	0.09
rs56077241	chr1	167715577	C	A	0.85	0.15
rs664182	chr1	33127286	A	G	0.71	0.19
rs6687177	chr1	157182009	G	C	0.70	0.16
rs10179357	chr2	205368061	A	C	0.82	0.17
rs12613956	chr2	44244270	T	G	0.82	0.17
rs12624197	chr2	241315322	C	G	0.67	0.14
rs13021990	chr2	13853805	T	C	0.82	0.17
rs2241876	chr2	233278088	G	T	0.82	0.17
rs3813228	chr2	73780218	A	G	0.28	0.10
rs4413108	chr2	126864471	C	T	0.82	0.17
rs4670248	chr2	38262143	T	C	0.86	0.14
rs55792977	chr2	65423730	T	G	0.83	0.16
rs6436813	chr2	228683248	G	C	0.82	0.17
rs6727980	chr2	20868589	T	G	0.91	0.09
rs13086176	chr3	57100218	C	T	0.82	0.17
rs13098781	chr3	18716927	G	A	0.82	0.17
rs13324462	chr3	197713714	A	G	0.76	0.16
rs2279908	chr3	44729636	C	T	0.86	0.14
rs2574711	chr3	11626292	C	T	0.68	0.17
rs2881514	chr3	16512173	A	G	0.75	0.14
rs62248690	chr3	43181246	G	A	0.63	0.10
rs62410372	chr3	179260754	A	G	0.82	0.17
rs9818027	chr3	189489558	G	A	0.76	0.16
rs998909	chr3	52771077	A	G	0.47	0.14
rs11132491	chr4	187913080	G	T	0.82	0.17
rs11544037	chr4	158667824	C	A	0.85	0.15
rs11729497	chr4	125630228	G	C	0.82	0.17
rs11733032	chr4	78639073	G	A	0.69	0.07
rs34035218	chr4	37562171	C	T	0.82	0.17
rs10069743	chr5	129088984	T	C	0.82	0.17
rs1430204	chr5	88382815	C	T	0.82	0.17
rs2170502	chr5	7911030	G	A	0.82	0.17
rs257916	chr5	129085865	T	G	0.82	0.17
rs4276369	chr5	61330048	G	A	0.83	0.16
rs4703608	chr5	73654711	A	G	0.68	0.15
rs55811049	chr5	118988603	A	C	0.85	0.15
rs67061065	chr5	36242110	C	T	0.82	0.17
rs712615	chr5	116885938	T	A	0.73	0.18
rs72775970	chr5	97643953	C	T	0.83	0.16
rs10949389	chr6	16942833	A	G	0.82	0.17
rs12214623	chr6	113065866	T	C	0.83	0.16
rs1782437	chr6	90425069	G	A	0.83	0.12
rs178399	chr6	2791449	G	A	0.95	0.03
rs2531804	chr6	28443526	A	G	0.44	0.15

rs274436	chr6	104326558	C	T	0.83	0.16
rs3798259	chr6	45921171	G	T	0.60	0.15
rs396360	chr6	13260221	C	T	0.85	0.15
rs66773803	chr6	130967385	C	T	0.82	0.17
rs9347904	chr6	158032179	T	C	0.88	0.11
rs9348121	chr6	166341524	C	A	0.75	0.17
rs9493768	chr6	133953159	T	A	0.35	0.08
rs10232857	chr7	55533643	C	T	0.83	0.16
rs10951263	chr7	30163611	G	A	0.82	0.17
rs2686823	chr7	47937388	C	T	0.82	0.17
rs2708881	chr7	47937496	C	A	0.35	0.13
rs34571901	chr7	37038985	A	G	0.83	0.16
rs698631	chr7	56195405	C	T	0.86	0.14
rs1347322	chr8	102536334	G	A	0.47	0.16
rs1389206	chr8	71012627	G	A	0.82	0.17
rs4440604	chr8	104278607	G	A	0.86	0.14
rs10119345	chr9	110898705	C	T	0.82	0.17
rs10812343	chr9	2616875	G	A	0.46	0.15
rs2297876	chr9	35604048	G	A	0.71	0.19
rs72761985	chr9	113517421	G	T	0.60	0.16
rs10883801	chr10	102918130	C	A	0.82	0.17
rs10904183	chr10	4097576	A	G	0.73	0.18
rs10995246	chr10	62632086	C	A	0.82	0.17
rs12098858	chr10	67012714	T	C	0.73	0.18
rs12763743	chr10	95832399	A	T	0.83	0.16
rs1750737	chr10	11113369	A	G	0.82	0.17
rs2387397	chr10	6348230	C	G	0.82	0.17
rs3750619	chr10	110919278	G	A	0.40	0.10
rs3793786	chr10	49535042	A	G	0.83	0.16
rs670446	chr11	107602007	C	T	0.82	0.17
rs871699	chr11	18106132	T	C	0.57	0.14
rs10862993	chr12	85531560	T	C	0.83	0.16
rs12300068	chr12	79087591	G	A	0.82	0.17
rs2286576	chr12	4604842	T	G	0.86	0.14
rs2638263	chr12	40152998	A	T	0.83	0.16
rs7307718	chr12	40632248	C	T	0.85	0.15
rs7309003	chr12	97358012	C	G	0.82	0.17
rs961320	chr12	48904081	C	T	0.75	0.05
rs1923909	chr13	24124980	T	C	0.83	0.16
rs45465203	chr13	48038078	G	A	0.60	0.18
rs12594789	chr15	65304862	G	A	0.82	0.17
rs3825776	chr15	58454631	T	C	0.65	0.18
rs4408496	chr15	70463694	T	C	0.50	0.16
rs58583822	chr15	59165936	G	A	0.82	0.17
rs8035605	chr15	35756386	G	T	0.82	0.17
rs2059292	chr16	62423133	A	T	0.83	0.16
rs35787586	chr16	19067848	C	A	0.70	0.16
rs11657238	chr17	48908272	G	A	0.57	0.17
rs1380180	chr17	15289882	A	C	0.82	0.17
rs34038545	chr17	47067541	G	C	0.91	0.09
rs4796758	chr17	42017501	T	G	0.67	0.12
rs1039143	chr18	32714710	A	G	0.83	0.16
rs689384	chr18	56651223	C	T	0.71	0.19
rs1073378	chr20	13247424	C	T	0.90	0.04
rs16993008	chr20	47861743	G	C	0.73	0.18
rs62200158	chr20	62926554	C	T	0.89	0.10
rs74364610	chr21	42557163	G	A	0.83	0.16
rs2267393	chr22	38705614	G	C	0.83	0.16

Abbreviations: KPNC, Kaiser Permanente Northern California; UKB, UK Biobank; FDR, false discovery rate; GIV, genetic instrumental variable; VDR-BV, vitamin D receptor binding variant

Table S6. Associations between vitamin D receptor binding variants (VDR-BV) and multiple sclerosis within each study.

					KPNC				GSA				Human OMNI				UKB			
VDR-BV	chr hg19	bp hg19	effect allele	other allele	OR	95% CI	SE	p.value	OR	95% CI	SE	p.value	OR	95% CI	SE	p.value	OR	95% CI	SE	p.value
rs10797448_C	chr1	233348941	C	T	1.07	(0.96-1.19)	###	2.42E-01	0.97	(0.79-1.18)	0.10	7.41E-01	1.09	(0.98-1.21)	0.05	1.03E-01	0.94	(0.87-1.02)	0.04	1.43E-01
rs10800934_G	chr1	203429502	G	C	0.99	(0.89-1.1)	###	8.29E-01	0.98	(0.86-1.12)	0.06	7.77E-01	1.01	(0.94-1.07)	0.03	8.35E-01	1.05	(0.98-1.14)	0.04	1.76E-01
rs11263852_G	chr1	36088841	G	C	0.74	(0.57-0.95)	###	1.94E-02	1.00	(0.84-1.21)	0.09	9.80E-01	0.98	(0.89-1.08)	0.05	7.35E-01	0.87	(0.72-1.05)	0.10	1.61E-01
rs11585739_C	chr1	230110153	C	T	1.06	(0.85-1.31)	###	6.08E-01	1.01	(0.89-1.14)	0.06	9.37E-01	0.97	(0.91-1.04)	0.03	4.30E-01	0.98	(0.84-1.14)	0.08	8.24E-01
rs12021542_T	chr1	230981596	T	A	1.01	(0.89-1.15)	###	8.53E-01	0.96	(0.83-1.12)	0.07	6.18E-01	0.95	(0.88-1.02)	0.04	1.55E-01	1.04	(0.94-1.14)	0.05	4.81E-01
rs12048389_T	chr1	106996101	T	C	0.88	(0.74-1.05)	###	1.65E-01	0.89	(0.74-1.07)	0.09	2.32E-01	0.96	(0.88-1.06)	0.05	4.59E-01	0.89	(0.79-1.01)	0.06	7.78E-02
rs12144635_T	chr1	173277123	T	C	1.37	(0.99-1.91)	###	6.01E-02	1.20	(0.99-1.45)	0.10	6.65E-02	0.98	(0.89-1.09)	0.05	7.59E-01	1.14	(0.91-1.43)	0.11	2.61E-01
rs2331903_T	chr1	180981733	T	C	1.02	(0.76-1.38)	###	8.97E-01	0.85	(0.75-0.96)	0.06	9.84E-03	0.99	(0.93-1.05)	0.03	6.85E-01	1.10	(0.89-1.36)	0.11	3.76E-01
rs3738504_C	chr1	42767136	C	G	0.98	(0.83-1.15)	###	7.75E-01	1.03	(0.91-1.16)	0.06	6.51E-01	1.03	(0.96-1.09)	0.03	4.00E-01	1.01	(0.9-1.13)	0.06	8.93E-01
rs4916354_C	chr1	173415688	C	T	0.91	(0.75-1.12)	###	3.78E-01	1.14	(0.99-1.31)	0.07	7.53E-02	0.92	(0.85-0.99)	0.04	2.58E-02	1.01	(0.88-1.16)	0.07	9.09E-01
rs56077241_C	chr1	167715577	C	A	1.06	(0.86-1.3)	###	5.62E-01	0.94	(0.61-1.45)	0.22	7.68E-01	0.94	(0.75-1.17)	0.11	5.67E-01	1.07	(0.91-1.24)	0.08	4.09E-01
rs664182_A	chr1	33127286	A	G	1.08	(0.93-1.25)	###	3.24E-01	1.12	(0.97-1.29)	0.07	1.12E-01	1.03	(0.96-1.1)	0.04	4.70E-01	0.98	(0.88-1.08)	0.05	6.46E-01
rs6687177_G	chr1	157182009	G	C	1.01	(0.89-1.15)	###	8.77E-01	1.08	(0.95-1.22)	0.06	2.29E-01	0.95	(0.89-1.01)	0.03	1.24E-01	1.00	(0.91-1.09)	0.05	9.19E-01
rs10179357_A	chr2	205368061	A	C	0.98	(0.83-1.15)	###	8.25E-01	0.96	(0.81-1.14)	0.09	6.44E-01	1.05	(0.95-1.15)	0.05	3.18E-01	1.01	(0.9-1.13)	0.06	8.98E-01
rs12613956_T	chr2	44244270	T	G	0.90	(0.78-1.05)	###	1.76E-01	1.03	(0.89-1.2)	0.08	6.91E-01	1.03	(0.95-1.11)	0.04	4.64E-01	1.05	(0.94-1.17)	0.06	3.90E-01
rs12624197_C	chr2	241315322	C	G	0.98	(0.86-1.12)	###	7.87E-01	0.94	(0.83-1.06)	0.06	3.01E-01	0.99	(0.93-1.05)	0.03	6.88E-01	1.02	(0.93-1.13)	0.05	6.29E-01
rs13021990_T	chr2	13853805	T	C	1.00	(0.85-1.18)	###	9.74E-01	1.06	(0.89-1.26)	0.09	5.04E-01	1.03	(0.94-1.13)	0.05	5.27E-01	1.06	(0.94-1.2)	0.06	3.54E-01
rs2241876_G	chr2	233278088	G	T	0.98	(0.87-1.11)	###	7.49E-01	0.92	(0.8-1.05)	0.07	2.03E-01	1.00	(0.94-1.08)	0.04	9.23E-01	1.02	(0.93-1.11)	0.05	6.90E-01
rs3813228_A	chr2	73780218	A	G	0.91	(0.65-1.29)	###	6.10E-01	1.00	(0.87-1.14)	0.07	9.56E-01	1.03	(0.96-1.1)	0.03	4.11E-01	0.97	(0.76-1.24)	0.12	8.22E-01
rs4413108_C	chr2	126864471	C	T	1.19	(0.99-1.44)	###	6.34E-02	0.87	(0.69-1.09)	0.11	2.23E-01	1.02	(0.91-1.14)	0.06	7.70E-01	1.03	(0.91-1.18)	0.07	6.57E-01
rs4670248_T	chr2	38262143	T	C	0.97	(0.84-1.12)	###	6.39E-01	1.03	(0.87-1.21)	0.08	7.27E-01	1.02	(0.94-1.12)	0.04	6.01E-01	0.95	(0.85-1.05)	0.05	2.89E-01
rs55792977_T	chr2	65423730	T	G	1.05	(0.94-1.18)	###	3.47E-01	1.06	(0.94-1.2)	0.06	3.45E-01	1.06	(1-1.14)	0.03	6.38E-02	1.02	(0.94-1.1)	0.04	6.61E-01
rs6436813_G	chr2	228683248	G	C	1.03	(0.91-1.17)	###	6.20E-01	0.96	(0.83-1.12)	0.08	6.35E-01	1.02	(0.94-1.1)	0.04	6.79E-01	1.00	(0.92-1.09)	0.04	9.95E-01
rs6727980_T	chr2	20868589	T	G	0.82	(0.69-0.99)	###	3.41E-02	0.95	(0.74-1.2)	0.12	6.46E-01	0.96	(0.85-1.09)	0.06	5.68E-01	0.95	(0.83-1.1)	0.07	4.89E-01
rs13086176_C	chr3	57100218	C	T	0.94	(0.84-1.06)	###	3.45E-01	1.00	(0.88-1.14)	0.07	9.95E-01	1.07	(1-1.15)	0.04	5.61E-02	0.95	(0.87-1.03)	0.04	2.11E-01
rs13098781_G	chr3	18716927	G	A	0.90	(0.78-1.05)	###	1.83E-01	1.08	(0.89-1.3)	0.10	4.35E-01	1.07	(0.97-1.18)	0.05	1.98E-01	1.07	(0.96-1.18)	0.05	2.08E-01
rs13324462_A	chr3	197713714	A	G	0.92	(0.75-1.13)	###	4.16E-01	1.22	(0.99-1.5)	0.11	6.08E-02	0.95	(0.85-1.07)	0.06	3.90E-01	0.99	(0.86-1.15)	0.07	9.03E-01
rs2279908_C	chr3	44729636	C	T	1.01	(0.9-1.12)	###	9.01E-01	1.07	(0.95-1.21)	0.06	2.68E-01	1.01	(0.95-1.08)	0.03	7.64E-01	1.01	(0.94-1.09)	0.04	7.58E-01
rs2574711_C	chr3	11626292	C	T	0.92	(0.72-1.18)	###	5.07E-01	0.86	(0.68-1.07)	0.12	1.81E-01	1.08	(0.96-1.21)	0.06	1.81E-01	1.20	(0.99-1.46)	0.10	6.72E-02
rs2881514_A	chr3	16512173	A	G	1.11	(0.99-1.26)	###	8.59E-02	1.10	(0.96-1.26)	0.07	1.87E-01	1.11	(1.03-1.19)	0.04	5.91E-03	1.08	(0.99-1.18)	0.04	7.40E-02
rs62248690_G	chr3	43181246	G	A	1.00	(0.78-1.3)	###	9.79E-01	0.87	(0.55-1.36)	0.23	5.54E-01	0.82	(0.66-1.03)	0.11	8.56E-02	0.98	(0.83-1.17)	0.09	8.18E-01
rs62410372_A	chr3	179260754	A	G	1.05	(0.94-1.17)	###	4.29E-01	1.02	(0.88-1.19)	0.08	7.52E-01	0.97	(0.9-1.05)	0.04	4.58E-01	1.04	(0.96-1.13)	0.04	3.20E-01
rs9818027_G	chr3	189489558	G	A	0.95	(0.82-1.1)	###	5.10E-01	0.99	(0.85-1.16)	0.08	9.09E-01	0.97	(0.89-1.05)	0.04	4.30E-01	1.03	(0.92-1.15)	0.06	6.03E-01
rs998909_A	chr3	52771077	A	G	0.94	(0.77-1.14)	###	5.21E-01	1.06	(0.86-1.3)	0.11	6.06E-01	1.01	(0.91-1.13)	0.06	8.43E-01	1.06	(0.92-1.22)	0.07	3.99E-01
rs11132491_G	chr4	187913080	G	T	1.05	(0.93-1.2)	###	4.09E-01	1.27	(0.98-1.65)	0.13	7.80E-02	0.91	(0.79-1.04)	0.07	1.52E-01	1.01	(0.92-1.11)	0.05	8.59E-01
rs11544037_C	chr4	158667824	C	A	0.89	(0.77-1.04)	###	1.40E-01	1.33	(1.11-1.58)	0.09	1.53E-03	1.06	(0.97-1.17)	0.05	2.15E-01	1.04	(0.93-1.16)	0.06	4.68E-01
rs11729497_G	chr4	125630228	G	C	0.90	(0.8-1)	###	5.58E-02	1.13	(0.97-1.3)	0.07	1.07E-01	1.05	(0.97-1.14)	0.04	2.15E-01	1.11	(1.03-1.2)	0.04	9.73E-03
rs11733032_G	chr4	78639073	G	A	1.00	(0.86-1.16)	###	9.90E-01	1.04	(0.93-1.17)	0.06	4.80E-01	0.95	(0.9-1.02)	0.03	1.48E-01	0.97	(0.87-1.08)	0.05	5.98E-01
rs34035218_C	chr4	37562171	C	T	0.97	(0.87-1.08)	###	5.97E-01	1.03	(0.91-1.16)	0.06	6.61E-01	1.03	(0.96-1.09)	0.03	4.01E-01	1.06	(0.98-1.15)	0.04	1.35E-01
rs10069743_T	chr5	129088984	T	C	1.12	(0.91-1.36)	###	2.69E-01	1.08	(0.86-1.37)	0.12	5.08E-01	1.15	(1.02-1.31)	0.06	2.57E-02	0.98	(0.85-1.12)	0.07	7.90E-01
rs1430204_C	chr5	88382815	C	T	1.04	(0.91-1.18)	###	5.76E-01	1.49	(1.15-1.95)	0.13	3.03E-03	0.96	(0.84-1.09)	0.07	5.14E-01	0.97	(0.88-1.07)	0.05	5.44E-01
rs2170502_G	chr5	7911030	G	A	0.94	(0.84-1.05)	###	2.77E-01	1.09	(0.96-1.23)	0.06	1.91E-01	1.00	(0.94-1.07)	0.03	9.90E-01	0.97	(0.89-1.05)	0.04	4.29E-01
rs257916_T	chr5	129085865	T	G	1.05	(0.93-1.17)	###	4.29E-01	1.09	(0.96-1.24)	0.06	1.62E-01	1.03	(0.96-1.1)	0.03	4.25E-01	0.98	(0.9-1.06)	0.04	5.81E-01
rs4276369_G	chr5	61330048	G	A	1.20	(1.04-1.37)	###	1.13E-02	0.95	(0.82-1.11)	0.08	5.02E-01	0.99	(0.92-1.08)	0.04	8.49E-01	1.02	(0.92-1.13)	0.05	7.29E-01
rs4703608_A	chr5	73654711	A	G	1.01	(0.87-1.17)	###	8.89E-01	0.93	(0.78-1.09)	0.08	3.60E-01	1.08	(0.99-1.18)	0.04	1.02E-01	0.99	(0.89-1.1)	0.05	8.38E-01
rs55811049_A	chr5	118988603	A	C	1.13	(0.96-1.32)	###	1.46E-01	1.01	(0.82-1.23)	0.10	9.45E-01	1.06	(0.97-1.16)	0.04	1.97E-01	1.03	(0.92-1.16)	0.06	6.12E-01
rs67061065_C	chr5	36242110	C	T	0.97	(0.87-1.09)	###	6.52E-01	1.06	(0.94-1.2)	0.06	3.40E-01	0.96	(0.9-1.03)	0.03	2.65E-01	0.95	(0.88-1.03)	0.04	2.49E-01
rs712615_T	chr5	116885938	T	A	0.96	(0.84-1.09)	###	5.13E-01	0.98	(0.88-1.1)	0.06	7.43E-01	1.01	(0.95-1.07)	0.03	7.67E-01	0.94	(0.86-1.03)	0.05	1.87E-01
rs72775970_C	chr5	97643953	C	T	1.03	(0.87-1.22)	###	7.59E-01	0.97	(0.8-1.18)	0.10	7.66E-01	0.97	(0.88-1.08)	0.05	6.22E-01	1.00	(0.89-1.12)	0.06	9.88E-01
rs10949389_A	chr6	16942833	A	G	1.03	(0.92-1.17)	###	5.74E-01	0.99	(0.88-1.12)	0.06	8.97E-01	0.98	(0.92-1.04)	0.03	4.94E-01	0.94	(0.87-1.03)	0.04	1.70E-01
rs12214623_T	chr6	113065866	T	C	0.93	(0.82-1.07)	###	3.13E-01	1.07	(0.92-1.24)	0.08	3.98E-01	0.93	(0.86-1)	0.04	5.60E-02	1.10	(1-1.21)	0.05	4.60E-02
rs1782437_G	chr6	90425069	G	A	1.06	(0.91-1.25)	###	4.58E-01	0.92	(0.62-1.36)	0.20	6.85E-01	1.01	(0.82-1.23)	0.10	9.34E-01	0.97	(0.87-1.09)	0.06	6.33E-01
rs178399_G	chr6	2791449	G	A	0.86	(0.78-0.94)	###	1.67E-03	0.86	(0.77-0.98)	0.06	1.80E-02	0.98	(0.92-1.04)	0.03	4.98E-01	1.01	(0.95-1.08)	0.03	7.16E-01
rs2531804_A	chr6	28443526	A	G	0.80	(0.65-0.98)	###	3.16E-02	0.91	(0.8-1.03)	0.06	1.27E-01	0.87	(0.81-0.93)	0.03	2.52E-05	0.69	(0.59-0.79)	0.07	4.20E-07
rs274436_C	chr6	104326558	C	T	0.93	(0.83-1.05)	###	2.49E-01	1.00	(0.88-1.13)	0.06	9.96E-01	1.05	(0.98-1.12)	0.03	1.68E-01	1.06	(0.98-1.16)	0.04	1.48E-01
rs3798259_G	chr6	45921171	G	T	0.80	(0.68-0.93)	###	4.64E-03	1.00	(0.88-1.14)	0.06	9.51E-01	0.93	(0.87-1)	0.03	4.86E-02	1.04	(0.93-1.17)	0.06	5.05E-01
rs396360_C	chr6	13260221	C	T	1.00	(0.84-1.21)	###	9.61E-01	0.88	(0.71-1.08)	0.11	2.26E-01	1.03	(0.92-1.15)	0.06	5.89E-01	1.00	(0.88-1.15)	0.07	9.51E-01
rs66773803_C	chr6	130967385	C	T	0.98	(0.83-1.18)	###	8.66E-01	0.99	(0.79-1.23)	0.11	9.15E-01	1.02	(0.91-1.15)	0.06	7.18E-01	0.93	(0.82-1.05)	0.07	2.45E-01
rs9347904_T	chr6	158032179	T	C	0.93	(0.83-1.03)	###	1.82E-01	0.78	(0.66-0.91)	0.08	2.05E-03	0.98	(0.9-1.06)	0.04	5.62E-01	0.99	(0.92-1.07)	0.04	8.38E-01
rs9348121_C	chr6	166341524	C	A	1.03	(0.91-1.16)	###	6.92E-01	1.05	(0.92-1.2)	0.07	4.56E-01	0.96	(0.9-1.03)	0.04	2.48E-01	1.00	(0.92-1.09)	0.04	9.37E-01
rs9493768_T	chr6	133953159	T	A	1.04	(0.66-1.67)	###	8.58E-01	1.02	(0.81-1.29)	0.12	8.55E-01	1.01	(0.9-1.15)	0.06	8.18E-01	0.90	(0.64-1.27)	0.17	5.51E-01
rs10232857_C	chr7	55533643	C	T	1.04	(0.87-1.24)	###	6.48E-01	0.94	(0.78-1.13)	0.10	5.16E-01	0.91	(0.83-1)	0.05	5.47E-02	0.92	(0.8-1.06)	0.07	2.51E-01
rs10951263_G	chr7	30163611	G	A	1.02	(0.9-1.16)	###	7.49E-01	0.85	(0.69-1.03)	0.10	1.01E-01	1.05	(0.95-1.17)	0.05	3.17E-01	0.97	(0.89-1.07)	0.05	5.70E-01
rs2686823_C	chr7	47937388	C	T	0.91	(0.81-1.01)	###	8.13E-02	0.94	(0.84-1.05)	0.06	2.81E-01	1.02	(0.96-1.08)	0.03	4.68E-01	1.03	(0.95-1.11)	0.04	4.98E-01
rs2708881_C	chr7	47937496	C	A	1.26	(0.97-1.64)	###	8.08E-02	1.08	(0.94-1.24)	0.07	2.81E-01	0.97	(0.91-1.05)	0.04	4.68E-01	0.94	(0.78-1.13)	0.09	4.99E-01
rs34571901_A	chr7	37038985	A	G	0.91	(0.76-1.11)	###	3.45E-01	1.08	(0.87-1.34)	0.11	4.88E-01	0.95	(0.85-1.06)	0.06	3.26E-01	1.13	(0.98-1.31)	0.07	9.14E-02
rs698631_C	chr7	56195405	C	T	0.90	(0.78-1.05)	###	1.81E-01	0.96	(0.76-1.21)	0.12	7.30E-01	1.02	(0.9-1.16)	0.06	7.13E-01	0.99	(0.89-1.11)	0.06	8.69E-01
rs1347322_G	chr8	102536334	G	A	1.08	(0.85-1.37)	###	5.56E-01	0.92	(0.8-1.06)	0.07	2.50E-01	0.97	(0.91-1.05)	0.04	4.54E-01	0.90	(0.76-1.06)	0.09	2.09E-01
rs1389206_G	chr8	71012627	G	A	1.05	(0.94-1.17)	###	4.27E-01	0.96	(0.85-1.09)	0.06	4.98E-01	1.02	(0.95-1.09)	0.03	6.44E-01	0.99	(0.91-1.07)	0.04	7.41E-01
rs4440604_G	chr8	104278607	G	A	1.06	(0.95-1.17)	###	3.07E-01	0.99	(0.88-1.12)	0.06	9.26E-01	0.99	(0.93-1.06)	0.03	7.75E-01	1.08	(1-1.17)	0.04	4.34E-02
rs10119345_C	chr9	110898705	C	T	1.00	(0.85-1.17)	###	9.86E-01	1.06	(0.9-1.26)	0.08	4.58E-01	0.97	(0.89-1.06)	0.04	5.28E-01	1.03	(0.92-1.15)	0.06	5.61E-01
rs10812343_G	chr9	2616875	G	A	1.00	(0.82-1.23)	###	9.61E-01	1.06	(0.94-1.2)	0.06	3.47E-01	0.96	(0.9-1.02)	0.03	1.70E-01	1.02	(0.88-1.17)	0.07	8.10E-01
rs2297876_G	chr9	35604048	G	A	0.78	(0.67-0.92)	###	2.39E-03	1.02	(0.86-1.22)	0.09	8.01E-01	1.05	(0.95-1.15)	0.05	3.42E-01	1.10	(0.98-1.23)	0.06	1.10E-01
rs72761985_G	chr9	113517421	G	T	0.91	(0.7-1.18)	###	4.55E-01	1.21	(0.81-1.79)	0.20	3.53E-01	1.10	(0.91-1.32)	0.09	3.25E-01	1.17	(0.97-1.42)	0.10	1.10E-01
rs10883801_C	chr10	102918130	C	A	0.98	(0.87-1.1)	###	6.86E-01	1.03	(0.91-1.17)	0.06	6.06E-01	0.99	(0.93-1.06)	0.03	8.68E-01	0.97	(0.89-1.05)	0.04	4.62E-01
rs10904183_A	chr10	4097576	A	G	0.93	(0.81-1.07)	###	3.26E-01	1.05	(0.91-1.2)	0.07	5.20E-01	0.99	(0.92-1.06)	0.04	7.09E-01	0.98	(0.88-1.09)	0.05	6.86E-01
rs10995246_C	chr10	62632086	C	A	1.06	(0.95-1.19)	###	3.00E-01	1.07	(0.85-1.35)	0.12	5.71E-01	0.99	(0.88-1.12)	0.06	9.08E-01	1.10	(1.01-1.19)	0.04	2.90E-02
rs12098858_T	chr10	67012714	T	C	1.00	(0.88-1.13)	###	9.88E-01	1.15	(0.96-1.37)	0.09	1.28E-01	0.98	(0.9-1.08)	0.05	7.17E-01	0.98	(0.9-1.07)	0.05	6.48E-01
rs12763743_A	chr10	95832399	A	T	1.03	(0.88-1.21)	###	7.29E-01	1.12	(0.94-1.33)	0.09	2.10E-01	1.02	(0.93-1.12)	0.05	6.65E-01	0.90	(0.81-1.01)	0.06	6.71E-02
rs1750737_A	chr10	11113369	A	G	0.95	(0.79-1.14)	###	5.69E-01	1.07	(0.88-1.28)	0.09	4.97E-01	1.05	(0.95-1.17)	0.05	2.98E-01	0.98	(0.86-1.12)	0.07	7.78E-01
rs2387397_C	chr10	6348230	C	G	1.02	(0.9-1.17)	###	7.46E-01	0.90	(0.73-1.11)	0.11	3.32E-01	0.97	(0.87-1.09)	0.06	6.49E-01	0.90	(0.82-0.99)	0.05	2.97E-02
rs3750619_G	chr10	110919278	G	A	0.78	(0.55-1.09)	###	1.42E-01	0.95	(0.76-1.18)	0.11	6.42E-01	0.97	(0.87-1.09)	0.06	6.11E-01	0.95	(0.75-1.21)	0.12	6.78E-01
rs3793786_A	chr10	49535042	A	G	1.11	(0.97-1.26)	###	1.19E-01	1.00	(0.87-1.14)	0.07	9.69E-01	1.02	(0.95-1.09)	0.04	6.17E-01	0.93	(0.85-1.01)	0.04	9.88E-02
rs670446_C	chr11	107602007	C	T	0.89	(0.75-1.06)	###	1.96E-01	0.98	(0.77-1.23)	0.12	8.42E-01	1.01	(0.89-1.14)	0.06	9.15E-01	0.94	(0.82-1.07)	0.07	3.10E-01
rs871699_T	chr11	18106132	T	C	1.01	(0.86-1.18)	###	9.39E-01	1.22	(0.92-1.63)	0.15	1.64E-01	0.99	(0.85-1.15)	0.08	8.49E-01	1.04	(0.93-1.17)	0.06	4.95E-01
rs10862993_T	chr12	85531560	T	C	1.05	(0.94-1.17)	###	4.17E-01	1.05	(0.93-1.18)	0.06	4.38E-01	1.01	(0.95-1.08)	0.03	7.37E-01	1.05	(0.97-1.14)	0.04	2.26E-01

rs12300068_G	chr12	79087591	G	A	0.98	(0.84-1.16)	###	8.33E-01	1.04	(0.88-1.22)	0.08	6.51E-01	1.04	(0.95-1.13)	0.04	4.05E-01	0.95	(0.84-1.07)	0.06	3.71E-01
rs2286576_T	chr12	4604842	T	G	1.00	(0.9-1.11)	###	9.68E-01	1.09	(0.96-1.25)	0.07	1.91E-01	1.01	(0.94-1.09)	0.04	7.55E-01	0.97	(0.9-1.05)	0.04	4.93E-01
rs2638263_A	chr12	40152998	A	T	1.00	(0.9-1.11)	###	9.95E-01	0.95	(0.84-1.07)	0.06	3.68E-01	1.07	(1-1.14)	0.03	4.13E-02	0.96	(0.89-1.04)	0.04	3.08E-01
rs7307718_C	chr12	40632248	C	T	0.95	(0.86-1.06)	###	3.95E-01	0.94	(0.84-1.05)	0.06	2.76E-01	0.98	(0.92-1.04)	0.03	4.43E-01	0.98	(0.9-1.05)	0.04	5.28E-01
rs7309003_C	chr12	97358012	C	G	0.94	(0.84-1.05)	###	2.83E-01	0.91	(0.81-1.03)	0.06	1.57E-01	0.95	(0.89-1.01)	0.03	1.23E-01	0.98	(0.91-1.07)	0.04	7.06E-01
rs961320_C	chr12	48904081	C	T	0.93	(0.83-1.05)	###	2.69E-01	1.04	(0.92-1.19)	0.07	5.21E-01	0.99	(0.93-1.06)	0.04	8.43E-01	1.09	(1-1.19)	0.05	6.37E-02
rs1923909_T	chr13	24124980	T	C	1.07	(0.86-1.33)	###	5.63E-01	0.86	(0.68-1.07)	0.12	1.85E-01	1.09	(0.97-1.22)	0.06	1.68E-01	1.03	(0.88-1.2)	0.08	7.49E-01
rs45465203_G	chr13	48038078	G	A	1.03	(0.83-1.28)	###	8.02E-01	0.98	(0.81-1.18)	0.10	8.22E-01	0.97	(0.88-1.07)	0.05	5.87E-01	0.89	(0.76-1.04)	0.08	1.28E-01
rs12594789_G	chr15	65304862	G	A	1.09	(0.96-1.23)	###	1.86E-01	1.12	(0.99-1.28)	0.06	7.23E-02	1.05	(0.98-1.12)	0.03	1.78E-01	0.94	(0.86-1.03)	0.04	1.64E-01
rs3825776_T	chr15	58454631	T	C	1.00	(0.86-1.16)	###	9.92E-01	0.94	(0.77-1.14)	0.10	5.12E-01	1.03	(0.94-1.14)	0.05	4.79E-01	1.05	(0.94-1.17)	0.06	3.87E-01
rs4408496_T	chr15	70463694	T	C	1.12	(0.82-1.56)	###	4.80E-01	0.93	(0.66-1.3)	0.17	6.84E-01	1.01	(0.85-1.21)	0.09	8.78E-01	0.93	(0.75-1.16)	0.11	5.18E-01
rs58583822_G	chr15	59165936	G	A	0.99	(0.87-1.13)	###	8.76E-01	0.94	(0.81-1.09)	0.08	4.40E-01	1.01	(0.94-1.09)	0.04	7.96E-01	1.02	(0.93-1.12)	0.05	6.74E-01
rs8035605_G	chr15	35756386	G	T	0.99	(0.88-1.1)	###	8.15E-01	1.02	(0.9-1.16)	0.07	7.39E-01	0.98	(0.91-1.04)	0.03	4.92E-01	1.00	(0.93-1.09)	0.04	9.32E-01
rs2059292_A	chr16	62423133	A	T	1.04	(0.93-1.17)	###	4.70E-01	0.97	(0.85-1.1)	0.06	6.06E-01	1.03	(0.97-1.1)	0.03	3.87E-01	1.03	(0.95-1.12)	0.04	4.85E-01
rs35787586_C	chr16	19067848	C	A	1.01	(0.86-1.19)	###	9.13E-01	1.00	(0.81-1.24)	0.11	9.99E-01	0.95	(0.87-1.04)	0.04	2.57E-01	1.08	(0.96-1.22)	0.06	2.09E-01
rs11657238_G	chr17	48908272	G	A	0.89	(0.76-1.05)	###	1.55E-01	1.15	(1-1.31)	0.07	4.74E-02	0.98	(0.91-1.05)	0.04	6.01E-01	1.08	(0.96-1.22)	0.06	1.78E-01
rs1380180_A	chr17	15289882	A	C	1.00	(0.9-1.12)	###	9.63E-01	1.21	(1.05-1.38)	0.07	6.84E-03	1.01	(0.94-1.08)	0.04	8.81E-01	0.97	(0.9-1.05)	0.04	4.83E-01
rs34038545_G	chr17	47067541	G	C	1.01	(0.91-1.11)	###	8.91E-01	0.97	(0.86-1.1)	0.06	6.43E-01	0.99	(0.93-1.05)	0.03	6.71E-01	0.98	(0.92-1.06)	0.04	6.58E-01
rs4796758_T	chr17	42017501	T	G	0.93	(0.78-1.12)	###	4.23E-01	0.98	(0.83-1.15)	0.08	7.82E-01	1.01	(0.93-1.09)	0.04	8.58E-01	0.97	(0.85-1.11)	0.07	6.75E-01
rs1039143_A	chr18	32714710	A	G	1.03	(0.91-1.17)	###	6.19E-01	1.01	(0.87-1.16)	0.07	9.32E-01	1.01	(0.94-1.08)	0.04	8.67E-01	0.95	(0.87-1.04)	0.04	2.68E-01
rs689384_C	chr18	56651223	C	T	1.04	(0.86-1.24)	###	6.90E-01	0.96	(0.81-1.14)	0.09	6.42E-01	0.94	(0.86-1.03)	0.05	1.84E-01	1.05	(0.92-1.2)	0.07	4.70E-01
rs1073378_C	chr20	13247424	C	T	0.99	(0.88-1.11)	###	8.55E-01	0.97	(0.84-1.13)	0.08	7.04E-01	0.98	(0.9-1.06)	0.04	5.45E-01	0.95	(0.88-1.04)	0.04	2.64E-01
rs16993008_G	chr20	47861743	G	C	1.11	(0.95-1.3)	###	1.75E-01	0.93	(0.76-1.14)	0.10	4.76E-01	0.96	(0.87-1.07)	0.05	4.98E-01	1.07	(0.95-1.19)	0.06	2.58E-01
rs62200158_C	chr20	62926554	C	T	0.92	(0.81-1.04)	###	1.64E-01	1.04	(0.78-1.38)	0.15	7.93E-01	1.00	(0.86-1.17)	0.08	9.95E-01	0.97	(0.89-1.06)	0.04	4.98E-01
rs74364610_G	chr21	42557163	G	A	1.10	(0.91-1.33)	###	3.36E-01	1.29	(1.02-1.63)	0.12	3.20E-02	1.01	(0.9-1.14)	0.06	8.24E-01	1.05	(0.92-1.2)	0.07	4.69E-01
rs2267393_G	chr22	38705614	G	C	0.98	(0.88-1.11)	###	7.87E-01	0.89	(0.77-1.04)	0.08	1.44E-01	0.97	(0.9-1.05)	0.04	5.01E-01	0.99	(0.91-1.08)	0.04	8.07E-01

Abbreviations: KPNC, Kaiser Permanente Northern California; UKB, UK Biobank; FDR, false discovery rate; GIV, genetic instrumental variable; VDR-BV, vitamin D receptor binding variant

Table S7. Meta-analysis associations between vitamin D receptor binding variants (VDR-BV) and multiple sclerosis.

VDR-BV	chr hg19	bp hg19	effect allele	other allele	OR	95% CI	p.value	FDR	Q	df.Q	pval.Q	I2	I2_ci
rs10797448_C	chr1	233348941	C	T	1.02	(0.94-1.1)	6.87E-01	0.949	6.19	3	0.10	0.52	[0-0.84]
rs10800934_G	chr1	203429502	G	C	1.02	(0.97-1.06)	4.82E-01	0.929	1.51	3	0.68	0.00	[0-0.7]
rs11263852_G	chr1	36088841	G	C	0.92	(0.82-1.03)	1.64E-01	0.894	5.36	3	0.15	0.44	[0-0.81]
rs11585739_C	chr1	230110153	C	T	0.99	(0.94-1.04)	5.78E-01	0.929	0.63	3	0.89	0.00	[0-0.27]
rs12021542_T	chr1	230981596	T	A	0.98	(0.93-1.03)	4.97E-01	0.929	2.34	3	0.51	0.00	[0-0.8]
rs12048389_T	chr1	106996101	T	C	0.92	(0.87-0.99)	1.77E-02	0.657	1.39	3	0.71	0.00	[0-0.67]
rs12144635_T	chr1	173277123	T	C	1.12	(0.97-1.28)	1.24E-01	0.870	6.33	3	0.10	0.53	[0-0.84]
rs2331903_T	chr1	180981733	T	C	0.96	(0.87-1.07)	4.80E-01	0.929	6.12	3	0.11	0.51	[0-0.84]
rs3738504_C	chr1	42767136	C	G	1.02	(0.97-1.07)	4.24E-01	0.929	0.37	3	0.95	0.00	[0-0]
rs4916354_C	chr1	173415688	C	T	0.99	(0.89-1.1)	8.14E-01	0.984	7.55	3	0.06	0.60	[0-0.87]
rs56077241_C	chr1	167715577	C	A	1.03	(0.93-1.14)	6.00E-01	0.929	1.16	3	0.76	0.00	[0-0.6]
rs664182_A	chr1	33127286	A	G	1.03	(0.98-1.08)	2.28E-01	0.894	2.77	3	0.43	0.00	[0-0.83]
rs6687177_G	chr1	157182009	G	C	0.99	(0.94-1.04)	6.51E-01	0.929	3.42	3	0.33	0.12	[0-0.87]
rs10179357_A	chr2	205368061	A	C	1.02	(0.95-1.08)	6.34E-01	0.929	1.05	3	0.79	0.00	[0-0.56]
rs12613956_T	chr2	44244270	T	G	1.02	(0.96-1.07)	5.66E-01	0.929	2.94	3	0.40	0.00	[0-0.84]
rs12624197_C	chr2	241315322	C	G	0.99	(0.94-1.03)	5.83E-01	0.929	1.24	3	0.74	0.00	[0-0.63]
rs13021990_T	chr2	13853805	T	C	1.04	(0.97-1.1)	2.46E-01	0.894	0.36	3	0.95	0.00	[0-0]
rs2241876_G	chr2	233278088	G	T	0.99	(0.95-1.04)	7.73E-01	0.962	1.81	3	0.61	0.00	[0-0.75]
rs3813228_A	chr2	73780218	A	G	1.02	(0.96-1.08)	5.92E-01	0.929	0.70	3	0.87	0.00	[0-0.35]
rs4413108_C	chr2	126864471	C	T	1.03	(0.94-1.13)	5.52E-01	0.929	4.61	3	0.20	0.35	[0-0.77]
rs4670248_T	chr2	38262143	T	C	0.99	(0.94-1.05)	7.67E-01	0.962	1.65	3	0.65	0.00	[0-0.72]
rs55792977_T	chr2	65423730	T	G	1.05	(1-1.09)	3.15E-02	0.657	0.78	3	0.85	0.00	[0-0.41]
rs6436813_G	chr2	228683248	G	C	1.01	(0.96-1.06)	7.59E-01	0.962	0.55	3	0.91	0.00	[0-0.16]
rs6727980_T	chr2	20868589	T	G	0.93	(0.86-1.01)	6.91E-02	0.833	2.20	3	0.53	0.00	[0-0.79]
rs13086176_C	chr3	57100218	C	T	1.00	(0.93-1.07)	8.94E-01	0.987	6.05	3	0.11	0.50	[0-0.84]
rs13098781_G	chr3	18716927	G	A	1.04	(0.96-1.11)	3.57E-01	0.929	4.09	3	0.25	0.27	[0-0.72]
rs13324462_A	chr3	197713714	A	G	1.00	(0.9-1.11)	9.90E-01	0.996	4.87	3	0.18	0.38	[0-0.79]
rs2279908_C	chr3	44729636	C	T	1.02	(0.98-1.06)	4.28E-01	0.929	0.80	3	0.85	0.00	[0-0.43]
rs2574711_C	chr3	11626292	C	T	1.03	(0.9-1.17)	7.16E-01	0.960	6.25	3	0.10	0.52	[0-0.84]
rs2881514_A	chr3	16512173	A	G	1.10	(1.05-1.15)	9.40E-05	0.011	0.21	3	0.98	0.00	[0-0]
rs62248690_G	chr3	43181246	G	A	0.93	(0.83-1.05)	2.33E-01	0.894	1.94	3	0.59	0.00	[0-0.76]
rs62410372_A	chr3	179260754	A	G	1.01	(0.97-1.06)	5.71E-01	0.929	1.94	3	0.58	0.00	[0-0.76]
rs9818027_G	chr3	189489558	G	A	0.98	(0.93-1.04)	5.75E-01	0.929	1.03	3	0.79	0.00	[0-0.55]
rs998909_A	chr3	52771077	A	G	1.02	(0.95-1.1)	6.06E-01	0.929	1.16	3	0.76	0.00	[0-0.6]
rs11132491_G	chr4	187913080	G	T	1.02	(0.92-1.12)	7.23E-01	0.960	5.79	3	0.12	0.48	[0-0.83]
rs11544037_C	chr4	158667824	C	A	1.06	(0.94-1.2)	3.39E-01	0.929	####	3	0.01	0.74	[0.26-0.91]
rs11729497_G	chr4	125630228	G	C	1.04	(0.95-1.14)	3.84E-01	0.929	####	3	0.01	0.72	[0.19-0.9]
rs11733032_G	chr4	78639073	G	A	0.98	(0.93-1.02)	3.03E-01	0.929	1.81	3	0.61	0.00	[0-0.75]
rs34035218_C	chr4	37562171	C	T	1.03	(0.99-1.07)	1.89E-01	0.894	1.69	3	0.64	0.00	[0-0.73]
rs10069743_T	chr5	129088984	T	C	1.08	(1-1.17)	5.71E-02	0.799	3.03	3	0.39	0.01	[0-0.85]
rs1430204_C	chr5	88382815	C	T	1.05	(0.92-1.19)	4.72E-01	0.929	9.81	3	0.02	0.69	[0.12-0.89]
rs2170502_G	chr5	7911030	G	A	0.99	(0.95-1.04)	7.28E-01	0.960	3.37	3	0.34	0.11	[0-0.86]
rs257916_T	chr5	129085865	T	G	1.02	(0.98-1.07)	3.06E-01	0.929	2.47	3	0.48	0.00	[0-0.81]
rs4276369_G	chr5	61330048	G	A	1.03	(0.95-1.12)	4.84E-01	0.929	6.38	3	0.09	0.53	[0-0.84]
rs4703608_A	chr5	73654711	A	G	1.02	(0.96-1.08)	5.12E-01	0.929	3.09	3	0.38	0.03	[0-0.85]
rs55811049_A	chr5	118988603	A	C	1.06	(0.99-1.12)	8.18E-02	0.833	1.01	3	0.80	0.00	[0-0.55]
rs67061065_C	chr5	36242110	C	T	0.97	(0.93-1.02)	2.33E-01	0.894	2.26	3	0.52	0.00	[0-0.8]
rs712615_T	chr5	116885938	T	A	0.98	(0.94-1.03)	4.58E-01	0.929	1.81	3	0.61	0.00	[0-0.75]
rs72775970_C	chr5	97643953	C	T	0.99	(0.93-1.06)	7.62E-01	0.962	0.33	3	0.95	0.00	[0-0]
rs10949389_A	chr6	16942833	A	G	0.98	(0.93-1.02)	3.07E-01	0.929	1.64	3	0.65	0.00	[0-0.72]
rs12214623_T	chr6	113065866	T	C	1.00	(0.91-1.1)	9.75E-01	0.996	9.28	3	0.03	0.68	[0.06-0.89]
rs1782437_G	chr6	90425069	G	A	1.00	(0.92-1.08)	9.80E-01	0.996	0.95	3	0.81	0.00	[0-0.52]
rs178399_G	chr6	2791449	G	A	0.94	(0.86-1.01)	9.65E-02	0.870	####	3	0.01	0.72	[0.22-0.9]
rs2531804_A	chr6	28443526	A	G	0.82	(0.73-0.92)	6.40E-04	0.036	####	3	0.02	0.70	[0.14-0.9]
rs274436_C	chr6	104326558	C	T	1.03	(0.97-1.08)	3.27E-01	0.929	3.70	3	0.30	0.19	[0-0.88]
rs3798259_G	chr6	45921171	G	T	0.95	(0.86-1.04)	2.47E-01	0.894	8.14	3	0.04	0.63	[0-0.88]
rs396360_C	chr6	13260221	C	T	1.00	(0.93-1.07)	9.96E-01	0.996	1.76	3	0.62	0.00	[0-0.74]
rs66773803_C	chr6	130967385	C	T	0.98	(0.91-1.05)	5.86E-01	0.929	1.23	3	0.75	0.00	[0-0.63]
rs9347904_T	chr6	158032179	T	C	0.93	(0.86-1.01)	1.04E-01	0.870	7.86	3	0.05	0.62	[0-0.87]
rs9348121_C	chr6	166341524	C	A	0.99	(0.95-1.04)	7.45E-01	0.962	1.95	3	0.58	0.00	[0-0.76]
rs9493768_T	chr6	133953159	T	A	1.01	(0.91-1.11)	8.97E-01	0.987	0.46	3	0.93	0.00	[0-0]
rs10232857_C	chr7	55533643	C	T	0.93	(0.87-1)	4.89E-02	0.783	1.76	3	0.62	0.00	[0-0.74]
rs10951263_G	chr7	30163611	G	A	0.99	(0.93-1.07)	8.52E-01	0.984	4.09	3	0.25	0.27	[0-0.72]
rs2686823_C	chr7	47937388	C	T	0.99	(0.93-1.04)	6.43E-01	0.929	5.16	3	0.16	0.42	[0-0.8]
rs2708881_C	chr7	47937496	C	A	1.02	(0.93-1.12)	6.56E-01	0.929	5.19	3	0.16	0.42	[0-0.81]
rs34571901_A	chr7	37038985	A	G	1.01	(0.91-1.12)	8.63E-01	0.986	5.18	3	0.16	0.42	[0-0.81]
rs698631_C	chr7	56195405	C	T	0.98	(0.91-1.05)	5.32E-01	0.929	1.68	3	0.64	0.00	[0-0.73]
rs1347322_G	chr8	102536334	G	A	0.96	(0.91-1.02)	1.70E-01	0.894	1.93	3	0.59	0.00	[0-0.76]

rs1389206_G	chr8	71012627	G	A	1.00	(0.96-1.05)	8.44E-01	0.984	1.37	3	0.71	0.00	[0-0.67]
rs4440604_G	chr8	104278607	G	A	1.03	(0.98-1.08)	2.30E-01	0.894	3.56	3	0.31	0.16	[0-0.87]
rs10119345_C	chr9	110898705	C	T	1.00	(0.95-1.06)	8.99E-01	0.987	1.27	3	0.74	0.00	[0-0.64]
rs10812343_G	chr9	2616875	G	A	0.98	(0.94-1.04)	5.46E-01	0.929	2.46	3	0.48	0.00	[0-0.81]
rs2297876_G	chr9	35604048	G	A	0.99	(0.86-1.13)	8.52E-01	0.984	####	3	0.01	0.76	[0.35-0.91]
rs72761985_G	chr9	113517421	G	T	1.09	(0.97-1.22)	1.37E-01	0.894	2.74	3	0.43	0.00	[0-0.83]
rs10883801_C	chr10	102918130	C	A	0.99	(0.95-1.03)	6.39E-01	0.929	0.78	3	0.85	0.00	[0-0.41]
rs10904183_A	chr10	4097576	A	G	0.99	(0.94-1.04)	5.62E-01	0.929	1.35	3	0.72	0.00	[0-0.66]
rs10995246_C	chr10	62632086	C	A	1.06	(1-1.13)	3.52E-02	0.657	1.74	3	0.63	0.00	[0-0.74]
rs12098858_T	chr10	67012714	T	C	1.00	(0.95-1.05)	9.74E-01	0.996	2.66	3	0.45	0.00	[0-0.83]
rs12763743_A	chr10	95832399	A	T	1.00	(0.92-1.09)	9.86E-01	0.996	5.21	3	0.16	0.42	[0-0.81]
rs1750737_A	chr10	11113369	A	G	1.02	(0.95-1.09)	5.46E-01	0.929	1.58	3	0.66	0.00	[0-0.71]
rs2387397_C	chr10	6348230	C	G	0.95	(0.89-1.01)	7.70E-02	0.833	2.85	3	0.42	0.00	[0-0.84]
rs3750619_G	chr10	110919278	G	A	0.95	(0.87-1.04)	2.56E-01	0.894	1.51	3	0.68	0.00	[0-0.7]
rs3793786_A	chr10	49535042	A	G	1.00	(0.94-1.07)	9.21E-01	0.992	5.40	3	0.14	0.44	[0-0.81]
rs670446_C	chr11	107602007	C	T	0.96	(0.89-1.03)	2.47E-01	0.894	1.41	3	0.70	0.00	[0-0.68]
rs871699_T	chr11	18106132	T	C	1.03	(0.95-1.11)	4.44E-01	0.929	1.85	3	0.60	0.00	[0-0.75]
rs10862993_T	chr12	85531560	T	C	1.03	(0.99-1.08)	1.48E-01	0.894	0.74	3	0.86	0.00	[0-0.38]
rs12300068_G	chr12	79087591	G	A	1.01	(0.95-1.07)	8.39E-01	0.984	1.70	3	0.64	0.00	[0-0.73]
rs2286576_T	chr12	4604842	T	G	1.00	(0.96-1.05)	8.34E-01	0.984	2.23	3	0.53	0.00	[0-0.79]
rs2638263_A	chr12	40152998	A	T	1.00	(0.94-1.06)	9.74E-01	0.996	5.79	3	0.12	0.48	[0-0.83]
rs7307718_C	chr12	40632248	C	T	0.97	(0.93-1.01)	1.19E-01	0.870	0.47	3	0.93	0.00	[0-0.02]
rs7309003_C	chr12	97358012	C	G	0.95	(0.91-1)	3.23E-02	0.657	1.10	3	0.78	0.00	[0-0.58]
rs961320_C	chr12	48904081	C	T	1.02	(0.96-1.08)	6.19E-01	0.929	4.69	3	0.20	0.36	[0-0.78]
rs1923909_T	chr13	24124980	T	C	1.03	(0.95-1.13)	4.79E-01	0.929	3.37	3	0.34	0.11	[0-0.86]
rs45465203_G	chr13	48038078	G	A	0.96	(0.89-1.03)	2.67E-01	0.905	1.49	3	0.69	0.00	[0-0.69]
rs12594789_G	chr15	65304862	G	A	1.04	(0.96-1.12)	3.26E-01	0.929	6.97	3	0.07	0.57	[0-0.86]
rs3825776_T	chr15	58454631	T	C	1.02	(0.96-1.09)	4.66E-01	0.929	1.15	3	0.77	0.00	[0-0.6]
rs4408496_T	chr15	70463694	T	C	0.99	(0.88-1.12)	8.95E-01	0.987	1.09	3	0.78	0.00	[0-0.58]
rs58583822_G	chr15	59165936	G	A	1.00	(0.95-1.05)	9.37E-01	0.996	0.86	3	0.84	0.00	[0-0.46]
rs8035605_G	chr15	35756386	G	T	0.99	(0.95-1.04)	6.95E-01	0.949	0.49	3	0.92	0.00	[0-0.07]
rs2059292_A	chr16	62423133	A	T	1.02	(0.98-1.07)	2.93E-01	0.929	0.92	3	0.82	0.00	[0-0.5]
rs35787586_C	chr16	19067848	C	A	1.00	(0.94-1.06)	9.14E-01	0.992	2.86	3	0.41	0.00	[0-0.84]
rs11657238_G	chr17	48908272	G	A	1.02	(0.93-1.12)	6.35E-01	0.929	7.76	3	0.05	0.61	[0-0.87]
rs1380180_A	chr17	15289882	A	C	1.03	(0.95-1.11)	4.73E-01	0.929	7.45	3	0.06	0.60	[0-0.87]
rs34038545_G	chr17	47067541	G	C	0.99	(0.95-1.03)	5.35E-01	0.929	0.22	3	0.97	0.00	[0-0]
rs4796758_T	chr17	42017501	T	G	0.99	(0.93-1.05)	6.68E-01	0.935	0.74	3	0.86	0.00	[0-0.38]
rs1039143_A	chr18	32714710	A	G	0.99	(0.95-1.04)	7.81E-01	0.962	1.44	3	0.70	0.00	[0-0.68]
rs689384_C	chr18	56651223	C	T	0.98	(0.92-1.05)	5.50E-01	0.929	2.31	3	0.51	0.00	[0-0.8]
rs1073378_C	chr20	13247424	C	T	0.97	(0.92-1.02)	2.20E-01	0.894	0.29	3	0.96	0.00	[0-0]
rs16993008_G	chr20	47861743	G	C	1.02	(0.95-1.1)	6.02E-01	0.929	3.75	3	0.29	0.20	[0-0.88]
rs62200158_C	chr20	62926554	C	T	0.96	(0.91-1.03)	2.53E-01	0.894	1.16	3	0.76	0.00	[0-0.61]
rs74364610_G	chr21	42557163	G	A	1.07	(0.98-1.16)	1.13E-01	0.870	3.45	3	0.33	0.13	[0-0.87]
rs2267393_G	chr22	38705614	G	C	0.97	(0.93-1.02)	2.52E-01	0.894	1.41	3	0.70	0.00	[0-0.67]

Abbreviations: KPNC, Kaiser Permanente Northern California; UKB, UK Biobank; FDR, false discovery rate; GIV, genetic instrumental variable; VDR-BV, vitamin D receptor binding varian

Table S8. Associations for interactions between 25(OH)D GIVs and vitamin D receptor binding variants (VDR-BV) for multiple sclerosis within each study.

						KPNC				GSA				Human OMNI				UKB			
VDR-BV	chr hg19	bp hg19	effect allele	other allele	25(OH)D GIV[a]	OR	95% CI	SE	p.value	OR	95% CI	SE	p.value	OR	95% CI	SE	p.value	OR	95% CI	SE	p.value
rs10797448	chr1	233348941	C	T	Jiang et al.	0.74	(0.15-3.62)	0.81	7.13E-01	0.87	(0.05-15.3)	1.47	9.22E-01	1.05	(0.23-4.78)	0.77	9.50E-01	0.67	(0.22-2.08)	0.58	4.90E-01
rs10797448	chr1	233348941	C	T	Revez et al.	0.79	(0.44-1.44)	0.30	4.45E-01	1.37	(0.46-4.04)	0.55	5.69E-01	0.78	(0.45-1.36)	0.28	3.82E-01	0.81	(0.53-1.24)	0.21	3.32E-01
rs10800934	chr1	203429502	G	C	Jiang et al.	0.47	(0.1-2.17)	0.78	3.35E-01	0.59	(0.09-3.82)	0.95	5.80E-01	0.82	(0.32-2.08)	0.48	6.70E-01	1.01	(0.34-3.03)	0.56	9.84E-01
rs10800934	chr1	203429502	G	C	Revez et al.	0.72	(0.41-1.27)	0.29	2.52E-01	1.14	(0.57-2.31)	0.36	7.10E-01	1.10	(0.78-1.54)	0.17	5.98E-01	1.11	(0.74-1.67)	0.21	6.01E-01
rs11263852	chr1	36088841	G	C	Jiang et al.	0.44	(0.01-16.58)	1.85	6.59E-01	0.14	(0.01-1.98)	1.34	1.45E-01	0.44	(0.11-1.76)	0.71	2.46E-01	6.74	(0.46-99.66)	1.37	1.65E-01
rs11263852	chr1	36088841	G	C	Revez et al.	0.45	(0.11-1.74)	0.70	2.45E-01	0.69	(0.25-1.86)	0.51	4.60E-01	0.85	(0.51-1.41)	0.26	5.29E-01	1.42	(0.52-3.84)	0.51	4.93E-01
rs11585739	chr1	230110153	C	T	Jiang et al.	2.85	(0.14-57.99)	1.54	4.96E-01	0.70	(0.11-4.46)	0.94	7.06E-01	1.38	(0.54-3.51)	0.48	4.99E-01	0.16	(0.02-1.4)	1.12	9.74E-02
rs11585739	chr1	230110153	C	T	Revez et al.	1.31	(0.42-4.05)	0.58	6.43E-01	0.90	(0.45-1.8)	0.35	7.74E-01	0.97	(0.69-1.37)	0.17	8.76E-01	0.74	(0.33-1.67)	0.41	4.72E-01
rs12021542	chr1	230981596	T	A	Jiang et al.	0.39	(0.06-2.48)	0.94	3.20E-01	0.52	(0.07-4.03)	1.04	5.33E-01	0.56	(0.19-1.67)	0.56	3.01E-01	1.59	(0.4-6.3)	0.70	5.08E-01
rs12021542	chr1	230981596	T	A	Revez et al.	0.68	(0.34-1.36)	0.35	2.76E-01	1.63	(0.76-3.53)	0.39	2.14E-01	0.98	(0.66-1.45)	0.20	9.14E-01	1.10	(0.67-1.83)	0.26	7.00E-01
rs12048389	chr1	106996101	T	C	Jiang et al.	2.16	(0.17-27.33)	1.30	5.53E-01	1.22	(0.09-16.51)	1.34	8.83E-01	1.87	(0.46-7.59)	0.72	3.82E-01	1.91	(0.32-11.53)	0.92	4.81E-01
rs12048389	chr1	106996101	T	C	Revez et al.	1.15	(0.45-2.94)	0.48	7.64E-01	1.09	(0.41-2.82)	0.49	8.67E-01	0.92	(0.55-1.54)	0.26	7.56E-01	1.72	(0.89-3.33)	0.34	1.06E-01
rs12144635	chr1	173277123	T	C	Jiang et al.	0.18	(0-18.03)	2.36	4.63E-01	0.26	(0.02-4.16)	1.42	3.47E-01	0.29	(0.07-1.25)	0.75	9.74E-02	0.21	(0.01-5.41)	1.65	3.49E-01
rs12144635	chr1	173277123	T	C	Revez et al.	0.38	(0.07-2.09)	0.88	2.64E-01	1.57	(0.56-4.38)	0.52	3.89E-01	1.38	(0.81-2.36)	0.27	2.41E-01	0.72	(0.22-2.38)	0.61	5.93E-01
rs2331903	chr1	180981733	T	C	Jiang et al.	0.55	(0.01-39.19)	2.17	7.85E-01	3.39	(0.6-19.28)	0.89	1.69E-01	0.51	(0.2-1.26)	0.46	1.43E-01	2.57	(0.12-54.35)	1.56	5.45E-01
rs2331903	chr1	180981733	T	C	Revez et al.	0.70	(0.14-3.42)	0.81	6.58E-01	2.43	(1.23-4.81)	0.35	1.07E-02	0.97	(0.69-1.36)	0.17	8.68E-01	1.55	(0.51-4.75)	0.57	4.44E-01
rs3738504	chr1	42767136	C	G	Jiang et al.	1.73	(0.16-18.64)	1.21	6.51E-01	0.35	(0.06-2.03)	0.90	2.41E-01	1.53	(0.61-3.86)	0.47	3.64E-01	1.07	(0.2-5.77)	0.86	9.41E-01
rs3738504	chr1	42767136	C	G	Revez et al.	1.03	(0.43-2.48)	0.45	9.48E-01	0.98	(0.51-1.88)	0.33	9.49E-01	1.19	(0.85-1.68)	0.17	3.04E-01	0.91	(0.49-1.7)	0.32	7.71E-01
rs4916354	chr1	173415688	C	T	Jiang et al.	0.68	(0.04-12.14)	1.47	7.92E-01	0.47	(0.06-3.49)	1.02	4.65E-01	1.54	(0.52-4.59)	0.56	4.38E-01	1.59	(0.21-11.93)	1.03	6.51E-01
rs4916354	chr1	173415688	C	T	Revez et al.	1.09	(0.38-3.13)	0.54	8.74E-01	1.14	(0.53-2.43)	0.39	7.32E-01	1.21	(0.81-1.81)	0.20	3.55E-01	1.49	(0.71-3.11)	0.38	2.90E-01
rs56077241	chr1	167715577	C	A	Jiang et al.	0.95	(0.05-18.29)	1.51	9.76E-01	0.93	(0-687.6)	3.34	9.83E-01	0.80	(0.03-20.4)	1.65	8.90E-01	1.63	(0.2-13.39)	1.07	6.48E-01
rs56077241	chr1	167715577	C	A	Revez et al.	1.13	(0.38-3.38)	0.56	8.21E-01	2.16	(0.2-23.45)	1.21	5.24E-01	0.78	(0.24-2.53)	0.60	6.81E-01	1.13	(0.52-2.47)	0.40	7.55E-01
rs664182	chr1	33127286	A	G	Jiang et al.	0.24	(0.03-1.92)	1.07	1.77E-01	4.14	(0.52-32.9)	1.06	1.79E-01	0.49	(0.18-1.33)	0.51	1.61E-01	0.33	(0.07-1.42)	0.75	1.35E-01
rs664182	chr1	33127286	A	G	Revez et al.	0.80	(0.37-1.75)	0.40	5.84E-01	1.64	(0.75-3.57)	0.40	2.13E-01	1.03	(0.71-1.5)	0.19	8.63E-01	0.74	(0.43-1.27)	0.28	2.72E-01
rs6687177	chr1	157182009	G	C	Jiang et al.	4.57	(0.72-29.13)	0.94	1.08E-01	0.72	(0.12-4.11)	0.89	7.07E-01	0.68	(0.27-1.68)	0.46	4.01E-01	4.14	(1.07-16.05)	0.69	3.99E-02
rs6687177	chr1	157182009	G	C	Revez et al.	1.66	(0.83-3.31)	0.35	1.48E-01	1.03	(0.53-2.02)	0.34	9.29E-01	1.10	(0.79-1.55)	0.17	5.62E-01	1.39	(0.85-2.28)	0.25	1.93E-01
rs10179357	chr2	205368061	A	C	Jiang et al.	3.03	(0.31-29.95)	1.17	3.43E-01	4.16	(0.32-54.91)	1.31	2.75E-01	1.99	(0.51-7.76)	0.69	3.20E-01	0.71	(0.13-3.76)	0.85	6.86E-01
rs10179357	chr2	205368061	A	C	Revez et al.	1.34	(0.57-3.17)	0.44	5.06E-01	2.61	(1.03-6.68)	0.48	4.38E-02	1.38	(0.84-2.27)	0.25	2.02E-01	0.71	(0.39-1.32)	0.31	2.85E-01
rs12613956	chr2	44244270	T	G	Jiang et al.	1.58	(0.2-12.31)	1.05	6.62E-01	0.24	(0.03-2.01)	1.09	1.89E-01	0.26	(0.08-0.81)	0.58	1.99E-02	1.14	(0.25-5.22)	0.78	8.70E-01
rs12613956	chr2	44244270	T	G	Revez et al.	1.27	(0.59-2.74)	0.39	5.35E-01	0.63	(0.28-1.41)	0.41	2.64E-01	0.55	(0.36-0.83)	0.21	4.73E-03	1.23	(0.7-2.17)	0.29	4.66E-01
rs12624197	chr2	241315322	C	G	Jiang et al.	2.49	(0.35-17.71)	1.00	3.62E-01	0.21	(0.04-1.24)	0.90	8.55E-02	0.42	(0.17-1.03)	0.46	5.88E-02	2.14	(0.52-8.81)	0.72	2.92E-01
rs12624197	chr2	241315322	C	G	Revez et al.	1.46	(0.7-3.03)	0.37	3.12E-01	0.55	(0.28-1.08)	0.34	8.33E-02	0.95	(0.68-1.32)	0.17	7.57E-01	1.58	(0.94-2.66)	0.27	8.62E-02
rs13021990	chr2	13853805	T	C	Jiang et al.	2.37	(0.22-25.98)	1.22	4.80E-01	7.73	(0.61-100.14)	1.30	1.16E-01	3.82	(1.01-14.6)	0.68	4.93E-02	0.32	(0.05-1.88)	0.91	2.06E-01
rs13021990	chr2	13853805	T	C	Revez et al.	1.57	(0.64-3.84)	0.46	3.26E-01	3.06	(1.16-8.18)	0.50	2.50E-02	1.23	(0.76-2.01)	0.25	3.97E-01	0.65	(0.34-1.25)	0.33	2.00E-01
rs2241876	chr2	233278088	G	T	Jiang et al.	0.54	(0.09-3.14)	0.90	4.93E-01	1.36	(0.19-9.59)	1.00	7.57E-01	0.83	(0.3-2.28)	0.51	7.22E-01	1.02	(0.29-3.67)	0.65	9.71E-01
rs2241876	chr2	233278088	G	T	Revez et al.	0.70	(0.36-1.36)	0.34	2.93E-01	1.42	(0.68-2.94)	0.37	3.44E-01	0.83	(0.57-1.21)	0.19	3.25E-01	0.92	(0.57-1.47)	0.24	7.22E-01
rs3813228	chr2	73780218	A	G	Jiang et al.	4.61	(0.04-590.21)	2.48	5.37E-01	2.69	(0.39-18.58)	0.98	3.14E-01	1.26	(0.47-3.36)	0.50	6.44E-01	3.23	(0.1-103.24)	1.77	5.07E-01
rs3813228	chr2	73780218	A	G	Revez et al.	1.46	(0.24-9.04)	0.93	6.81E-01	1.19	(0.58-2.44)	0.36	6.27E-01	0.79	(0.55-1.13)	0.18	1.92E-01	2.22	(0.62-7.91)	0.65	2.20E-01
rs4413108	chr2	126864471	C	T	Jiang et al.	0.17	(0.01-2.44)	1.35	1.93E-01	0.35	(0.01-9.34)	1.69	5.38E-01	0.59	(0.12-3)	0.83	5.29E-01	0.57	(0.09-3.65)	0.95	5.55E-01
rs4413108	chr2	126864471	C	T	Revez et al.	0.99	(0.36-2.7)	0.51	9.82E-01	0.65	(0.18-2.25)	0.64	4.98E-01	0.62	(0.34-1.13)	0.31	1.19E-01	0.84	(0.42-1.66)	0.35	6.13E-01
rs4670248	chr2	38262143	T	C	Jiang et al.	0.75	(0.1-5.74)	1.04	7.80E-01	5.95	(0.54-64.8)	1.22	1.45E-01	2.00	(0.57-6.97)	0.64	2.78E-01	2.05	(0.47-9.04)	0.76	3.43E-01
rs4670248	chr2	38262143	T	C	Revez et al.	0.91	(0.42-1.95)	0.39	8.04E-01	1.22	(0.5-2.97)	0.45	6.63E-01	1.13	(0.71-1.78)	0.23	6.14E-01	1.18	(0.68-2.04)	0.28	5.60E-01
rs55792977	chr2	65423730	T	G	Jiang et al.	1.32	(0.27-6.35)	0.80	7.33E-01	0.92	(0.16-5.19)	0.88	9.27E-01	1.52	(0.61-3.84)	0.47	3.71E-01	0.39	(0.12-1.22)	0.58	1.05E-01
rs55792977	chr2	65423730	T	G	Revez et al.	1.23	(0.69-2.22)	0.30	4.85E-01	1.25	(0.64-2.43)	0.34	5.18E-01	1.00	(0.71-1.42)	0.18	9.94E-01	0.68	(0.45-1.04)	0.21	7.54E-02
rs6436813	chr2	228683248	G	C	Jiang et al.	4.23	(0.7-25.64)	0.92	1.17E-01	6.22	(0.69-55.53)	1.12	1.02E-01	1.55	(0.48-5)	0.60	4.68E-01	0.99	(0.27-3.55)	0.65	9.84E-01
rs6436813	chr2	228683248	G	C	Revez et al.	1.50	(0.77-2.94)	0.34	2.37E-01	1.25	(0.55-2.88)	0.42	5.93E-01	1.07	(0.69-1.65)	0.22	7.59E-01	1.04	(0.65-1.67)	0.24	8.68E-01
rs6727980	chr2	20868589	T	G	Jiang et al.	0.78	(0.06-9.69)	1.28	8.48E-01	2.75	(0.08-97.23)	1.82	5.79E-01	1.20	(0.19-7.4)	0.93	8.42E-01	3.56	(0.46-27.3)	1.04	2.22E-01
rs6727980	chr2	20868589	T	G	Revez et al.	0.82	(0.33-2.08)	0.47	6.78E-01	0.61	(0.15-2.36)	0.70	4.74E-01	1.22	(0.63-2.37)	0.34	5.55E-01	1.42	(0.67-3)	0.38	3.63E-01
rs13086176	chr3	57100218	C	T	Jiang et al.	0.44	(0.08-2.43)	0.88	3.44E-01	1.84	(0.29-11.66)	0.94	5.18E-01	0.35	(0.13-0.92)	0.50	3.44E-02	0.64	(0.18-2.25)	0.64	4.85E-01
rs13086176	chr3	57100218	C	T	Revez et al.	0.77	(0.41-1.46)	0.33	4.24E-01	1.35	(0.68-2.71)	0.35	3.89E-01	0.71	(0.5-1.03)	0.19	6.86E-02	0.83	(0.52-1.32)	0.24	4.34E-01
rs13098781	chr3	18716927	G	A	Jiang et al.	1.40	(0.17-11.33)	1.07	7.52E-01	9.90	(0.71-140.74)	1.35	8.89E-02	3.32	(0.79-14.05)	0.73	1.02E-01	0.94	(0.22-4.07)	0.75	9.38E-01
rs13098781	chr3	18716927	G	A	Revez et al.	1.37	(0.63-3)	0.40	4.26E-01	0.83	(0.3-2.28)	0.52	7.10E-01	0.77	(0.45-1.33)	0.28	3.49E-01	1.01	(0.59-1.71)	0.27	9.80E-01
rs13324462	chr3	197713714	A	G	Jiang et al.	5.15	(0.26-102.91)	1.53	2.83E-01	9.96	(0.58-167.85)	1.44	1.11E-01	1.05	(0.22-5.08)	0.81	9.53E-01	2.21	(0.27-18.27)	1.08	4.63E-01
rs13324462	chr3	197713714	A	G	Revez et al.	2.70	(0.88-8.27)	0.57	8.22E-02	2.35	(0.78-7.05)	0.56	1.29E-01	1.16	(0.64-2.1)	0.30	6.30E-01	1.36	(0.63-2.95)	0.40	4.37E-01
rs2279908	chr3	44729636	C	T	Jiang et al.	0.87	(0.19-3.93)	0.77	8.56E-01	2.95	(0.49-18)	0.92	2.39E-01	1.06	(0.42-2.67)	0.47	9.04E-01	0.81	(0.28-2.37)	0.55	7.03E-01
rs2279908	chr3	44729636	C	T	Revez et al.	1.05	(0.6-1.86)	0.29	8.56E-01	1.02	(0.52-1.99)	0.34	9.50E-01	1.03	(0.74-1.45)	0.17	8.47E-01	0.97	(0.66-1.44)	0.20	8.93E-01
rs2574711	chr3	11626292	C	T	Jiang et al.	1.69	(0.05-57.33)	1.80	7.71E-01	0.47	(0.02-12.67)	1.69	6.54E-01	0.46	(0.09-2.35)	0.84	3.49E-01	1.30	(0.08-21.73)	1.44	8.57E-01
rs2574711	chr3	11626292	C	T	Revez et al.	1.14	(0.31-4.21)	0.67	8.46E-01	1.29	(0.37-4.52)	0.64	6.89E-01	1.67	(0.91-3.09)	0.31	1.00E-01	0.82	(0.29-2.29)	0.52	7.05E-01
rs2881514	chr3	16512173	A	G	Jiang et al.	0.79	(0.14-4.47)	0.89	7.88E-01	5.26	(0.71-38.72)	1.02	1.03E-01	1.90	(0.67-5.36)	0.53	2.27E-01	3.15	(0.91-10.84)	0.63	6.94E-02
rs2881514	chr3	16512173	A	G	Revez et al.	0.73	(0.38-1.41)	0.33	3.52E-01	1.65	(0.77-3.54)	0.39	1.95E-01	1.18	(0.81-1.73)	0.19	3.91E-01	1.06	(0.67-1.68)	0.23	7.93E-01
rs62248690	chr3	43181246	G	A	Jiang et al.	0.73	(0.02-32.3)	1.93	8.70E-01	0.14	(0-76.34)	3.25	5.43E-01	0.02	(0-0.62)	1.65	2.46E-02	1.02	(0.09-11.62)	1.24	9.87E-01
rs62248690	chr3	43181246	G	A	Revez et al.	0.72	(0.18-2.97)	0.72	6.53E-01	2.76	(0.24-30.82)	1.24	4.13E-01	0.61	(0.18-2)	0.61	4.12E-01	0.77	(0.31-1.91)	0.46	5.70E-01
rs62410372	chr3	179260754	A	G	Jiang et al.	0.73	(0.15-3.6)	0.82	6.98E-01	5.42	(0.65-45.25)	1.08	1.18E-01	1.69	(0.57-5.03)	0.56	3.47E-01	1.19	(0.38-3.72)	0.58	7.61E-01
rs62410372	chr3	179260754	A	G	Revez et al.	0.81	(0.45-1.47)	0.30	4.96E-01	1.06	(0.47-2.4)	0.41	8.81E-01	1.22	(0.82-1.83)	0.21	3.31E-01	1.25	(0.82-1.9)	0.21	2.96E-01
rs9818027	chr3	189489558	G	A	Jiang et al.	5.48	(0.65-45.96)	1.08	1.17E-01	0.40	(0.05-3.55)	1.11	4.08E-01	0.54	(0.17-1.75)	0.60	3.07E-01	1.96	(0.42-9.23)	0.79	3.92E-01
rs9818027	chr3	189489558	G	A	Revez et al.	2.43	(1.11-5.34)	0.40	2.70E-02	0.63	(0.28-1.42)	0.41	2.66E-01	0.87	(0.56-1.33)	0.22	5.09E-01	1.26	(0.71-2.23)	0.29	4.25E-01
rs998909	chr3	52771077	A	G	Jiang et al.	1.82	(0.12-28.06)	1.39	6.67E-01	0.75	(0.04-15.97)	1.56	8.54E-01	0.28	(0.06-1.36)	0.81	1.14E-01	0.46	(0.06-3.48)	1.03	4.55E-01
rs998909	chr3	52771077	A	G	Revez et al.	1.11	(0.4-3.06)	0.52	8.41E-01	1.11	(0.36-3.45)	0.58	8.58E-01	0.76	(0.43-1.36)	0.30	3.60E-01	0.63	(0.3-1.31)	0.37	2.15E-01
rs11132491	chr4	187913080	G	T	Jiang et al.	0.64	(0.1-3.99)	0.93	6.32E-01	0.00	(0-0.16)	1.90	3.56E-03	0.09	(0.01-0.59)	0.99	1.24E-02	1.40	(0.37-5.32)	0.68	6.22E-01
rs11132491	chr4	187913080	G	T	Revez et al.	0.83	(0.42-1.63)	0.34	5.81E-01	0.25	(0.06-1.05)	0.74	5.76E-02	0.45	(0.22-0.91)	0.36	2.68E-02	1.19	(0.73-1.94)	0.25	4.89E-01
rs11544037	chr4	158667824	C	A	Jiang et al.	0.16	(0.02-1.33)	1.09	9.01E-02	9.83	(0.82-118)	1.27	7.15E-02	5.90	(1.47-23.64)	0.71	1.21E-02	1.83	(0.38-8.79)	0.80	4.51E-01
rs11544037	chr4	158667824	C	A	Revez et al.	0.52	(0.23-1.13)	0.40	9.95E-02	2.39	(0.93-6.1)	0.48	6.96E-02	1.64	(0.98-2.74)	0.26	5.91E-02	0.96	(0.54-1.72)	0.29	8.99E-01
rs11729497	chr4	125630228	G	C	Jiang et al.	1.23	(0.26-5.84)	0.79	7.92E-01	23.30	(3.12-176.03)	1.03	2.21E-03	2.00	(0.66-6.04)	0.56	2.20E-01	0.28	(0.09-0.88)	0.59	2.88E-02
rs11729497	chr4	125630228	G	C	Revez et al.	0.87	(0.49-1.55)	0.29	6.42E-01	1.05	(0.49-2.27)	0.39	8.92E-01	0.66	(0.44-0.99)	0.21	4.57E-02	0.64	(0.42-0.98)	0.22	4.12E-02
rs11733032	chr4	78639073	G	A	Jiang et al.	1.47	(0.17-12.8)	1.10	7.25E-01	5.72	(1.13-29.17)	0.83	3.54E-02	3.64	(1.5-8.88)	0.45	4.39E-03	1.14	(0.24-5.35)	0.79	8.72E-01
rs11733032	chr4	78639073	G	A	Revez et al.	1.28	(0.57-2.85)	0.41	5.52E-01	1.10	(0.59-2.05)	0.32	7.58E-01	1.48	(1.06-2.06)	0.17	1.99E-02	1.13	(0.64-1.99)	0.29	6.85E-01
rs34035218	chr4	37562171	C	T	Jiang et al.	2.05	(0.42-10.01)	0.81	3.73E-01	0.52	(0.09-3.02)	0.90	4.67E-01	0.26	(0.11-0.63)	0.46	3.13E-03	1.60	(0.52-4.97)	0.58	4.16E-01
rs34035218	chr4	37562171	C	T	Revez et al.	1.05	(0.58-1.89)	0.30	8.80E-01	0.71	(0.37-1.36)	0.33	3.02E-01	0.75	(0.54-1.05)	0.17	8.95E-02	1.16	(0.76-1.76)	0.21	4.88E-01
rs10069743	chr5	129088984	T	C	Jiang et al.	0.20	(0.01-3.44)	1.44	2.70E-01	0.53	(0.02-15.88)	1.72	7.14E-01	0.67	(0.11-4.2)	0.93	6.72E-01	2.43	(0.34-17.55)	1.01	3.79E-01
rs10069743	chr5	129088984	T	C	Revez et al.	0.58	(0.21-1.65)	0.53	3.10E-01	0.84	(0.23-3.05)	0.65	7.87E-01	0.89	(0.46-1.73)	0.34	7.39E-01	1.59	(0.77-3.31)	0.37	2.12E-01
rs1430204	chr5	88382815	C	T	Jiang et al.	1.99	(0.32-12.36)	0.93	4.60E-01	0.15	(0-7.65)	1.99	3.43E-01	0.92	(0.13-6.41)	0.99	9.34E-01	0.56	(0.13-2.36)	0.73	4.32E-01
rs1430204	chr5	88382815	C	T	Revez et al.	1.20	(0.6-2.36)	0.35	6.07E-01	1.06	(0.24-4.74)	0.76	9.39E-01	0.74	(0.36-1.49)	0.36	3.94E-01	0.95	(0.56-1.61)	0.27	8.49E-01
rs2170502	chr5	7911030	G	A	Jiang et al.	1.19	(0.24-5.87)	0.82	8.35E-01	0.49	(0.08-3.1)	0.94	4.50E-01	0.52	(0.2-1.33)	0.48	1.71E-01	1.94	(0.61-6.12)	0.59	2.58E-01
rs2170502	chr5	7911030	G	A	Revez et al.	0.96	(0.53-1.74)	0.30	9.00E-01	0.67	(0.34-1.32)	0.35	2.48E-01	0.78	(0.55-1.09)	0.17	1.45E-01	1.25	(0.82-1.91)	0.21	2.95E-01
rs257916	chr5	129085865	T	G	Jiang et al.	0.40	(0.08-2.03)	0.83	2.67E-01	3.29	(0.51-21.4)	0.95	2.12E-01	2.64	(1.02-6.83)	0.48	4.50E-02	1.35	(0.42-4.34)	0.60	6.15E-01
rs257916	chr5	129085865	T	G	Revez et al.	0.76	(0.42-1.4)	0.31	3.82E-01	1.20	(0.61-2.39)	0.35	5.94E-01	1.39	(0.98-1.98)	0.18	6.34E-02	1.17	(0.76-1.79)	0.22	4.84E-01
rs4276369	chr5	61330048	G	A	Jiang et al.	1.95	(0.28-13.86)	1.00	5.02E-01	0.96	(0.1-9.12)	1.14	9.73E-01	0.47	(0.15-1.52)	0.60	2.07E-01	1.20	(0.28-5.18)	0.75	8.04E-01
rs4276369	chr5	61330048	G	A	Revez et al.	1.12	(0.53-2.34)	0.38	7.67E-01	0.86	(0.38-1.95)	0.42	7.15E-01	0.75	(0.49-1.16)	0.22	1.99E-01	1.16	(0.68-1.98)	0.27	5.96E-01
rs4703608	chr5	73654711	A	G	Jiang et al.	0.47	(0.06-3.94)	1.09	4.85E-01	1.39	(0.12-16.39)	1.25	7.92E-01	3.32	(0.92-12)	0.65	6.69E-02	1.00	(0.22-4.46)	0.76	1.00E+00
rs4703608	chr5	73654711	A	G	Revez et al.	0.92	(0.42-2.01)	0.40	8.29E-01	0.69	(0.28-1.72)	0.46	4.24E-01	1.37	(0.86-2.21)	0.24	1.89E-01	1.00	(0.58-1.73)	0.28	9.91E-01
rs55811049	chr5	118988603	A	C	Jiang et al.	7.48	(0.75-74.49)	1.17	8.60E-02	1.89	(0.1-35.21)	1.50	6.71E-01	3.12	(0.88-11.06)	0.65	7.85E-02	1.51	(0.29-7.86)	0.84	6.25E-01
rs55811049	chr5	118988603	A	C	Revez et al.	1.57	(0.67-3.66)	0.43	2.96E-01	0.93	(0.31-2.75)	0.55	8.96E-01	1.81	(1.14-2.88)	0.24	1.21E-02	1.34	(0.73-2.45)	0.31	3.46E-01
rs67061065	chr5	36242110	C	T	Jiang et al.	0.46	(0.09-2.33)	0.83	3.45E-01	2.45	(0.41-14.91)	0.92	3.30E-01	1.18	(0.47-3.01)	0.48	7.22E-01	0.57	(0.18-1.83)	0.59	3.45E-01
rs67061065	chr5	36242110	C	T	Revez et al.	0.79	(0.43-1.44)	0.31	4.36E-01	1.45	(0.74-2.85)	0.34	2.80E-01	0.94	(0.67-1.32)	0.17	7.11E-01	0.69	(0.45-1.06)	0.22	9.11E-02
rs712615	chr5	116885938	T	A	Jiang et al.	2.35	(0.39-14.13)	0.91	3.49E-01	0.35	(0.07-1.8)	0.84	2.07E-01	0.99	(0.43-2.33)	0.43	9.90E-01	0.73	(0.19-2.76)	0.68	6.47E-01
rs712615	chr5	116885938	T	A	Revez et al.	1.15	(0.58-2.26)	0.35	6.90E-01	0.38	(0.21-0.69)	0.30	1.38E-03	0.87	(0.64-1.18)	0.16	3.62E-01	0.86	(0.53-1.39)	0.25	5.42E-01
rs72775970	chr5	97643953	C	T	Jiang et al.	2.09	(0.19-23.23)	1.23	5.49E-01	0.14	(0.01-2.38)	1.45	1.75E-01	0.44	(0.1-1.91)	0.75	2.74E-01	3.13	(0.58-16.81)	0.86	1.83E-01
rs72775970	chr5	97643953	C	T	Revez et al.	1.55	(0.63-3.77)	0.45	3.39E-01	0.77	(0.27-2.17)	0.53	6.17E-01	0.54	(0.31-0.92)	0.28	2.37E-02	1.31	(0.71-2.42)	0.31	3.94E-01
rs10949389	chr6	16942833	A	G	Jiang et al.	2.67	(0.49-14.5)	0.86	2.55E-01	3.24	(0.53-19.74)	0.92	2.03E-01	0.74	(0.29-1.89)	0.48	5.33E-01	1.60	(0.47-5.42)	0.62	4.54E-01
rs10949389	chr6	16942833	A	G	Revez et al.	1.19	(0.63-2.23)	0.32	5.94E-01	1.71	(0.88-3.36)	0.34	1.16E-01	0.86	(0.61-1.21)	0.18	3.85E-01	1.06	(0.67-1.66)	0.23	8.10E-01
rs12214623	chr6	113065866	T	C	Jiang et al.	0.89	(0.14-5.89)	0.96	9.07E-01	0.90	(0.11-7.35)	1.07	9.18E-01	0.87	(0.28-2.71)	0.58	8.14E-01	1.10	(0.28-4.33)	0.70	8.92E-01

rs12214623	chr6	113065866	T	C	Revez et al.	1.40 (0.69-2.83)	0.36	3.51E-01	1.21 (0.55-2.69)	0.41	6.41E-01	1.04 (0.69-1.58)	0.21	8.52E-01	1.21 (0.73-2.01)	0.26	4.59E-01
rs1782437	chr6	90425069	G	A	Jiang et al.	1.24 (0.12-12.34)	1.17	8.54E-01	5.52 (0.02-1860.55)	2.98	5.66E-01	2.16 (0.12-39.41)	1.48	6.03E-01	0.46 (0.1-2.19)	0.79	3.32E-01
rs1782437	chr6	90425069	G	A	Revez et al.	0.76 (0.32-1.82)	0.45	5.39E-01	2.00 (0.22-17.69)	1.11	5.33E-01	1.08 (0.37-3.15)	0.55	8.88E-01	1.07 (0.6-1.91)	0.29	8.18E-01
rs178399	chr6	2791449	G	A	Jiang et al.	0.64 (0.16-2.49)	0.70	5.16E-01	0.71 (0.13-4)	0.88	6.96E-01	0.91 (0.37-2.24)	0.46	8.43E-01	1.05 (0.4-2.76)	0.50	9.28E-01
rs178399	chr6	2791449	G	A	Revez et al.	0.75 (0.45-1.24)	0.26	2.61E-01	1.51 (0.79-2.87)	0.33	2.15E-01	1.01 (0.73-1.41)	0.17	9.40E-01	0.85 (0.59-1.21)	0.18	3.58E-01
rs2531804	chr6	28443526	A	G	Jiang et al.	0.93 (0.05-17.16)	1.49	9.60E-01	1.43 (0.24-8.65)	0.92	6.97E-01	1.47 (0.56-3.81)	0.49	4.33E-01	1.81 (0.22-14.82)	1.07	5.81E-01
rs2531804	chr6	28443526	A	G	Revez et al.	1.38 (0.46-4.12)	0.56	5.68E-01	0.91 (0.46-1.82)	0.35	7.98E-01	1.10 (0.78-1.57)	0.18	5.77E-01	1.00 (0.46-2.17)	0.39	9.99E-01
rs274436	chr6	104326558	C	T	Jiang et al.	0.65 (0.11-3.63)	0.88	6.20E-01	3.68 (0.56-24.14)	0.96	1.74E-01	1.06 (0.41-2.73)	0.48	8.98E-01	1.56 (0.48-5.15)	0.61	4.62E-01
rs274436	chr6	104326558	C	T	Revez et al.	0.96 (0.51-1.83)	0.33	9.12E-01	1.52 (0.76-3.04)	0.35	2.40E-01	0.99 (0.7-1.4)	0.18	9.59E-01	0.93 (0.6-1.44)	0.23	7.43E-01
rs3798259	chr6	45921171	G	T	Jiang et al.	0.75 (0.08-7.19)	1.15	8.04E-01	0.18 (0.03-1.07)	0.91	5.95E-02	0.27 (0.1-0.71)	0.49	8.01E-03	2.72 (0.51-14.4)	0.85	2.40E-01
rs3798259	chr6	45921171	G	T	Revez et al.	1.00 (0.42-2.35)	0.44	9.93E-01	1.02 (0.51-2.02)	0.35	9.53E-01	0.70 (0.49-1)	0.18	4.96E-02	1.60 (0.87-2.94)	0.31	1.33E-01
rs396360	chr6	13260221	C	T	Jiang et al.	0.74 (0.05-11.18)	1.39	8.25E-01	0.03 (0-0.69)	1.64	2.99E-02	0.39 (0.08-1.93)	0.81	2.50E-01	1.78 (0.26-12.26)	0.99	5.60E-01
rs396360	chr6	13260221	C	T	Revez et al.	1.27 (0.46-3.5)	0.52	6.41E-01	0.45 (0.14-1.47)	0.60	1.90E-01	1.36 (0.74-2.49)	0.31	3.22E-01	1.46 (0.71-2.97)	0.36	3.03E-01
rs66773803	chr6	130967385	C	T	Jiang et al.	1.20 (0.09-15.72)	1.31	8.88E-01	0.05 (0-0.91)	1.54	4.46E-02	0.08 (0.02-0.44)	0.86	3.50E-03	3.05 (0.47-19.59)	0.95	2.40E-01
rs66773803	chr6	130967385	C	T	Revez et al.	0.86 (0.33-2.22)	0.49	7.49E-01	1.39 (0.41-4.67)	0.62	5.95E-01	0.62 (0.33-1.17)	0.32	1.38E-01	1.55 (0.79-3.04)	0.34	1.99E-01
rs9347904	chr6	158032179	T	C	Jiang et al.	1.02 (0.22-4.72)	0.78	9.84E-01	1.00 (0.09-10.78)	1.21	9.97E-01	0.18 (0.05-0.58)	0.61	4.55E-03	0.84 (0.28-2.5)	0.56	7.51E-01
rs9347904	chr6	158032179	T	C	Revez et al.	0.88 (0.5-1.56)	0.29	6.59E-01	1.11 (0.46-2.66)	0.45	8.16E-01	0.65 (0.42-1.01)	0.22	5.43E-02	0.93 (0.63-1.39)	0.20	7.35E-01
rs9348121	chr6	166341524	C	A	Jiang et al.	1.66 (0.29-9.38)	0.88	5.68E-01	0.82 (0.12-5.78)	0.99	8.42E-01	0.48 (0.18-1.3)	0.51	1.46E-01	0.88 (0.25-3.08)	0.64	8.45E-01
rs9348121	chr6	166341524	C	A	Revez et al.	1.08 (0.56-2.07)	0.33	8.17E-01	1.13 (0.54-2.36)	0.38	7.47E-01	0.79 (0.54-1.14)	0.19	2.03E-01	1.30 (0.82-2.05)	0.23	2.67E-01
rs9493768	chr6	133953159	T	A	Jiang et al.	0.01 (0-4.32)	3.43	1.25E-01	0.34 (0.01-9.52)	1.71	5.32E-01	1.90 (0.33-11.12)	0.90	4.75E-01	8.56 (0.07-1039.31)	2.45	3.81E-01
rs9493768	chr6	133953159	T	A	Revez et al.	0.32 (0.03-4.01)	1.29	3.75E-01	0.62 (0.18-2.15)	0.64	4.54E-01	0.88 (0.45-1.7)	0.34	7.01E-01	2.06 (0.35-12.16)	0.91	4.26E-01
rs10232857	chr7	55533643	C	T	Jiang et al.	6.02 (0.52-70.04)	1.25	1.52E-01	0.54 (0.04-8.54)	1.40	6.64E-01	0.20 (0.05-0.8)	0.70	2.22E-02	1.04 (0.14-7.7)	1.02	9.70E-01
rs10232857	chr7	55533643	C	T	Revez et al.	1.44 (0.57-3.68)	0.48	4.43E-01	1.29 (0.47-3.59)	0.52	6.22E-01	0.74 (0.45-1.2)	0.25	2.23E-01	1.09 (0.53-2.27)	0.37	8.08E-01
rs10951263	chr7	30163611	G	A	Jiang et al.	0.76 (0.12-4.81)	0.94	7.74E-01	0.32 (0.02-6.12)	1.50	4.53E-01	0.18 (0.04-0.81)	0.76	2.53E-02	1.05 (0.28-3.92)	0.67	9.44E-01
rs10951263	chr7	30163611	G	A	Revez et al.	0.90 (0.45-1.79)	0.35	7.66E-01	0.40 (0.13-1.22)	0.57	1.08E-01	0.50 (0.29-0.86)	0.28	1.18E-02	0.98 (0.6-1.6)	0.25	9.34E-01
rs2686823	chr7	47937388	C	T	Jiang et al.	0.70 (0.14-3.43)	0.81	6.64E-01	1.09 (0.22-5.43)	0.82	9.13E-01	1.43 (0.64-3.2)	0.41	3.89E-01	1.95 (0.63-6.08)	0.58	2.47E-01
rs2686823	chr7	47937388	C	T	Revez et al.	1.09 (0.6-1.97)	0.30	7.71E-01	1.13 (0.62-2.06)	0.30	6.84E-01	1.18 (0.88-1.6)	0.15	2.66E-01	1.24 (0.81-1.88)	0.21	3.19E-01
rs2708881	chr7	47937496	C	A	Jiang et al.	2.27 (0.05-95.73)	1.91	6.67E-01	0.89 (0.12-6.83)	1.04	9.13E-01	0.64 (0.23-1.78)	0.53	3.89E-01	0.20 (0.01-2.99)	1.37	2.46E-01
rs2708881	chr7	47937496	C	A	Revez et al.	0.81 (0.2-3.28)	0.71	7.69E-01	0.85 (0.4-1.82)	0.39	6.84E-01	0.81 (0.55-1.18)	0.19	2.66E-01	0.60 (0.22-1.62)	0.50	3.17E-01
rs34571901	chr7	37038985	A	G	Jiang et al.	0.81 (0.06-11.52)	1.36	8.75E-01	0.72 (0.03-16.61)	1.61	8.36E-01	0.91 (0.19-4.32)	0.79	9.08E-01	6.12 (0.73-51.28)	1.08	9.48E-02
rs34571901	chr7	37038985	A	G	Revez et al.	0.73 (0.27-1.97)	0.50	5.39E-01	0.68 (0.2-2.28)	0.62	5.33E-01	1.04 (0.58-1.86)	0.30	8.92E-01	1.40 (0.64-3.06)	0.40	3.94E-01
rs698631	chr7	56195405	C	T	Jiang et al.	0.15 (0.02-1.24)	1.09	7.84E-02	161.92 (5.22-5074.49)	1.75	3.73E-03	2.52 (0.4-15.8)	0.94	3.25E-01	1.50 (0.31-7.17)	0.80	6.11E-01
rs698631	chr7	56195405	C	T	Revez et al.	0.63 (0.28-1.39)	0.41	2.51E-01	4.96 (1.35-18.22)	0.66	1.57E-02	1.14 (0.59-2.22)	0.34	6.97E-01	1.23 (0.7-2.18)	0.29	4.74E-01
rs1347322	chr8	102536334	G	A	Jiang et al.	0.22 (0.01-6.82)	1.75	3.87E-01	0.79 (0.1-5.93)	1.03	8.22E-01	0.67 (0.24-1.9)	0.53	4.52E-01	1.52 (0.13-18.02)	1.26	7.41E-01
rs1347322	chr8	102536334	G	A	Revez et al.	0.43 (0.12-1.57)	0.66	1.99E-01	1.11 (0.52-2.38)	0.39	7.80E-01	1.16 (0.79-1.7)	0.20	4.53E-01	1.08 (0.44-2.69)	0.46	8.65E-01
rs1389206	chr8	71012627	G	A	Jiang et al.	0.91 (0.19-4.39)	0.80	9.03E-01	0.22 (0.03-1.36)	0.93	1.04E-01	0.87 (0.33-2.3)	0.49	7.86E-01	0.36 (0.11-1.11)	0.58	7.61E-02
rs1389206	chr8	71012627	G	A	Revez et al.	1.07 (0.59-1.94)	0.30	8.13E-01	0.71 (0.36-1.4)	0.34	3.27E-01	0.92 (0.65-1.31)	0.18	6.52E-01	0.76 (0.5-1.16)	0.21	2.09E-01
rs4440604	chr8	104278607	G	A	Jiang et al.	0.27 (0.06-1.22)	0.77	8.82E-02	0.44 (0.08-2.47)	0.88	3.50E-01	0.57 (0.23-1.42)	0.46	2.31E-01	0.61 (0.21-1.83)	0.56	3.81E-01
rs4440604	chr8	104278607	G	A	Revez et al.	0.50 (0.28-0.87)	0.29	1.50E-02	1.13 (0.59-2.15)	0.33	7.11E-01	0.87 (0.63-1.22)	0.17	4.30E-01	0.76 (0.51-1.13)	0.20	1.70E-01
rs10119345	chr9	110898705	C	T	Jiang et al.	0.18 (0.02-1.84)	1.17	1.50E-01	0.33 (0.03-3.93)	1.26	3.81E-01	0.83 (0.25-2.76)	0.61	7.55E-01	2.45 (0.5-11.98)	0.81	2.68E-01
rs10119345	chr9	110898705	C	T	Revez et al.	0.68 (0.29-1.57)	0.43	3.66E-01	1.00 (0.41-2.48)	0.46	9.97E-01	1.17 (0.75-1.83)	0.23	4.83E-01	1.72 (0.95-3.11)	0.30	7.34E-02
rs10812343	chr9	2616875	G	A	Jiang et al.	0.10 (0.01-1.64)	1.44	1.06E-01	3.99 (0.67-23.84)	0.91	1.29E-01	1.72 (0.69-4.25)	0.46	2.43E-01	1.25 (0.17-9.26)	1.02	8.29E-01
rs10812343	chr9	2616875	G	A	Revez et al.	0.47 (0.16-1.35)	0.54	1.62E-01	1.15 (0.6-2.23)	0.34	6.74E-01	1.27 (0.92-1.77)	0.17	1.52E-01	0.87 (0.41-1.82)	0.38	7.06E-01
rs2297876	chr9	35604048	G	A	Jiang et al.	0.64 (0.07-5.83)	1.13	6.89E-01	0.04 (0-0.53)	1.30	1.48E-02	0.27 (0.07-1.01)	0.68	5.24E-02	3.96 (0.74-21.05)	0.85	1.07E-01
rs2297876	chr9	35604048	G	A	Revez et al.	1.64 (0.7-3.81)	0.43	2.53E-01	0.46 (0.17-1.27)	0.51	1.37E-01	0.95 (0.58-1.55)	0.25	8.30E-01	2.10 (1.15-3.86)	0.31	1.65E-02
rs72761985	chr9	113517421	G	T	Jiang et al.	13.52 (0.3-600.53)	1.94	1.78E-01	###### (7.27-386325.2	2.77	7.71E-03	10.17 (0.75-138.62)	1.33	8.13E-02	0.45 (0.03-6.88)	1.39	5.64E-01
rs72761985	chr9	113517421	G	T	Revez et al.	2.44 (0.6-9.89)	0.71	2.12E-01	7.13 (0.89-57.64)	1.06	6.41E-02	1.23 (0.47-3.24)	0.49	6.72E-01	0.58 (0.22-1.58)	0.51	2.89E-01
rs10883801	chr10	102918130	C	A	Jiang et al.	1.33 (0.25-7.03)	0.85	7.34E-01	0.15 (0.02-0.97)	0.95	4.57E-02	0.86 (0.33-2.27)	0.49	7.63E-01	1.64 (0.48-5.6)	0.63	4.31E-01
rs10883801	chr10	102918130	C	A	Revez et al.	0.91 (0.49-1.69)	0.32	7.57E-01	0.68 (0.34-1.37)	0.35	2.82E-01	1.11 (0.78-1.58)	0.18	5.70E-01	1.38 (0.88-2.18)	0.23	1.58E-01
rs10904183	chr10	4097576	A	G	Jiang et al.	7.20 (1-51.68)	1.01	4.97E-02	2.74 (0.36-21.03)	1.04	3.33E-01	1.37 (0.48-3.9)	0.53	5.53E-01	1.03 (0.23-4.56)	0.76	9.69E-01
rs10904183	chr10	4097576	A	G	Revez et al.	1.39 (0.67-2.86)	0.37	3.75E-01	1.36 (0.64-2.89)	0.39	4.29E-01	0.91 (0.62-1.33)	0.20	6.21E-01	1.14 (0.66-1.96)	0.28	6.49E-01
rs10995246	chr10	62632086	C	A	Jiang et al.	0.45 (0.09-2.34)	0.84	3.45E-01	8.83 (0.3-259.28)	1.72	2.07E-01	1.84 (0.31-10.98)	0.91	5.04E-01	1.32 (0.4-4.36)	0.61	6.45E-01
rs10995246	chr10	62632086	C	A	Revez et al.	0.65 (0.36-1.2)	0.31	1.69E-01	1.76 (0.5-6.21)	0.64	3.78E-01	1.23 (0.64-2.37)	0.33	5.32E-01	1.02 (0.66-1.58)	0.22	9.20E-01
rs12098858	chr10	67012714	T	C	Jiang et al.	1.74 (0.29-10.35)	0.91	5.44E-01	0.04 (0-0.64)	1.35	2.19E-02	0.42 (0.12-1.55)	0.66	1.94E-01	0.91 (0.25-3.3)	0.66	8.91E-01
rs12098858	chr10	67012714	T	C	Revez et al.	1.06 (0.54-2.06)	0.34	8.67E-01	0.49 (0.19-1.31)	0.50	1.56E-01	0.96 (0.6-1.55)	0.24	8.82E-01	1.14 (0.71-1.82)	0.24	5.88E-01
rs12763743	chr10	95832399	A	T	Jiang et al.	1.40 (0.15-13.45)	1.15	7.69E-01	0.96 (0.08-11.72)	1.28	9.77E-01	0.93 (0.25-3.39)	0.66	9.10E-01	0.82 (0.16-4.16)	0.83	8.15E-01
rs12763743	chr10	95832399	A	T	Revez et al.	1.52 (0.65-3.57)	0.44	3.36E-01	0.95 (0.36-2.49)	0.49	9.12E-01	0.72 (0.45-1.16)	0.24	1.73E-01	0.79 (0.44-1.43)	0.30	4.37E-01
rs1750737	chr10	11113369	A	G	Jiang et al.	5.36 (0.36-79.28)	1.37	2.22E-01	3.10 (0.22-42.59)	1.34	3.98E-01	3.58 (0.84-15.3)	0.74	8.43E-02	0.62 (0.09-4.15)	0.97	6.24E-01
rs1750737	chr10	11113369	A	G	Revez et al.	2.44 (0.88-6.72)	0.52	8.55E-02	0.78 (0.29-2.1)	0.51	6.29E-01	1.56 (0.92-2.65)	0.27	1.02E-01	0.87 (0.43-1.74)	0.35	6.92E-01
rs2387397	chr10	6348230	C	G	Jiang et al.	1.37 (0.21-8.84)	0.95	7.42E-01	0.02 (0-0.33)	1.52	7.12E-03	0.07 (0.02-0.35)	0.80	1.03E-03	1.46 (0.39-5.56)	0.68	5.76E-01
rs2387397	chr10	6348230	C	G	Revez et al.	0.97 (0.48-1.94)	0.35	9.31E-01	0.52 (0.16-1.65)	0.59	2.72E-01	0.70 (0.4-1.25)	0.29	2.34E-01	0.94 (0.57-1.54)	0.25	7.99E-01
rs3750619	chr10	110919278	G	A	Jiang et al.	7.39 (0.06-954.45)	2.48	4.20E-01	1.24 (0.05-28.77)	1.61	8.96E-01	1.55 (0.3-7.99)	0.84	6.01E-01	0.64 (0.02-18.38)	1.71	7.94E-01
rs3750619	chr10	110919278	G	A	Revez et al.	2.91 (0.48-17.67)	0.92	2.46E-01	1.86 (0.57-6.08)	0.60	3.03E-01	1.46 (0.8-2.67)	0.31	2.15E-01	1.13 (0.33-3.91)	0.63	8.42E-01
rs3793786	chr10	49535042	A	G	Jiang et al.	11.02 (1.78-68.36)	0.93	9.94E-03	2.88 (0.41-20.06)	0.99	2.87E-01	1.32 (0.48-3.62)	0.52	5.95E-01	0.41 (0.12-1.43)	0.64	1.61E-01
rs3793786	chr10	49535042	A	G	Revez et al.	1.96 (0.99-3.88)	0.35	5.19E-02	1.21 (0.58-2.54)	0.38	6.08E-01	1.00 (0.69-1.45)	0.19	9.97E-01	0.74 (0.46-1.17)	0.24	1.98E-01
rs670446	chr11	107602007	C	T	Jiang et al.	0.89 (0.08-9.83)	1.22	9.27E-01	0.65 (0.02-21.06)	1.78	8.09E-01	4.50 (0.74-27.52)	0.92	1.04E-01	0.65 (0.1-4.09)	0.94	6.50E-01
rs670446	chr11	107602007	C	T	Revez et al.	1.14 (0.47-2.74)	0.45	7.75E-01	1.61 (0.45-5.81)	0.65	4.66E-01	1.68 (0.87-3.26)	0.34	1.23E-01	1.19 (0.6-2.33)	0.34	6.21E-01
rs871699	chr11	18106132	T	C	Jiang et al.	0.15 (0.02-1.51)	1.17	1.07E-01	0.18 (0-10.38)	2.09	4.04E-01	0.36 (0.04-3.12)	1.11	3.52E-01	0.47 (0.09-2.47)	0.85	3.69E-01
rs871699	chr11	18106132	T	C	Revez et al.	0.80 (0.34-1.87)	0.43	6.08E-01	1.94 (0.4-9.4)	0.81	4.12E-01	1.15 (0.51-2.57)	0.41	7.33E-01	0.78 (0.42-1.45)	0.31	4.36E-01
rs10862993	chr12	85531560	T	C	Jiang et al.	4.81 (0.96-24.1)	0.82	5.61E-02	0.39 (0.07-2.07)	0.85	2.69E-01	1.84 (0.76-4.44)	0.45	1.75E-01	2.27 (0.72-7.19)	0.59	1.62E-01
rs10862993	chr12	85531560	T	C	Revez et al.	1.52 (0.84-2.75)	0.30	1.65E-01	0.65 (0.35-1.21)	0.32	1.77E-01	0.98 (0.71-1.36)	0.17	9.13E-01	1.37 (0.9-2.09)	0.22	1.47E-01
rs12300068	chr12	79087591	G	A	Jiang et al.	2.38 (0.25-22.9)	1.15	4.52E-01	2.27 (0.2-25.39)	1.23	5.06E-01	1.99 (0.56-7.09)	0.65	2.88E-01	0.36 (0.07-1.9)	0.85	2.29E-01
rs12300068	chr12	79087591	G	A	Revez et al.	0.96 (0.41-2.26)	0.43	9.31E-01	0.94 (0.37-2.34)	0.47	8.91E-01	1.50 (0.95-2.39)	0.24	8.54E-02	0.87 (0.47-1.6)	0.31	6.44E-01
rs2286576	chr12	4604842	T	G	Jiang et al.	1.70 (0.37-7.85)	0.78	4.97E-01	3.96 (0.6-26.32)	0.97	1.54E-01	4.30 (1.55-11.91)	0.52	4.99E-03	1.35 (0.45-4.03)	0.56	5.90E-01
rs2286576	chr12	4604842	T	G	Revez et al.	1.03 (0.58-1.83)	0.29	9.12E-01	1.35 (0.65-2.78)	0.37	4.24E-01	1.36 (0.93-1.99)	0.19	1.08E-01	1.10 (0.73-1.64)	0.21	6.57E-01
rs2638263	chr12	40152998	A	T	Jiang et al.	0.77 (0.16-3.67)	0.79	7.48E-01	1.34 (0.24-7.57)	0.88	7.39E-01	1.00 (0.41-2.49)	0.46	9.94E-01	0.72 (0.24-2.2)	0.57	5.67E-01
rs2638263	chr12	40152998	A	T	Revez et al.	0.97 (0.54-1.73)	0.30	9.07E-01	0.60 (0.31-1.13)	0.33	1.14E-01	0.84 (0.6-1.18)	0.17	3.16E-01	1.08 (0.71-1.63)	0.21	7.22E-01
rs7307718	chr12	40632248	C	T	Jiang et al.	0.52 (0.11-2.4)	0.78	4.01E-01	2.33 (0.46-11.88)	0.83	3.09E-01	0.93 (0.39-2.18)	0.44	8.62E-01	0.74 (0.25-2.21)	0.56	5.86E-01
rs7307718	chr12	40632248	C	T	Revez et al.	0.90 (0.51-1.6)	0.29	7.31E-01	1.09 (0.59-2)	0.31	7.88E-01	0.80 (0.58-1.09)	0.16	1.54E-01	0.84 (0.56-1.27)	0.21	4.09E-01
rs7309003	chr12	97358012	C	G	Jiang et al.	10.72 (2.04-56.38)	0.85	5.09E-03	0.88 (0.14-5.37)	0.92	8.89E-01	0.83 (0.33-2.13)	0.48	7.02E-01	2.93 (0.91-9.5)	0.60	7.28E-02
rs7309003	chr12	97358012	C	G	Revez et al.	1.42 (0.77-2.63)	0.31	2.60E-01	1.11 (0.56-2.19)	0.35	7.70E-01	1.06 (0.75-1.5)	0.18	7.35E-01	1.60 (1.04-2.47)	0.22	3.37E-02
rs961320	chr12	48904081	C	T	Jiang et al.	3.64 (0.64-20.56)	0.88	1.44E-01	15.69 (2.44-101.85)	0.95	3.83E-03	2.89 (1.08-7.7)	0.50	3.42E-02	1.33 (0.37-4.73)	0.65	6.59E-01
rs961320	chr12	48904081	C	T	Revez et al.	1.68 (0.88-3.21)	0.33	1.18E-01	1.56 (0.77-3.19)	0.36	2.20E-01	0.97 (0.68-1.4)	0.19	8.82E-01	0.97 (0.61-1.56)	0.24	9.15E-01
rs1923909	chr13	24124980	T	C	Jiang et al.	2.00 (0.09-44.22)	1.58	6.60E-01	1.42 (0.06-34.61)	1.64	8.32E-01	2.26 (0.41-12.65)	0.88	3.52E-01	0.30 (0.03-2.78)	1.14	2.89E-01
rs1923909	chr13	24124980	T	C	Revez et al.	1.39 (0.45-4.34)	0.58	5.65E-01	1.08 (0.33-3.52)	0.60	8.97E-01	1.36 (0.73-2.54)	0.32	3.31E-01	1.02 (0.45-2.31)	0.41	9.53E-01
rs45465203	chr13	48038078	G	A	Jiang et al.	0.18 (0.01-4.14)	1.59	2.85E-01	5.92 (0.39-88.97)	1.38	1.99E-01	1.13 (0.26-4.85)	0.75	8.74E-01	0.36 (0.04-3.31)	1.13	3.69E-01
rs45465203	chr13	48038078	G	A	Revez et al.	0.52 (0.17-1.65)	0.59	2.68E-01	1.53 (0.56-4.19)	0.51	4.06E-01	1.04 (0.61-1.76)	0.27	8.98E-01	0.65 (0.29-1.48)	0.42	3.07E-01
rs12594789	chr15	65304862	G	A	Jiang et al.	1.33 (0.22-7.95)	0.91	7.54E-01	3.87 (0.6-24.99)	0.95	1.55E-01	1.15 (0.44-3.01)	0.49	7.72E-01	0.51 (0.14-1.78)	0.64	2.89E-01
rs12594789	chr15	65304862	G	A	Revez et al.	0.87 (0.44-1.69)	0.34	6.76E-01	2.23 (1.11-4.47)	0.35	2.41E-02	1.05 (0.74-1.49)	0.18	7.95E-01	0.90 (0.57-1.43)	0.24	6.67E-01
rs3825776	chr15	58454631	T	C	Jiang et al.	0.21 (0.03-1.65)	1.06	1.36E-01	0.29 (0.02-4.8)	1.44	3.88E-01	0.35 (0.09-1.38)	0.71	1.32E-01	0.64 (0.14-3.06)	0.80	5.79E-01
rs3825776	chr15	58454631	T	C	Revez et al.	0.46 (0.21-1)	0.40	5.09E-02	0.79 (0.28-2.25)	0.53	6.60E-01	0.71 (0.43-1.18)	0.26	1.92E-01	0.97 (0.55-1.73)	0.29	9.26E-01
rs4408496	chr15	70463694	T	C	Jiang et al.	3.18 (0.03-351.94)	2.40	6.30E-01	1.75 (0.01-193.81)	2.41	8.16E-01	0.10 (0.01-1.29)	1.28	7.88E-02	2.40 (0.1-58.85)	1.63	5.92E-01
rs4408496	chr15	70463694	T	C	Revez et al.	2.65 (0.48-14.58)	0.87	2.63E-01	1.04 (0.17-6.29)	0.92	9.66E-01	0.40 (0.16-1.01)	0.47	5.33E-02	1.99 (0.62-6.44)	0.60	2.50E-01
rs58583822	chr15	59165936	G	A	Jiang et al.	1.11 (0.17-7.14)	0.95	9.14E-01	1.79 (0.2-15.49)	1.10	5.98E-01	0.98 (0.33-2.9)	0.55	9.75E-01	1.43 (0.37-5.56)	0.69	6.05E-01
rs58583822	chr15	59165936	G	A	Revez et al.	1.34 (0.67-2.7)	0.36	4.05E-01	1.06 (0.46-2.41)	0.42	8.95E-01	0.82 (0.55-1.21)	0.20	3.18E-01	1.01 (0.61-1.65)	0.25	9.80E-01
rs8035605	chr15	35756386	G	T	Jiang et al.	0.69 (0.14-3.46)	0.82	6.53E-01	0.82 (0.12-5.78)	0.99	8.46E-01	0.87 (0.34-2.27)	0.49	7.83E-01	1.28 (0.4-4.13)	0.60	6.81E-01
rs8035605	chr15	35756386	G	T	Revez et al.	0.95 (0.52-1.73)	0.31	8.62E-01	0.88 (0.42-1.82)	0.37	7.28E-01	1.00 (0.71-1.42)	0.18	9.99E-01	0.96 (0.62-1.48)	0.22	8.59E-01
rs2059292	chr16	62423133	A	T	Jiang et al.	1.73 (0.34-8.76)	0.83	5.05E-01	2.43 (0.4-14.73)	0.92	3.34E-01	2.21 (0.89-5.48)	0.46	8.67E-02	0.92 (0.28-2.94)	0.60	8.83E-01
rs2059292	chr16	62423133	A	T	Revez et al.	1.21 (0.66-2.2)	0.31	5.42E-01	2.37 (1.21-4.65)	0.34	1.18E-02	1.34 (0.95-1.88)	0.17	9.22E-02	0.92 (0.6-1.41)	0.22	7.06E-01
rs35787586	chr16	19067848	C	A	Jiang et al.	0.95 (0.09-9.84)	1.19	9.66E-01	1.11 (0.04-27.47)	1.65	9.51E-01	1.66 (0.46-5.95)	0.65	4.33E-01	0.27 (0.05-1.45)	0.86	1.27E-01
rs35787586	chr16	19067848	C	A	Revez et al.	0.90 (0.37-2.19)	0.45	8.24E-01	1.55 (0.48-5.02)	0.60	4.62E-01	1.06 (0.66-1.7)	0.24	7.98E-01	0.55 (0.3-1.03)	0.32	6.25E-02
rs11657238	chr17	48908272	G	A	Jiang et al.	0.74 (0.07-7.43)	1.17	8.02E-01	1.36 (0.18-10.22)	1.03	7.67E-01	1.70 (0.6-4.76)	0.53	3.15E-01	0.69 (0.13-3.66)	0.85	6.67E-01
rs11657238	chr17	48908272	G	A	Revez et al.	0.79 (0.34-1.86)	0.44	5.94E-01	0.56 (0.26-1.18)	0.38	1.26E-01	0.97 (0.67-1.42)	0.19	8.90E-01	0.54 (0.3-1)	0.31	5.11E-02
rs1380180	chr17	15289882	A	C	Jiang et al.	1.35 (0.28-6.44)	0.80	7.04E-01	0.47 (0.07-3.32)	0.99	4.52E-01	0.45 (0.16-1.23)	0.51	1.19E-01	1.00 (0.32-3.09)	0.58	9.94E-01
rs1380180	chr17	15289882	A	C	Revez et al.	1.16 (0.65-2.08)	0.30	6.08E-01	0.79 (0.38-1.64)	0.37	5.28E-01	0.76 (0.52-1.1)	0.19	1.43E-01	1.05 (0.69-1.6)	0.21	8.05E-01
rs34038545	chr17	47067541	G	C	Jiang et al.	0.31 (0.07-1.31)	0.73	1.11E-01	1.07 (0.19-6.14)	0.89	9.42E-01	0.87 (0.36-2.12)	0.45	7.58E-01	2.09 (0.76-5.73)	0.52	1.54E-01
rs34038545	chr17	47067541	G	C	Revez et al.	0.62 (0.36-1.06)	0.27	8.15E-02	1.03 (0.54-1.97)	0.33	9.36E-01	0.92 (0.66-1.28)	0.17	6.12E-01	1.39 (0.96-2.01)	0.19	8.27E-02

rs4796758	chr17	42017501	T	G	Jiang et al.	1.99	(0.15-25.97)	1.31	5.99E-01	2.93	(0.27-31.8)	1.22	3.77E-01	1.31	(0.39-4.37)	0.61	6.58E-01	0.36	(0.05-2.41)	0.97	2.93E-01
rs4796758	chr17	42017501	T	G	Revez et al.	1.37	(0.52-3.62)	0.50	5.26E-01	1.22	(0.5-2.95)	0.45	6.65E-01	1.07	(0.69-1.66)	0.22	7.53E-01	0.69	(0.34-1.4)	0.36	3.09E-01
rs1039143	chr18	32714710	A	G	Jiang et al.	0.32	(0.05-1.85)	0.90	2.03E-01	4.28	(0.53-34.41)	1.06	1.72E-01	1.13	(0.41-3.12)	0.52	8.15E-01	0.36	(0.11-1.23)	0.62	1.04E-01
rs1039143	chr18	32714710	A	G	Revez et al.	0.72	(0.37-1.38)	0.33	3.16E-01	0.92	(0.42-2.03)	0.40	8.42E-01	0.89	(0.62-1.29)	0.19	5.48E-01	0.82	(0.52-1.29)	0.23	3.94E-01
rs689384	chr18	56651223	C	T	Jiang et al.	1.89	(0.15-24.12)	1.30	6.23E-01	3.80	(0.28-53.35)	1.34	3.19E-01	2.61	(0.66-10.29)	0.70	1.70E-01	2.60	(0.39-17.11)	0.96	3.21E-01
rs689384	chr18	56651223	C	T	Revez et al.	1.03	(0.4-2.7)	0.49	9.48E-01	1.16	(0.44-3.09)	0.50	7.59E-01	1.30	(0.79-2.16)	0.26	2.99E-01	0.90	(0.45-1.81)	0.36	7.70E-01
rs1073378	chr20	13247424	C	T	Jiang et al.	3.02	(0.59-15.45)	0.83	1.85E-01	0.17	(0.02-1.47)	1.12	1.08E-01	0.46	(0.15-1.44)	0.58	1.85E-01	1.06	(0.32-3.44)	0.60	9.28E-01
rs1073378	chr20	13247424	C	T	Revez et al.	1.58	(0.86-2.91)	0.31	1.41E-01	0.71	(0.32-1.59)	0.41	4.12E-01	0.88	(0.58-1.33)	0.21	5.36E-01	1.37	(0.89-2.11)	0.22	1.59E-01
rs16993008	chr20	47861743	G	C	Jiang et al.	0.28	(0.03-2.5)	1.12	2.52E-01	0.61	(0.03-11.87)	1.52	7.45E-01	3.19	(0.71-14.37)	0.77	1.30E-01	0.46	(0.09-2.22)	0.81	3.31E-01
rs16993008	chr20	47861743	G	C	Revez et al.	0.92	(0.41-2.08)	0.41	8.48E-01	0.74	(0.25-2.21)	0.56	5.96E-01	1.46	(0.84-2.55)	0.28	1.80E-01	0.99	(0.55-1.78)	0.30	9.67E-01
rs62200158	chr20	62926554	C	T	Jiang et al.	0.20	(0.03-1.11)	0.89	6.54E-02	0.21	(0-14.8)	2.17	4.77E-01	0.04	(0-0.38)	1.13	4.96E-03	0.56	(0.16-1.92)	0.63	3.56E-01
rs62200158	chr20	62926554	C	T	Revez et al.	0.62	(0.33-1.2)	0.33	1.56E-01	1.52	(0.31-7.33)	0.81	6.06E-01	0.69	(0.31-1.55)	0.41	3.71E-01	0.92	(0.58-1.46)	0.23	7.30E-01
rs74364610	chr21	42557163	G	A	Jiang et al.	0.22	(0.02-3.13)	1.35	2.66E-01	2.69	(0.08-85.47)	1.77	5.76E-01	0.91	(0.17-4.98)	0.87	9.16E-01	5.96	(0.87-40.84)	0.98	6.90E-02
rs74364610	chr21	42557163	G	A	Revez et al.	0.62	(0.23-1.66)	0.50	3.43E-01	3.18	(0.9-11.25)	0.64	7.22E-02	0.89	(0.48-1.65)	0.32	7.10E-01	1.65	(0.82-3.33)	0.36	1.60E-01
rs2267393	chr22	38705614	G	C	Jiang et al.	1.52	(0.28-8.2)	0.86	6.24E-01	0.99	(0.11-8.67)	1.11	9.93E-01	0.56	(0.18-1.69)	0.57	3.05E-01	1.31	(0.4-4.33)	0.61	6.59E-01
rs2267393	chr22	38705614	G	C	Revez et al.	1.14	(0.61-2.14)	0.32	6.88E-01	1.25	(0.56-2.79)	0.41	5.78E-01	0.61	(0.41-0.92)	0.21	1.83E-02	0.98	(0.63-1.53)	0.23	9.38E-01

Abbreviations: KPNC, Kaiser Permanente Northern California; UKB, UK Biobank; FDR, false discovery rate; GIV, genetic instrumental variable; VDR-BV, vitamin D receptor binding va

[a] 25(OH)D GIVs from GWAS summary statistics from J. A. Revez, et al., Genome-wide association study identifies 143 loci associated with 25 hydroxyvitamin D concentration.

Nature Communications 11, 1–12 (2020) and X. Jiang, et al., Genome-wide association study in 79,366 European-ancestry individuals informs the genetic architecture of 25-

Table S9. Meta-analysis associations for interactions between 25(OH)D GIVs and vitamin D receptor binding variants (VDR-BV) for multiple sclerosis.

VDR-BV	chr hg19	bp hg19	effect allele	other allele	25(OH)D GIV[a]	OR	95% CI	p.value	FDR	Q	df.Q	pval.Q	I2	I2_ci
rs10797448_C	chr1	233348941	C	T	Jiang et al.	0.78	(0.37-1.67)	5.26E-01	8.71E-01	0.22	3	0.97	0.00	[0-0]
rs10797448_C	chr1	233348941	C	T	Revez et al.	0.83	(0.63-1.1)	1.91E-01	7.58E-01	0.90	3	0.82	0.00	[0-0.49]
rs10800934_G	chr1	203429502	G	C	Jiang et al.	0.77	(0.42-1.42)	4.05E-01	8.21E-01	0.73	3	0.87	0.00	[0-0.37]
rs10800934_G	chr1	203429502	G	C	Revez et al.	1.04	(0.83-1.3)	7.61E-01	9.23E-01	1.91	3	0.59	0.00	[0-0.76]
rs11263852_G	chr1	36088841	G	C	Jiang et al.	0.61	(0.14-2.6)	5.03E-01	8.67E-01	4.47	3	0.22	0.33	[0-0.76]
rs11263852_G	chr1	36088841	G	C	Revez et al.	0.84	(0.57-1.25)	3.97E-01	8.21E-01	2.05	3	0.56	0.00	[0-0.78]
rs11585739_C	chr1	230110153	C	T	Jiang et al.	0.90	(0.34-2.34)	8.21E-01	9.32E-01	3.81	3	0.28	0.21	[0-0.88]
rs11585739_C	chr1	230110153	C	T	Revez et al.	0.95	(0.72-1.25)	7.07E-01	9.00E-01	0.70	3	0.87	0.00	[0-0.34]
rs12021542_T	chr1	230981596	T	A	Jiang et al.	0.70	(0.34-1.45)	3.41E-01	7.94E-01	1.98	3	0.58	0.00	[0-0.77]
rs12021542_T	chr1	230981596	T	A	Revez et al.	1.02	(0.78-1.33)	8.90E-01	9.53E-01	2.87	3	0.41	0.00	[0-0.84]
rs12048389_T	chr1	106996101	T	C	Jiang et al.	1.81	(0.7-4.67)	2.17E-01	7.70E-01	0.11	3	0.99	0.00	[0-0]
rs12048389_T	chr1	106996101	T	C	Revez et al.	1.16	(0.82-1.63)	4.16E-01	8.21E-01	2.16	3	0.54	0.00	[0-0.79]
rs12144635_T	chr1	173277123	T	C	Jiang et al.	0.26	(0.08-0.85)	2.55E-02	5.31E-01	0.06	3	1.00	0.00	[0-0]
rs12144635_T	chr1	173277123	T	C	Revez et al.	1.20	(0.78-1.84)	4.11E-01	8.21E-01	2.98	3	0.40	0.00	[0-0.85]
rs2331903_T	chr1	180981733	T	C	Jiang et al.	1.05	(0.34-3.26)	9.31E-01	9.66E-01	4.21	3	0.24	0.29	[0-0.74]
rs2331903_T	chr1	180981733	T	C	Revez et al.	1.33	(0.76-2.33)	3.17E-01	7.94E-01	6.22	3	0.10	0.52	[0-0.84]
rs3738504_C	chr1	42767136	C	G	Jiang et al.	1.15	(0.57-2.32)	6.99E-01	9.00E-01	2.26	3	0.52	0.00	[0-0.8]
rs3738504_C	chr1	42767136	C	G	Revez et al.	1.09	(0.84-1.41)	5.10E-01	8.71E-01	0.72	3	0.87	0.00	[0-0.36]
rs4916354_C	chr1	173415688	C	T	Jiang et al.	1.18	(0.52-2.71)	6.91E-01	9.00E-01	1.25	3	0.74	0.00	[0-0.63]
rs4916354_C	chr1	173415688	C	T	Revez et al.	1.23	(0.91-1.67)	1.84E-01	7.52E-01	0.35	3	0.95	0.00	[0-0]
rs56077241_C	chr1	167715577	C	A	Jiang et al.	1.20	(0.27-5.23)	8.13E-01	9.32E-01	0.17	3	0.98	0.00	[0-0]
rs56077241_C	chr1	167715577	C	A	Revez et al.	1.08	(0.63-1.86)	7.75E-01	9.23E-01	0.64	3	0.89	0.00	[0-0.28]
rs664182_A	chr1	33127286	A	G	Jiang et al.	0.56	(0.21-1.49)	2.45E-01	7.93E-01	4.79	3	0.19	0.37	[0-0.78]
rs664182_A	chr1	33127286	A	G	Revez et al.	0.98	(0.74-1.28)	8.63E-01	9.47E-01	3.06	3	0.38	0.02	[0-0.85]
rs6687177_G	chr1	157182009	G	C	Jiang et al.	1.59	(0.54-4.66)	3.98E-01	8.21E-01	7.01	3	0.07	0.57	[0-0.86]
rs6687177_G	chr1	157182009	G	C	Revez et al.	1.22	(0.95-1.55)	1.13E-01	6.96E-01	1.62	3	0.66	0.00	[0-0.72]
rs10179357_A	chr2	205368061	A	C	Jiang et al.	1.72	(0.7-4.23)	2.33E-01	7.86E-01	1.82	3	0.61	0.00	[0-0.75]
rs10179357_A	chr2	205368061	A	C	Revez et al.	1.27	(0.79-2.06)	3.24E-01	7.94E-01	5.74	3	0.12	0.48	[0-0.83]
rs12613956_T	chr2	44244270	T	G	Jiang et al.	0.52	(0.2-1.33)	1.72E-01	7.49E-01	4.04	3	0.26	0.26	[0-0.72]
rs12613956_T	chr2	44244270	T	G	Revez et al.	0.84	(0.52-1.34)	4.54E-01	8.40E-01	7.07	3	0.07	0.58	[0-0.86]
rs12624197_C	chr2	241315322	C	G	Jiang et al.	0.77	(0.26-2.32)	6.45E-01	8.78E-01	6.97	3	0.07	0.57	[0-0.86]
rs12624197_C	chr2	241315322	C	G	Revez et al.	1.05	(0.7-1.59)	8.13E-01	9.32E-01	6.98	3	0.07	0.57	[0-0.86]
rs13021990_T	chr2	13853805	T	C	Jiang et al.	2.00	(0.5-7.98)	3.25E-01	7.94E-01	6.12	3	0.11	0.51	[0-0.84]
rs13021990_T	chr2	13853805	T	C	Revez et al.	1.29	(0.74-2.26)	3.72E-01	8.19E-01	7.22	3	0.07	0.58	[0-0.86]
rs2241876_G	chr2	233278088	G	T	Jiang et al.	0.88	(0.45-1.73)	7.07E-01	9.00E-01	0.55	3	0.91	0.00	[0-0.17]
rs2241876_G	chr2	233278088	G	T	Revez et al.	0.89	(0.69-1.14)	3.55E-01	8.04E-01	2.24	3	0.52	0.00	[0-0.79]
rs3813228_A	chr2	73780218	A	G	Jiang et al.	1.59	(0.69-3.67)	2.74E-01	7.94E-01	0.85	3	0.84	0.00	[0-0.46]
rs3813228_A	chr2	73780218	A	G	Revez et al.	0.96	(0.67-1.37)	8.18E-01	9.32E-01	3.31	3	0.35	0.09	[0-0.86]
rs4413108_C	chr2	126864471	C	T	Jiang et al.	0.46	(0.16-1.31)	1.46E-01	6.96E-01	0.70	3	0.87	0.00	[0-0.35]
rs4413108_C	chr2	126864471	C	T	Revez et al.	0.74	(0.5-1.09)	1.27E-01	6.96E-01	0.82	3	0.84	0.00	[0-0.44]
rs4670248_T	chr2	38262143	T	C	Jiang et al.	1.95	(0.87-4.41)	1.07E-01	6.96E-01	1.69	3	0.64	0.00	[0-0.73]
rs4670248_T	chr2	38262143	T	C	Revez et al.	1.11	(0.82-1.51)	4.84E-01	8.61E-01	0.36	3	0.95	0.00	[0-0]
rs55792977_T	chr2	65423730	T	G	Jiang et al.	0.93	(0.47-1.84)	8.43E-01	9.42E-01	3.52	3	0.32	0.15	[0-0.87]
rs55792977_T	chr2	65423730	T	G	Revez et al.	0.96	(0.73-1.25)	7.39E-01	9.12E-01	3.84	3	0.28	0.22	[0-0.88]
rs6436813_G	chr2	228683248	G	C	Jiang et al.	1.84	(0.88-3.85)	1.03E-01	6.96E-01	3.00	3	0.39	0.00	[0-0.85]
rs6436813_G	chr2	228683248	G	C	Revez et al.	1.14	(0.87-1.5)	3.46E-01	7.94E-01	0.91	3	0.82	0.00	[0-0.5]
rs6727980_T	chr2	20868589	T	G	Jiang et al.	1.67	(0.54-5.2)	3.72E-01	8.19E-01	1.08	3	0.78	0.00	[0-0.57]
rs6727980_T	chr2	20868589	T	G	Revez et al.	1.11	(0.73-1.68)	6.37E-01	8.76E-01	1.64	3	0.65	0.00	[0-0.72]
rs13086176_C	chr3	57100218	C	T	Jiang et al.	0.52	(0.27-1.01)	5.50E-02	6.45E-01	2.59	3	0.46	0.00	[0-0.82]
rs13086176_C	chr3	57100218	C	T	Revez et al.	0.82	(0.64-1.04)	1.02E-01	6.96E-01	2.63	3	0.45	0.00	[0-0.83]
rs13098781_G	chr3	18716927	G	A	Jiang et al.	2.06	(0.86-4.93)	1.03E-01	6.96E-01	3.01	3	0.39	0.00	[0-0.85]
rs13098781_G	chr3	18716927	G	A	Revez et al.	0.95	(0.69-1.31)	7.37E-01	9.12E-01	1.54	3	0.67	0.00	[0-0.7]
rs13324462_A	chr3	197713714	A	G	Jiang et al.	2.17	(0.74-6.37)	1.59E-01	7.07E-01	2.25	3	0.52	0.00	[0-0.8]
rs13324462_A	chr3	197713714	A	G	Revez et al.	1.49	(0.99-2.23)	5.47E-02	6.45E-01	2.47	3	0.48	0.00	[0-0.81]
rs2279908_C	chr3	44729636	C	T	Jiang et al.	1.06	(0.58-1.92)	8.54E-01	9.47E-01	1.54	3	0.67	0.00	[0-0.7]
rs2279908_C	chr3	44729636	C	T	Revez et al.	1.02	(0.81-1.27)	8.87E-01	9.53E-01	0.07	3	0.99	0.00	[0-0]
rs2574711_C	chr3	11626292	C	T	Jiang et al.	0.65	(0.19-2.22)	4.94E-01	8.61E-01	0.73	3	0.87	0.00	[0-0.37]
rs2574711_C	chr3	11626292	C	T	Revez et al.	1.34	(0.85-2.11)	2.06E-01	7.67E-01	1.44	3	0.70	0.00	[0-0.68]
rs2881514_A	chr3	16512173	A	G	Jiang et al.	2.17	(1.1-4.29)	2.52E-02	5.31E-01	2.47	3	0.48	0.00	[0-0.81]
rs2881514_A	chr3	16512173	A	G	Revez et al.	1.11	(0.86-1.42)	4.34E-01	8.28E-01	2.74	3	0.43	0.00	[0-0.83]
rs62248690_G	chr3	43181246	G	A	Jiang et al.	0.29	(0.04-1.87)	1.93E-01	7.58E-01	3.54	3	0.32	0.15	[0-0.87]
rs62248690_G	chr3	43181246	G	A	Revez et al.	0.77	(0.42-1.44)	4.21E-01	8.21E-01	1.22	3	0.75	0.00	[0-0.62]
rs62410372_A	chr3	179260754	A	G	Jiang et al.	1.45	(0.74-2.84)	2.76E-01	7.94E-01	2.39	3	0.50	0.00	[0-0.81]
rs62410372_A	chr3	179260754	A	G	Revez et al.	1.13	(0.88-1.45)	3.27E-01	7.94E-01	1.56	3	0.67	0.00	[0-0.71]
rs9818027_G	chr3	189489558	G	A	Jiang et al.	1.12	(0.38-3.27)	8.38E-01	9.41E-01	4.92	3	0.18	0.39	[0-0.79]
rs9818027_G	chr3	189489558	G	A	Revez et al.	1.11	(0.69-1.79)	6.66E-01	8.91E-01	7.06	3	0.07	0.57	[0-0.86]
rs998909_A	chr3	52771077	A	G	Jiang et al.	0.48	(0.17-1.4)	1.78E-01	7.52E-01	1.46	3	0.69	0.00	[0-0.69]
rs998909_A	chr3	52771077	A	G	Revez et al.	0.80	(0.54-1.18)	2.56E-01	7.94E-01	1.16	3	0.76	0.00	[0-0.6]
rs11132491_G	chr4	187913080	G	T	Jiang et al.	0.21	(0.03-1.56)	1.28E-01	6.96E-01	11.98	3	0.01	0.75	[0.31-0.91]
rs11132491_G	chr4	187913080	G	T	Revez et al.	0.69	(0.38-1.24)	2.09E-01	7.67E-01	7.62	3	0.05	0.61	[0-0.87]
rs11544037_C	chr4	158667824	C	A	Jiang et al.	2.07	(0.42-10.29)	3.74E-01	8.19E-01	9.22	3	0.03	0.67	[0.05-0.89]
rs11544037_C	chr4	158667824	C	A	Revez et al.	1.17	(0.66-2.06)	5.95E-01	8.76E-01	8.44	3	0.04	0.64	[0-0.88]

rs11729497_G	chr4	125630228	G	C	Jiang et al.	1.74	(0.36-8.43)	4.93E-01	8.61E-01	15.36	3	0.00	0.80	[0.49-0.93]
rs11729497_G	chr4	125630228	G	C	Revez et al.	0.72	(0.56-0.93)	1.04E-02	4.64E-01	1.82	3	0.61	0.00	[0-0.75]
rs11733032_G	chr4	78639073	G	A	Jiang et al.	2.91	(1.5-5.66)	1.58E-03	2.36E-01	2.71	3	0.44	0.00	[0-0.83]
rs11733032_G	chr4	78639073	G	A	Revez et al.	1.32	(1.03-1.69)	2.61E-02	5.31E-01	1.09	3	0.78	0.00	[0-0.58]
rs34035218_C	chr4	37562171	C	T	Jiang et al.	0.77	(0.26-2.28)	6.34E-01	8.76E-01	8.56	3	0.04	0.65	[0-0.88]
rs34035218_C	chr4	37562171	C	T	Revez et al.	0.89	(0.7-1.13)	3.35E-01	7.94E-01	3.31	3	0.35	0.09	[0-0.86]
rs10069743_T	chr5	129088984	T	C	Jiang et al.	0.83	(0.26-2.59)	7.44E-01	9.16E-01	2.20	3	0.53	0.00	[0-0.79]
rs10069743_T	chr5	129088984	T	C	Revez et al.	1.00	(0.66-1.52)	9.94E-01	9.96E-01	2.77	3	0.43	0.00	[0-0.83]
rs1430204_C	chr5	88382815	C	T	Jiang et al.	0.82	(0.32-2.12)	6.85E-01	9.00E-01	1.90	3	0.59	0.00	[0-0.76]
rs1430204_C	chr5	88382815	C	T	Revez et al.	0.95	(0.67-1.35)	7.89E-01	9.23E-01	0.96	3	0.81	0.00	[0-0.52]
rs2170502_G	chr5	7911030	G	A	Jiang et al.	0.88	(0.44-1.76)	7.08E-01	9.00E-01	3.55	3	0.31	0.16	[0-0.87]
rs2170502_G	chr5	7911030	G	A	Revez et al.	0.91	(0.7-1.19)	4.88E-01	8.61E-01	3.86	3	0.28	0.22	[0-0.88]
rs257916_T	chr5	129085865	T	G	Jiang et al.	1.57	(0.7-3.55)	2.73E-01	7.94E-01	4.51	3	0.21	0.33	[0-0.77]
rs257916_T	chr5	129085865	T	G	Revez et al.	1.19	(0.94-1.5)	1.44E-01	6.96E-01	2.85	3	0.42	0.00	[0-0.84]
rs4276369_G	chr5	61330048	G	A	Jiang et al.	0.84	(0.38-1.82)	6.53E-01	8.83E-01	1.90	3	0.59	0.00	[0-0.76]
rs4276369_G	chr5	61330048	G	A	Revez et al.	0.92	(0.69-1.22)	5.60E-01	8.71E-01	1.81	3	0.61	0.00	[0-0.75]
rs4703608_A	chr5	73654711	A	G	Jiang et al.	1.53	(0.67-3.53)	3.14E-01	7.94E-01	2.90	3	0.41	0.00	[0-0.84]
rs4703608_A	chr5	73654711	A	G	Revez et al.	1.08	(0.79-1.47)	6.24E-01	8.76E-01	2.17	3	0.54	0.00	[0-0.79]
rs55811049_A	chr5	118988603	A	C	Jiang et al.	2.76	(1.15-6.64)	2.36E-02	5.31E-01	1.34	3	0.72	0.00	[0-0.66]
rs55811049_A	chr5	118988603	A	C	Revez et al.	1.53	(1.11-2.12)	9.22E-03	4.59E-01	1.51	3	0.68	0.00	[0-0.7]
rs67061065_C	chr5	36242110	C	T	Jiang et al.	0.91	(0.49-1.7)	7.71E-01	9.23E-01	2.77	3	0.43	0.00	[0-0.83]
rs67061065_C	chr5	36242110	C	T	Revez et al.	0.88	(0.68-1.14)	3.50E-01	7.99E-01	3.60	3	0.31	0.17	[0-0.87]
rs712615_T	chr5	116885938	T	A	Jiang et al.	0.89	(0.48-1.65)	7.09E-01	9.00E-01	2.54	3	0.47	0.00	[0-0.82]
rs712615_T	chr5	116885938	T	A	Revez et al.	0.76	(0.52-1.13)	1.75E-01	7.52E-01	7.50	3	0.06	0.60	[0-0.87]
rs72775970_C	chr5	97643953	C	T	Jiang et al.	0.91	(0.25-3.34)	8.83E-01	9.51E-01	5.14	3	0.16	0.42	[0-0.8]
rs72775970_C	chr5	97643953	C	T	Revez et al.	0.92	(0.54-1.58)	7.75E-01	9.23E-01	6.42	3	0.09	0.53	[0-0.85]
rs10949389_A	chr6	16942833	A	G	Jiang et al.	1.34	(0.69-2.59)	3.88E-01	8.19E-01	3.16	3	0.37	0.05	[0-0.85]
rs10949389_A	chr6	16942833	A	G	Revez et al.	1.05	(0.81-1.37)	6.99E-01	9.00E-01	3.49	3	0.32	0.14	[0-0.87]
rs12214623_T	chr6	113065866	T	C	Jiang et al.	0.94	(0.45-1.97)	8.71E-01	9.47E-01	0.07	3	1.00	0.00	[0-0]
rs12214623_T	chr6	113065866	T	C	Revez et al.	1.16	(0.88-1.52)	2.95E-01	7.94E-01	0.57	3	0.90	0.00	[0-0.2]
rs1782437_G	chr6	90425069	G	A	Jiang et al.	0.83	(0.26-2.64)	7.58E-01	9.23E-01	1.48	3	0.69	0.00	[0-0.69]
rs1782437_G	chr6	90425069	G	A	Revez et al.	1.01	(0.66-1.55)	9.61E-01	9.83E-01	0.84	3	0.84	0.00	[0-0.45]
rs178399_G	chr6	2791449	G	A	Jiang et al.	0.88	(0.5-1.53)	6.41E-01	8.76E-01	0.40	3	0.94	0.00	[0-0]
rs178399_G	chr6	2791449	G	A	Revez et al.	0.95	(0.76-1.18)	6.20E-01	8.76E-01	3.35	3	0.34	0.11	[0-0.86]
rs2531804_A	chr6	28443526	A	G	Jiang et al.	1.45	(0.68-3.1)	3.32E-01	7.94E-01	0.13	3	0.99	0.00	[0-0]
rs2531804_A	chr6	28443526	A	G	Revez et al.	1.07	(0.81-1.42)	6.25E-01	8.76E-01	0.46	3	0.93	0.00	[0-0.01]
rs274436_C	chr6	104326558	C	T	Jiang et al.	1.28	(0.68-2.43)	4.47E-01	8.37E-01	2.07	3	0.56	0.00	[0-0.78]
rs274436_C	chr6	104326558	C	T	Revez et al.	1.02	(0.8-1.29)	8.82E-01	9.51E-01	1.48	3	0.69	0.00	[0-0.69]
rs3798259_G	chr6	45921171	G	T	Jiang et al.	0.52	(0.16-1.7)	2.77E-01	7.94E-01	6.70	3	0.08	0.55	[0-0.85]
rs3798259_G	chr6	45921171	G	T	Revez et al.	0.98	(0.66-1.46)	9.20E-01	9.60E-01	5.49	3	0.14	0.45	[0-0.82]
rs396360_C	chr6	13260221	C	T	Jiang et al.	0.47	(0.11-1.93)	2.93E-01	7.94E-01	4.88	3	0.18	0.38	[0-0.79]
rs396360_C	chr6	13260221	C	T	Revez et al.	1.21	(0.81-1.81)	3.45E-01	7.94E-01	3.05	3	0.38	0.02	[0-0.85]
rs66773803_C	chr6	130967385	C	T	Jiang et al.	0.37	(0.05-2.93)	3.45E-01	7.94E-01	10.69	3	0.01	0.72	[0.2-0.9]
rs66773803_C	chr6	130967385	C	T	Revez et al.	0.99	(0.61-1.6)	9.65E-01	9.85E-01	4.22	3	0.24	0.29	[0-0.74]
rs9347904_T	chr6	158032179	T	C	Jiang et al.	0.55	(0.22-1.38)	2.05E-01	7.67E-01	4.89	3	0.18	0.39	[0-0.79]
rs9347904_T	chr6	158032179	T	C	Revez et al.	0.83	(0.65-1.07)	1.50E-01	6.96E-01	1.99	3	0.57	0.00	[0-0.77]
rs9348121_C	chr6	166341524	C	A	Jiang et al.	0.73	(0.37-1.42)	3.56E-01	8.04E-01	1.66	3	0.65	0.00	[0-0.72]
rs9348121_C	chr6	166341524	C	A	Revez et al.	0.99	(0.77-1.27)	9.53E-01	9.79E-01	3.00	3	0.39	0.00	[0-0.85]
rs9493768_T	chr6	133953159	T	A	Jiang et al.	0.97	(0.14-6.85)	9.77E-01	9.89E-01	3.96	3	0.27	0.24	[0-0.88]
rs9493768_T	chr6	133953159	T	A	Revez et al.	0.85	(0.49-1.46)	5.57E-01	8.71E-01	1.78	3	0.62	0.00	[0-0.74]
rs10232857_C	chr7	55533643	C	T	Jiang et al.	0.75	(0.17-3.26)	7.04E-01	9.00E-01	6.08	3	0.11	0.51	[0-0.84]
rs10232857_C	chr7	55533643	C	T	Revez et al.	0.95	(0.67-1.34)	7.65E-01	9.23E-01	2.29	3	0.52	0.00	[0-0.8]
rs10951263_G	chr7	30163611	G	A	Jiang et al.	0.51	(0.21-1.24)	1.37E-01	6.96E-01	3.25	3	0.35	0.08	[0-0.86]
rs10951263_G	chr7	30163611	G	A	Revez et al.	0.70	(0.47-1.06)	9.22E-02	6.96E-01	4.75	3	0.19	0.37	[0-0.78]
rs2686823_C	chr7	47937388	C	T	Jiang et al.	1.36	(0.77-2.41)	2.85E-01	7.94E-01	1.14	3	0.77	0.00	[0-0.6]
rs2686823_C	chr7	47937388	C	T	Revez et al.	1.18	(0.96-1.45)	1.26E-01	6.96E-01	0.14	3	0.99	0.00	[0-0]
rs2708881_C	chr7	47937496	C	A	Jiang et al.	0.64	(0.28-1.5)	3.07E-01	7.94E-01	1.24	3	0.74	0.00	[0-0.63]
rs2708881_C	chr7	47937496	C	A	Revez et al.	0.79	(0.58-1.08)	1.42E-01	6.96E-01	0.34	3	0.95	0.00	[0-0]
rs34571901_A	chr7	37038985	A	G	Jiang et al.	1.41	(0.48-4.1)	5.30E-01	8.71E-01	2.48	3	0.48	0.00	[0-0.81]
rs34571901_A	chr7	37038985	A	G	Revez et al.	1.02	(0.68-1.51)	9.40E-01	9.73E-01	1.50	3	0.68	0.00	[0-0.69]
rs698631_C	chr7	56195405	C	T	Jiang et al.	2.22	(0.27-18.17)	4.58E-01	8.40E-01	11.97	3	0.01	0.75	[0.3-0.91]
rs698631_C	chr7	56195405	C	T	Revez et al.	1.24	(0.68-2.26)	4.74E-01	8.55E-01	7.13	3	0.07	0.58	[0-0.86]
rs1347322_G	chr8	102536334	G	A	Jiang et al.	0.71	(0.31-1.65)	4.24E-01	8.22E-01	0.84	3	0.84	0.00	[0-0.45]
rs1347322_G	chr8	102536334	G	A	Revez et al.	1.08	(0.79-1.47)	6.37E-01	8.76E-01	2.10	3	0.55	0.00	[0-0.78]
rs1389206_G	chr8	71012627	G	A	Jiang et al.	0.57	(0.3-1.07)	7.83E-02	6.71E-01	2.78	3	0.43	0.00	[0-0.83]
rs1389206_G	chr8	71012627	G	A	Revez et al.	0.87	(0.69-1.09)	2.19E-01	7.70E-01	1.29	3	0.73	0.00	[0-0.64]
rs4440604_G	chr8	104278607	G	A	Jiang et al.	0.50	(0.28-0.91)	2.42E-02	5.31E-01	0.90	3	0.83	0.00	[0-0.49]
rs4440604_G	chr8	104278607	G	A	Revez et al.	0.78	(0.6-1.03)	7.71E-02	6.71E-01	4.18	3	0.24	0.28	[0-0.73]
rs10119345_C	chr9	110898705	C	T	Jiang et al.	0.78	(0.29-2.13)	6.26E-01	8.76E-01	3.96	3	0.27	0.24	[0-0.88]
rs10119345_C	chr9	110898705	C	T	Revez et al.	1.18	(0.85-1.64)	3.33E-01	7.94E-01	3.34	3	0.34	0.10	[0-0.86]
rs10812343_G	chr9	2616875	G	A	Jiang et al.	1.39	(0.47-4.1)	5.48E-01	8.71E-01	4.85	3	0.18	0.38	[0-0.79]
rs10812343_G	chr9	2616875	G	A	Revez et al.	1.07	(0.78-1.48)	6.69E-01	8.92E-01	3.63	3	0.30	0.17	[0-0.87]
rs2297876_G	chr9	35604048	G	A	Jiang et al.	0.46	(0.08-2.64)	3.88E-01	8.19E-01	10.38	3	0.02	0.71	[0.18-0.9]
rs2297876_G	chr9	35604048	G	A	Revez et al.	1.17	(0.66-2.09)	5.85E-01	8.76E-01	8.07	3	0.04	0.63	[0-0.87]
rs72761985_G	chr9	113517421	G	T	Jiang et al.	9.68	(0.6-154.81)	1.09E-01	6.96E-01	7.87	3	0.05	0.62	[0-0.87]
rs72761985_G	chr9	113517421	G	T	Revez et al.	1.44	(0.6-3.46)	4.20E-01	8.21E-01	5.82	3	0.12	0.48	[0-0.83]
rs10883801_C	chr10	102918130	C	A	Jiang et al.	0.85	(0.36-1.99)	7.05E-01	9.00E-01	4.70	3	0.20	0.36	[0-0.78]
rs10883801_C	chr10	102918130	C	A	Revez et al.	1.08	(0.84-1.38)	5.54E-01	8.71E-01	3.16	3	0.37	0.05	[0-0.85]

rs10904183_A	chr10	4097576	A	G	Jiang et al.	1.76	(0.85-3.66)	1.31E-01	6.96E-01	2.86	3	0.41	0.00 [0-0.84]
rs10904183_A	chr10	4097576	A	G	Revez et al.	1.07	(0.82-1.4)	6.24E-01	8.76E-01	1.62	3	0.65	0.00 [0-0.72]
rs10995246_C	chr10	62632086	C	A	Jiang et al.	1.21	(0.53-2.76)	6.47E-01	8.78E-01	2.94	3	0.40	0.00 [0-0.84]
rs10995246_C	chr10	62632086	C	A	Revez et al.	0.98	(0.72-1.34)	9.13E-01	9.59E-01	3.05	3	0.38	0.02 [0-0.85]
rs12098858_T	chr10	67012714	T	C	Jiang et al.	0.55	(0.18-1.71)	3.03E-01	7.94E-01	5.72	3	0.13	0.48 [0-0.83]
rs12098858_T	chr10	67012714	T	C	Revez et al.	0.98	(0.74-1.31)	9.16E-01	9.59E-01	2.34	3	0.50	0.00 [0-0.8]
rs12763743_A	chr10	95832399	A	T	Jiang et al.	0.96	(0.4-2.28)	9.22E-01	9.61E-01	0.14	3	0.99	0.00 [0-0]
rs12763743_A	chr10	95832399	A	T	Revez et al.	0.85	(0.61-1.17)	3.10E-01	7.94E-01	2.37	3	0.50	0.00 [0-0.81]
rs1750737_A	chr10	11113369	A	G	Jiang et al.	2.32	(0.87-6.19)	9.35E-02	6.96E-01	2.61	3	0.45	0.00 [0-0.82]
rs1750737_A	chr10	11113369	A	G	Revez et al.	1.27	(0.81-1.99)	3.04E-01	7.94E-01	4.20	3	0.24	0.29 [0-0.74]
rs2387397_C	chr10	6348230	C	G	Jiang et al.	0.27	(0.04-1.97)	1.99E-01	7.67E-01	14.21	3	0.00	0.79 [0.43-0.92]
rs2387397_C	chr10	6348230	C	G	Revez et al.	0.83	(0.6-1.14)	2.46E-01	7.93E-01	1.35	3	0.72	0.00 [0-0.66]
rs3750619_G	chr10	110919278	G	A	Jiang et al.	1.46	(0.4-5.3)	5.63E-01	8.71E-01	0.67	3	0.88	0.00 [0-0.32]
rs3750619_G	chr10	110919278	G	A	Revez et al.	1.54	(0.96-2.47)	7.61E-02	6.71E-01	0.84	3	0.84	0.00 [0-0.45]
rs3793786_A	chr10	49535042	A	G	Jiang et al.	1.75	(0.49-6.18)	3.86E-01	8.19E-01	9.18	3	0.03	0.67 [0.05-0.89]
rs3793786_A	chr10	49535042	A	G	Revez et al.	1.08	(0.74-1.55)	6.99E-01	9.00E-01	5.66	3	0.13	0.47 [0-0.82]
rs670446_C	chr11	107602007	C	T	Jiang et al.	1.38	(0.47-4.07)	5.57E-01	8.71E-01	2.58	3	0.46	0.00 [0-0.82]
rs670446_C	chr11	107602007	C	T	Revez et al.	1.37	(0.92-2.04)	1.17E-01	6.96E-01	0.78	3	0.85	0.00 [0-0.41]
rs871699_T	chr11	18106132	T	C	Jiang et al.	0.31	(0.1-0.94)	3.86E-02	6.03E-01	0.69	3	0.88	0.00 [0-0.33]
rs871699_T	chr11	18106132	T	C	Revez et al.	0.92	(0.61-1.39)	7.04E-01	9.00E-01	1.51	3	0.68	0.00 [0-0.7]
rs10862993_T	chr12	85531560	T	C	Jiang et al.	1.78	(0.8-3.96)	1.58E-01	7.07E-01	4.82	3	0.19	0.38 [0-0.79]
rs10862993_T	chr12	85531560	T	C	Revez et al.	1.09	(0.8-1.49)	5.93E-01	8.76E-01	5.34	3	0.15	0.44 [0-0.81]
rs12300068_G	chr12	79087591	G	A	Jiang et al.	1.31	(0.53-3.21)	5.60E-01	8.71E-01	3.20	3	0.36	0.06 [0-0.86]
rs12300068_G	chr12	79087591	G	A	Revez et al.	1.15	(0.84-1.58)	3.89E-01	8.19E-01	2.46	3	0.48	0.00 [0-0.81]
rs2286576_T	chr12	4604842	T	G	Jiang et al.	2.47	(1.31-4.65)	4.97E-03	4.45E-01	2.78	3	0.43	0.00 [0-0.83]
rs2286576_T	chr12	4604842	T	G	Revez et al.	1.21	(0.95-1.53)	1.18E-01	6.96E-01	0.99	3	0.80	0.00 [0-0.54]
rs2638263_A	chr12	40152998	A	T	Jiang et al.	0.91	(0.5-1.66)	7.55E-01	9.22E-01	0.44	3	0.93	0.00 [0-0]
rs2638263_A	chr12	40152998	A	T	Revez et al.	0.89	(0.71-1.11)	2.84E-01	7.94E-01	2.50	3	0.47	0.00 [0-0.82]
rs7307718_C	chr12	40632248	C	T	Jiang et al.	0.90	(0.51-1.6)	7.19E-01	9.04E-01	1.94	3	0.59	0.00 [0-0.76]
rs7307718_C	chr12	40632248	C	T	Revez et al.	0.86	(0.69-1.06)	1.50E-01	6.96E-01	0.83	3	0.84	0.00 [0-0.45]
rs7309003_C	chr12	97358012	C	G	Jiang et al.	2.06	(0.68-6.26)	2.04E-01	7.67E-01	8.29	3	0.04	0.64 [0-0.88]
rs7309003_C	chr12	97358012	C	G	Revez et al.	1.25	(0.99-1.58)	5.81E-02	6.45E-01	2.39	3	0.50	0.00 [0-0.81]
rs961320_C	chr12	48904081	C	T	Jiang et al.	3.20	(1.35-7.62)	8.39E-03	4.59E-01	4.65	3	0.20	0.36 [0-0.78]
rs961320_C	chr12	48904081	C	T	Revez et al.	1.12	(0.87-1.46)	3.78E-01	8.19E-01	3.23	3	0.36	0.07 [0-0.86]
rs1923909_T	chr13	24124980	T	C	Jiang et al.	1.21	(0.38-3.86)	7.50E-01	9.19E-01	2.13	3	0.55	0.00 [0-0.78]
rs1923909_T	chr13	24124980	T	C	Revez et al.	1.23	(0.8-1.87)	3.41E-01	7.94E-01	0.39	3	0.94	0.00 [0-0]
rs45465203_G	chr13	48038078	G	A	Jiang et al.	0.89	(0.27-2.94)	8.51E-01	9.47E-01	3.59	3	0.31	0.17 [0-0.87]
rs45465203_G	chr13	48038078	G	A	Revez et al.	0.92	(0.63-1.35)	6.58E-01	8.86E-01	2.78	3	0.43	0.00 [0-0.83]
rs12594789_G	chr15	65304862	G	A	Jiang et al.	1.10	(0.55-2.21)	7.89E-01	9.23E-01	3.26	3	0.35	0.08 [0-0.86]
rs12594789_G	chr15	65304862	G	A	Revez et al.	1.10	(0.79-1.55)	5.67E-01	8.71E-01	5.17	3	0.16	0.42 [0-0.8]
rs3825776_T	chr15	58454631	T	C	Jiang et al.	0.38	(0.16-0.91)	2.99E-02	5.59E-01	0.82	3	0.84	0.00 [0-0.44]
rs3825776_T	chr15	58454631	T	C	Revez et al.	0.74	(0.53-1.02)	6.56E-02	6.53E-01	2.33	3	0.51	0.00 [0-0.8]
rs4408496_T	chr15	70463694	T	C	Jiang et al.	0.61	(0.1-3.66)	5.87E-01	8.76E-01	3.25	3	0.36	0.08 [0-0.86]
rs4408496_T	chr15	70463694	T	C	Revez et al.	1.09	(0.41-2.85)	8.67E-01	9.47E-01	6.24	3	0.10	0.52 [0-0.84]
rs58583822_G	chr15	59165936	G	A	Jiang et al.	1.19	(0.58-2.46)	6.35E-01	8.76E-01	0.33	3	0.95	0.00 [0-0]
rs58583822_G	chr15	59165936	G	A	Revez et al.	0.96	(0.73-1.25)	7.64E-01	9.23E-01	1.62	3	0.66	0.00 [0-0.72]
rs8035605_G	chr15	35756386	G	T	Jiang et al.	0.94	(0.5-1.77)	8.40E-01	9.41E-01	0.44	3	0.93	0.00 [0-0]
rs8035605_G	chr15	35756386	G	T	Revez et al.	0.97	(0.77-1.22)	7.82E-01	9.23E-01	0.11	3	0.99	0.00 [0-0]
rs2059292_A	chr16	62423133	A	T	Jiang et al.	1.69	(0.91-3.13)	9.54E-02	6.96E-01	1.55	3	0.67	0.00 [0-0.7]
rs2059292_A	chr16	62423133	A	T	Revez et al.	1.30	(0.93-1.81)	1.24E-01	6.96E-01	5.61	3	0.13	0.46 [0-0.82]
rs35787586_C	chr16	19067848	C	A	Jiang et al.	0.89	(0.36-2.17)	7.94E-01	9.24E-01	2.88	3	0.41	0.00 [0-0.84]
rs35787586_C	chr16	19067848	C	A	Revez et al.	0.89	(0.6-1.3)	5.40E-01	8.71E-01	3.66	3	0.30	0.18 [0-0.87]
rs11657238_G	chr17	48908272	G	A	Jiang et al.	1.25	(0.58-2.66)	5.69E-01	8.71E-01	1.02	3	0.80	0.00 [0-0.55]
rs11657238_G	chr17	48908272	G	A	Revez et al.	0.76	(0.56-1.04)	8.68E-02	6.95E-01	3.43	3	0.33	0.13 [0-0.87]
rs1380180_A	chr17	15289882	A	C	Jiang et al.	0.70	(0.37-1.33)	2.77E-01	7.94E-01	1.96	3	0.58	0.00 [0-0.77]
rs1380180_A	chr17	15289882	A	C	Revez et al.	0.91	(0.72-1.15)	4.31E-01	8.24E-01	2.25	3	0.52	0.00 [0-0.8]
rs34038545_G	chr17	47067541	G	C	Jiang et al.	0.96	(0.45-2.02)	9.12E-01	9.59E-01	4.67	3	0.20	0.36 [0-0.78]
rs34038545_G	chr17	47067541	G	C	Revez et al.	0.98	(0.7-1.35)	8.79E-01	9.51E-01	6.31	3	0.10	0.52 [0-0.84]
rs4796758_T	chr17	42017501	T	G	Jiang et al.	1.17	(0.48-2.81)	7.33E-01	9.09E-01	2.24	3	0.52	0.00 [0-0.8]
rs4796758_T	chr17	42017501	T	G	Revez et al.	1.02	(0.74-1.41)	8.94E-01	9.56E-01	1.71	3	0.64	0.00 [0-0.73]
rs1039143_A	chr18	32714710	A	G	Jiang et al.	0.77	(0.29-2.06)	6.06E-01	8.76E-01	5.56	3	0.14	0.46 [0-0.82]
rs1039143_A	chr18	32714710	A	G	Revez et al.	0.85	(0.66-1.09)	1.87E-01	7.55E-01	0.39	3	0.94	0.00 [0-0]
rs689384_C	chr18	56651223	C	T	Jiang et al.	2.62	(1.01-6.75)	4.66E-02	6.33E-01	0.14	3	0.99	0.00 [0-0]
rs689384_C	chr18	56651223	C	T	Revez et al.	1.14	(0.8-1.61)	4.77E-01	8.55E-01	0.76	3	0.86	0.00 [0-0.39]
rs1073378_C	chr20	13247424	C	T	Jiang et al.	0.78	(0.29-2.1)	6.17E-01	8.76E-01	5.61	3	0.13	0.47 [0-0.82]
rs1073378_C	chr20	13247424	C	T	Revez et al.	1.11	(0.8-1.54)	5.28E-01	8.71E-01	4.56	3	0.21	0.34 [0-0.77]
rs16993008_G	chr20	47861743	G	C	Jiang et al.	0.82	(0.25-2.73)	7.51E-01	9.19E-01	4.61	3	0.20	0.35 [0-0.77]
rs16993008_G	chr20	47861743	G	C	Revez et al.	1.10	(0.78-1.55)	5.77E-01	8.73E-01	1.81	3	0.61	0.00 [0-0.75]
rs62200158_C	chr20	62926554	C	T	Jiang et al.	0.22	(0.07-0.71)	1.15E-02	4.67E-01	4.20	3	0.24	0.29 [0-0.74]
rs62200158_C	chr20	62926554	C	T	Revez et al.	0.81	(0.58-1.13)	2.17E-01	7.70E-01	1.67	3	0.64	0.00 [0-0.73]
rs74364610_G	chr21	42557163	G	A	Jiang et al.	1.42	(0.36-5.61)	6.21E-01	8.76E-01	4.41	3	0.22	0.32 [0-0.76]
rs74364610_G	chr21	42557163	G	A	Revez et al.	1.21	(0.68-2.15)	5.26E-01	8.71E-01	5.69	3	0.13	0.47 [0-0.83]
rs2267393_G	chr22	38705614	G	C	Jiang et al.	0.94	(0.47-1.87)	8.51E-01	9.47E-01	1.45	3	0.69	0.00 [0-0.68]
rs2267393_G	chr22	38705614	G	C	Revez et al.	0.89	(0.64-1.24)	4.91E-01	8.61E-01	4.63	3	0.20	0.35 [0-0.77]

Abbreviations: KPNC, Kaiser Permanente Northern California; UKB, UK Biobank; FDR, false discovery rate; GIV, genetic instrumental variable; VDR-BV, vitamin D receptor binding variant

[a] 25(OH)D GIVs from GWAS summary statistics from J. A. Revez, et al., Genome-wide association study identifies 143 loci associated with 25 hydroxyvitamin D concentration. Nature Communications 11, 1–12 (2020) and X. Jiang, et al., Genome-wide association study in 79,366 European-ancestry individuals informs the genetic architecture of 25-hydroxyvitamin D
